# Capturing Wheat Phenotypes at the Genome Level

**DOI:** 10.3389/fpls.2022.851079

**Published:** 2022-07-04

**Authors:** Babar Hussain, Bala A. Akpınar, Michael Alaux, Ahmed M. Algharib, Deepmala Sehgal, Zulfiqar Ali, Gudbjorg I. Aradottir, Jacqueline Batley, Arnaud Bellec, Alison R. Bentley, Halise B. Cagirici, Luigi Cattivelli, Fred Choulet, James Cockram, Francesca Desiderio, Pierre Devaux, Munevver Dogramaci, Gabriel Dorado, Susanne Dreisigacker, David Edwards, Khaoula El-Hassouni, Kellye Eversole, Tzion Fahima, Melania Figueroa, Sergio Gálvez, Kulvinder S. Gill, Liubov Govta, Alvina Gul, Goetz Hensel, Pilar Hernandez, Leonardo Abdiel Crespo-Herrera, Amir Ibrahim, Benjamin Kilian, Viktor Korzun, Tamar Krugman, Yinghui Li, Shuyu Liu, Amer F. Mahmoud, Alexey Morgounov, Tugdem Muslu, Faiza Naseer, Frank Ordon, Etienne Paux, Dragan Perovic, Gadi V. P. Reddy, Jochen Christoph Reif, Matthew Reynolds, Rajib Roychowdhury, Jackie Rudd, Taner Z. Sen, Sivakumar Sukumaran, Bahar Sogutmaz Ozdemir, Vijay Kumar Tiwari, Naimat Ullah, Turgay Unver, Selami Yazar, Rudi Appels, Hikmet Budak

**Affiliations:** ^1^Department of Biological Sciences, Middle East Technical University, Ankara, Turkey; ^2^Department of Biotechnology, Faculty of Life Sciences, University of Central Punjab, Lahore, Pakistan; ^3^Montana BioAgriculture, Inc., Missoula, MT, United States; ^4^Université Paris-Saclay, INRAE, URGI, Versailles, France; ^5^Department of Environment and Bio-Agriculture, Faculty of Agriculture, Al-Azhar University, Cairo, Egypt; ^6^International Maize and Wheat Improvement Center (CIMMYT), Texcoco, Mexico; ^7^Institute of Plant Breeding and Biotechnology, MNS University of Agriculture, Multan, Pakistan; ^8^Department of Pathology, The National Institute of Agricultural Botany, Cambridge, United Kingdom; ^9^School of Biological Sciences and Institute of Agriculture, University of Western Australia, Perth, WA, Australia; ^10^French Plant Genomic Resource Center, INRAE-CNRGV, Castanet Tolosan, France; ^11^Crop Improvement and Genetics Research, USDA, Agricultural Research Service, Albany, CA, United States; ^12^Council for Agricultural Research and Economics-Research Centre for Genomics and Bioinformatics, Fiorenzuola d’Arda, Italy; ^13^French National Research Institute for Agriculture, Food and the Environment, INRAE, GDEC, Clermont-Ferrand, France; ^14^The John Bingham Laboratory, The National Institute of Agricultural Botany, Cambridge, United Kingdom; ^15^Research & Innovation, Florimond Desprez Group, Cappelle-en-Pévèle, France; ^16^USDA, Agricultural Research Service, Edward T. Schafer Agricultural Research Center, Fargo, ND, United States; ^17^Department of Bioquímica y Biología Molecular, Campus Rabanales C6-1-E17, Campus de Excelencia Internacional Agroalimentario (ceiA3), Universidad de Córdoba, Córdoba, Spain; ^18^University of Western Australia, Perth, WA, Australia; ^19^State Plant Breeding Institute, The University of Hohenheim, Stuttgart, Germany; ^20^International Wheat Genome Sequencing Consortium (IWGSC), Bethesda, MD, United States; ^21^Institute of Evolution and Department of Environmental and Evolutionary Biology, University of Haifa, Haifa, Israel; ^22^Commonwealth Scientific and Industrial Research Organization, Agriculture and Food, Canberra, ACT, Australia; ^23^Department of Languages and Computer Science, ETSI Informática, Campus de Teatinos, Universidad de Málaga, Andalucía Tech, Málaga, Spain; ^24^Department of Crop Science, Washington State University, Pullman, WA, United States; ^25^Atta-ur-Rahman School of Applied Biosciences, National University of Sciences and Technology, Islamabad, Pakistan; ^26^Center of Plant Genome Engineering, Heinrich-Heine-Universität, Düsseldorf, Germany; ^27^Division of Molecular Biology, Centre of Region Haná for Biotechnological and Agriculture Research, Czech Advanced Technology and Research Institute, Palacký University, Olomouc, Czechia; ^28^Institute for Sustainable Agriculture (IAS-CSIC), Consejo Superior de Investigaciones Científicas (CSIC), Córdoba, Spain; ^29^Crop and Soil Science, Texas A&M University, College Station, TX, United States; ^30^Global Crop Diversity Trust, Bonn, Germany; ^31^KWS SAAT SE & Co. KGaA, Einbeck, Germany; ^32^Department of Plant Pathology, Faculty of Agriculture, Assiut University, Assiut, Egypt; ^33^Food and Agriculture Organization of the United Nations, Riyadh, Saudi Arabia; ^34^Molecular Biology, Genetics and Bioengineering, Sabanci University, Istanbul, Turkey; ^35^Institute for Resistance Research and Stress Tolerance, Julius Kühn Institute, Quedlinburg, Germany; ^36^USDA-Agricultural Research Service, Southern Insect Management Research Unit, Stoneville, MS, United States; ^37^Leibniz Institute of Plant Genetics and Crop Plant Research (IPK), Gatersleben, Germany; ^38^Department of Genetics and Bioengineering, Yeditepe University, Istanbul, Turkey; ^39^University of Maryland, Baltimore, MD, United States; ^40^Institute of Biological Sciences (IBS), Gomal University, D. I. Khan, Pakistan; ^41^Ficus Biotechnology, Ostim Teknopark, Ankara, Turkey; ^42^General Directorate of Research, Ministry of Agriculture, Ankara, Turkey; ^43^Murdoch University, Perth, WA, Australia

**Keywords:** Wheat, genome-wide association, quantitative trait locus mapping, abiotic-stress tolerance, genomic selection, QTL cloning, disease resistance, CRISPR/Cas9

## Abstract

Recent technological advances in next-generation sequencing (NGS) technologies have dramatically reduced the cost of DNA sequencing, allowing species with large and complex genomes to be sequenced. Although bread wheat (*Triticum aestivum* L.) is one of the world’s most important food crops, efficient exploitation of molecular marker-assisted breeding approaches has lagged behind that achieved in other crop species, due to its large polyploid genome. However, an international public–private effort spanning 9 years reported over 65% draft genome of bread wheat in 2014, and finally, after more than a decade culminated in the release of a gold-standard, fully annotated reference wheat-genome assembly in 2018. Shortly thereafter, in 2020, the genome of assemblies of additional 15 global wheat accessions was released. As a result, wheat has now entered into the pan-genomic era, where basic resources can be efficiently exploited. Wheat genotyping with a few hundred markers has been replaced by genotyping arrays, capable of characterizing hundreds of wheat lines, using thousands of markers, providing fast, relatively inexpensive, and reliable data for exploitation in wheat breeding. These advances have opened up new opportunities for marker-assisted selection (MAS) and genomic selection (GS) in wheat. Herein, we review the advances and perspectives in wheat genetics and genomics, with a focus on key traits, including grain yield, yield-related traits, end-use quality, and resistance to biotic and abiotic stresses. We also focus on reported candidate genes cloned and linked to traits of interest. Furthermore, we report on the improvement in the aforementioned quantitative traits, through the use of (i) clustered regularly interspaced short-palindromic repeats/CRISPR-associated protein 9 (CRISPR/Cas9)-mediated gene-editing and (ii) positional cloning methods, and of genomic selection. Finally, we examine the utilization of genomics for the next-generation wheat breeding, providing a practical example of using *in silico* bioinformatics tools that are based on the wheat reference-genome sequence.

## The 17 Gbp Wheat Genome: Challenges and Opportunities

Wheat (*Triticum aestivum* L.) is the key crop for feeding the Earth’ growing population, remaining a staple food in many regions of the world. It is cultivated on more than 220 million hectares worldwide, and global production exceeds 749 million tons annually.^1^ Bread wheat is a hexaploid species (2n = 6x = 42, genome AABBDD) that evolved *via* natural hybridization between tetraploid domesticated wheat *T. turgidum* ssp. *dicoccum* (contributed the AA and BB sub-genomes) and the wild grass species *Aegilops tauschii* (DD sub-genome), followed by the domestication of the resulting hexaploid spelt wheat (*T. spelta*; Petersen et al., 2006). The wheat genome is ~17 Gbp in size and contains a high degree of complexity, particularly in terms of chromosomal duplications and rearrangements, and the very high percentage of repetitive sequences (IWGSC, 2014; Akpinar et al., 2015).

Wheat breeding targets are numerous and varied, given the wide geographic area across which wheat is grown. However, the principal common targets are grain yield (GY), quality determinants, and tolerance to biotic and abiotic stresses. The complexity of the wheat genome makes improving qualitative and quantitative traits through molecular approaches challenging. An example of this is drought tolerance, which is conferred by diverse signaling molecules, including micro-RNA (miRNA), transcriptional factors, quantitative-trait loci (QTL), transcripts, proteomes, ionomes, and other metabolites, resulting in a complex signaling cascade for the control of traits such as abiotic stress (Budak et al., 2015). Furthermore, multiple genes are involved in the production and regulation of these molecules, which leads to a complex signaling cascade, responsible for conferring abiotic/biotic-stress tolerance. Hence, knowledge of the sequence, as well as the precise location, annotation, and casual polymorphisms of the genes involved is vital for utilizing the genomic data in breeding programs, aimed at achieving specific and desired traits or phenotypes.

Due to its large genome size in comparison with other major crops with smaller genomes, efforts to sequence and annotate the wheat genome have been extremely time-consuming, often involving sequencing of individual chromosomes (Berkman et al., 2011, 2012). Thus, the International Wheat Genome Sequencing Consortium (IWGSC) reported a draft sequence of bread wheat (cv. Chinese Spring; CS) in 2014, derived from sequencing flow-sorted chromosomes/chromosome arms. The draft assembly totaled 12.7 Gbp, comprising 124,201 gene loci distributed across A, B, and D sub-genomes (IWGSC, 2014). However, this assembly contained only approximately three-quarters of the whole wheat genome. Furthermore, the genome sequences of the chromosomes/chromosome arms were fragmented with many gaps as well as many incomplete, absent, or incorrectly assigned genes, making it hard for scientists to find and elucidate specific genes (Berkman et al., 2013; IWGSC, 2014; Lai et al., 2015). Despite the incompleteness of this version, it was highly useful for breeders, as it provided valuable information at the chromosomal/chromosome-arm level. A draft whole-genome sequence of wheat was obtained by combining long Pacific Biosciences (PacBio) reads (>10,000 bases long) with short (150 bp) Illumina reads, with 15.34 Gbp and an average contig size of 0.23 Mbp (Zimin et al., 2017). Low-coverage sequence data for 16 varieties were released in 2012 and used as the basis for the first draft wheat pan-genome (Edwards et al., 2012). This study highlighted the fact that, due to gene presence/absence variation, a single reference does not represent the gene content of the species (Bayer et al., 2020; Danilevicz et al., 2020; Golicz et al., 2020).

The high-quality reference sequence of wheat genome was achieved by the International Wheat Genome Sequencing Consortium in several steps. A whole-genome sequence based on Illumina technology and a draft assembly was released in 2016 (IWGSC WGS v0.4), which was comprised of Illumina short sequence reads assembled with NRGene’s DeNovoMagic (Appels et al., 2018).^2^ This was then combined with physical maps of the chromosome/chromosome arm and other genomic resources that had been developed over 13 years by numerous laboratories around the world, to develop IWGSC RefSeq v1.0. In 2018, the fully annotated reference-genome assembly was released (IWGSC RefSeqv1.1; Appels et al., 2018), with the precise location and annotation of 107,891 high-confidence genes and more than 4 million molecular markers along the 21 chromosomes. The chromosome-scale assembly covered approximately 94% of the bread-wheat genome (cv. Chinese Spring), with a total assembly size of 14.5 Gbp. A key feature of this new genome assembly was the long scaffolds, of which 90% were larger than 4.1 Mbp, the longest super scaffold being 166 Mbp (i.e., larger than the 135 Mbp *Arabidopsis thaliana* genome and half the size of the rice genome; Appels et al., 2018). Accordingly, RefSeqv1.0, with the highest sequence contiguity, has become a tool for wheat genomics and breeding activities worldwide. In 2020, the release of genome assemblies for 15 additional wheat accessions, with diverse origins across the globe (Walkowiak et al., 2020), has further consolidated the position of wheat in the genomics era, providing additional resources to underpin breeding strategies. The availability of multiple, high-quality genome assemblies for wheat has highlighted the genomic diversity present in the global breeding program. Introgressions from wild relatives, structural rearrangements, and variation in gene content, originating from various breeding efforts aimed for diverse and multiple traits have contributed to wheat genomic diversity (Walkowiak et al., 2020). An advantage of having multiple assemblies is that it enables the discovery of new sequences and genes that were not present in previous versions of the wheat genome, thus creating new opportunities to identify, characterize, and exploit the beneficial alleles/haplotypes present for wheat improvement. IWGSC RefSeq v2.0 available since 25 July 2019, as shown at “IWGSC RefSeq v2.0 now available at URGI.”^3^

**Figure 1:**
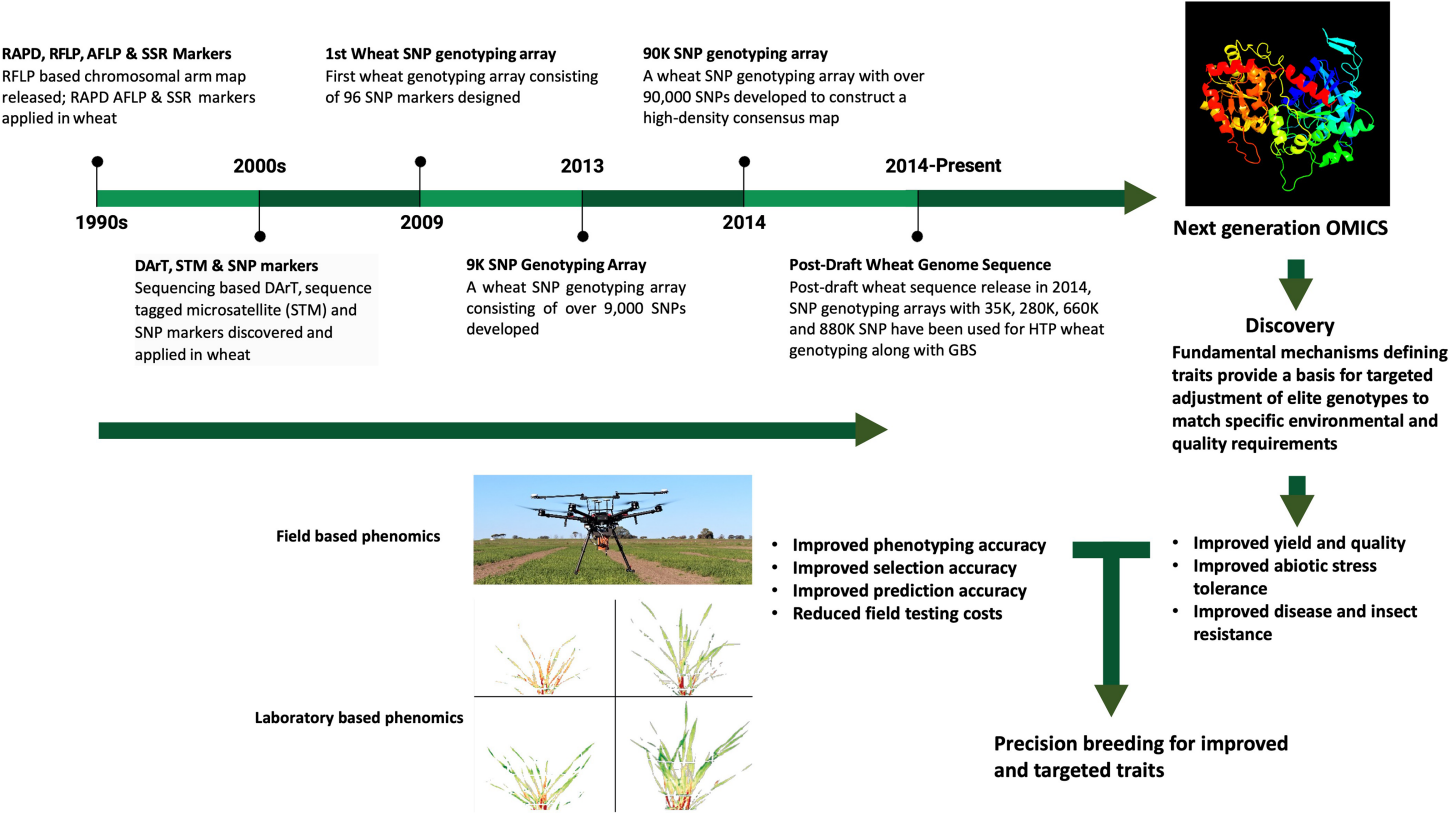
Overview of the parallel progress in the analysis of the wheat genome and high throughput phenotyping. The top panel provides the timeline for wheat-genome studies, opening up of the next generation omics; the image on the far right is the modeling of wheat granule-bound starch synthase, using Phyre 2 (Kelley et al., 2015). The lower panel emphasizes the progress of both field-based phenomics (image of drone with spectral-recording equipment, kindly provided by S. Kant, Agriculture Victoria, Grains Innovation Park, Horsham, VIC, Australia) and laboratory-based high-throughput analyses (part of Figure 6 of Banerjee et al., 2020), showing false-color composite from hyperspectral data of wheat leaves, kindly provided by S. Kant.

In summary, these genome assemblies represent an essential, highly efficient resource for wheat researchers and breeders, to identify and clone major genes and QTL, to elucidate regulatory regions, including miRNAs and transcription factors, and gene networks involved in yield, as well as biotic- and abiotic-stress tolerance in wheat, thereby facilitating their use in wheat improvement programs (Figure 1). Capturing phenotypes (as QTL) at the genomic level has brought a sharp focus on the repetitive nature of the gene-coding space in the wheat genome, allowing both copy number variation, as well as sequence variation *per se*, to be correlated with phenotype variation. The distribution of essentially identical genes on different non-syntenic chromosomes provides for further flexibility, as well as complexity, in fine-tuning phenotypes to specific environments.

In this paper, we consider the potential of current whole-genome assemblies to improve the accuracy and resolution of genetic mapping, QTL mapping, genome-wide association studies (GWAS), and the use of sequence-based markers for efficient MAS and genomic selection. A key focus for the review is the identification and cloning of major candidate genes (CG), for traditional wheat-breeding programs, and modern ones using Clustered Regularly-Interspaced Short Palindromic-Repeats (CRISPR) associated protein 9 (CRISPR/Cas9)-mediated genome editing, and bioinformatics tools for wheat improvement complement the advances in genotyping and phenotyping. They herald the start of what might be considered a golden era of wheat genomics-assisted breeding, promoting the aim of sustainably intensifying global-food production.

## Wheat Reference-Genome and *in silico* Bioinformatics

The so-called next gene generation sequencing (NGS) technologies, as well as high-resolution optical mapping generate large data sets (Dorado et al., 2019). They have driven improvements in bioinformatics tools used to deal with the challenge of analyzing such large data sets. Advancements have been made possible by a dual-strategy approach, focused on hardware and software. They have included clock-frequency increases for the Central Processing Units (CPU) of computers, node reduction, multicore (a few), many core (higher number) and integration through System on a Chip (SoC) with unified memory between the CPU and Graphics Processing Units (GPU). Machine learning, artificial intelligence, dedicated artificial neural network (ANN) analyses, and massive parallelism are enhanced using multi-core architectures (Gálvez et al., 2016, 2021). All this has contributed to our ability to sequence *de novo*, assemble, and annotate extremely large and complex genomes.

Abundant transcriptome sequence data have been generated in wheat, being freely available in open-access servers such as Wheat Expression,^4^ which can be utilized to narrow down the candidate genes identified in GWAS analyses, for further validation projects. The availability of sequenced mutant populations has opened doors to conduct validation studies.^5^ Predesigned single-nucleotide polymorphism (SNP)-based primers are available in the Ensemble database^6^ for validating mutations, which can then be combined to develop double- or triple-null mutants, for research and breeding applications. Efforts in this direction are expected if “causal” genes are to be identified. Transcriptomics and other expression analyses have generally been combined with QTL and metaQTL (mQTL), to narrow down and validate the candidate genes (Oyiga et al., 2018; Gálvez et al., 2019; Mérida-García et al., 2020). We expect it to be applied more frequently in marker-assisted and genomic selection. Continued advances in fingerprinting genome regions of interest, through improved designs of SNP arrays and associated statistical analyses of imputation, is establishing haplotype analyses as a more appropriate method to represent QTL, rather than relying on single-candidate genes.

**Figure 2:**
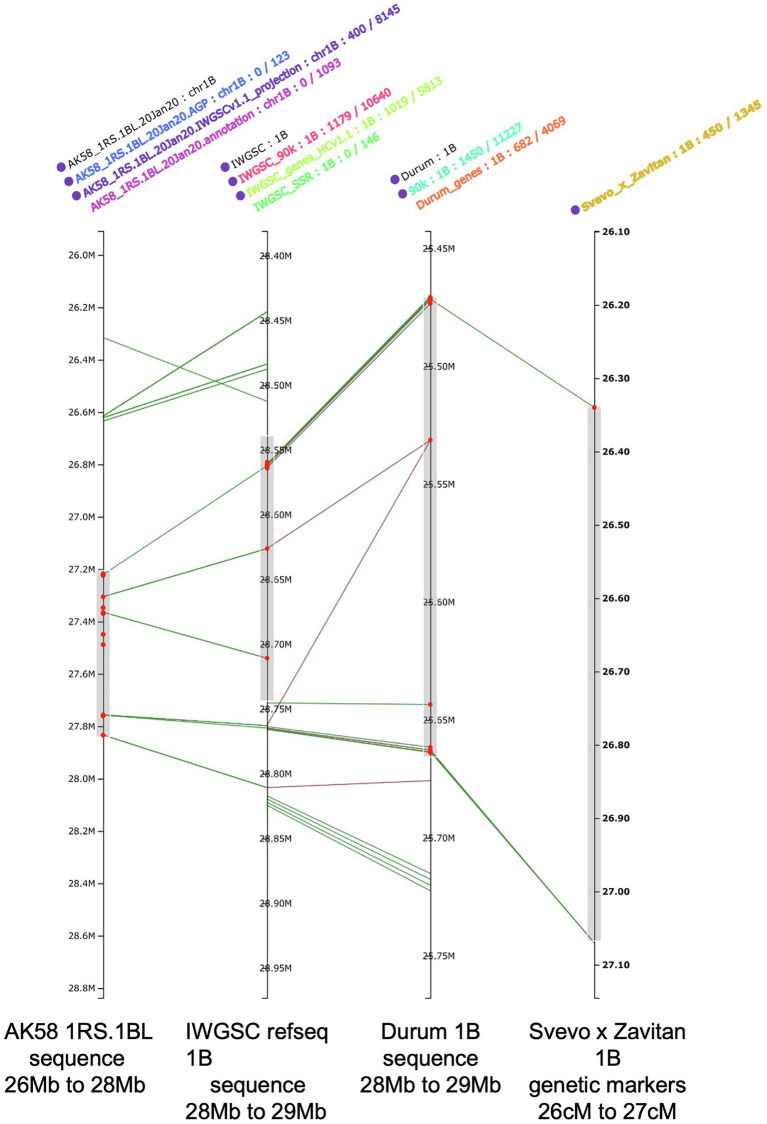
Aligning genome maps for SNP, DArTseq and/or GBS markers with IWGSC RefSeq 1.0 using PRETZEL https://plantinformatics.io. The right most map provides the location of a QTL for the emergence of additional seminal roots (midpoint = 26.6 cM; Golan et al., 2018) from a Svevo x Zavitan map based on a 90K SNP chip. The second map (from the right) is the durum genome sequence for 1B, available at the URGI with the SNPs annotations included. The second map from the left is the IWGSC RefSeq 1.0 with the HC ver1.1 gene annotation, the 90k SNP annotation, the LC ver1.1 gene annotations and the SSR annotations included. The left most map is the genome sequence for the 1RS.1BL sequence from wheat cv Aikan58 (Ru et al., 2020) with three sources of gene annotations included. The red dots identify the gene models predicted to be located in the QTL identified in the Svevo x Zavitan QTL.

A significant challenge that scientists have faced with the availability of the wheat genome reference (IWGSC RefSeq v 1.0) is its integration with the previously published genetic maps harboring QTL for various traits. Large datasets including physical maps, sequence variations, gene expression, markers, and phenomic data have already been integrated on the IWGSC RefSeq v1.0 (Wheat@URGI portal; Alaux et al., 2018). Furthermore, haplotype-based integration of different marker types and the capacity to align early genetic maps with the reference genome are refined, capitalizing on existing information of trait-linked SNP, DArTseq and/or Genotype By Sequencing (GBS) markers (PRETZEL; Figure 2).^7^

## Characterization of Genes and Gene Families Using the Wheat Reference-Genome

As indicated above, the availability of reference genome sequences in many crop species, including wheat, has sparked the publication of many works about genomics and breeding of such species, using bioinformatics tools, with special emphasis on previously unknown areas of the genome. For instance, *in silico* analyses of the published wheat reference genome, IWGSC RefSeq v1 (Appels et al., 2018), have allowed the identification and characterization of the gene families. They include: (i) Domain of Unknown Function (DUF-966, *TaDUF966*) gene family, involved in salinity-stress tolerance (Zhou et al., 2020); (ii) MADS-Box gene (*TaMADS*-box) family members, involved in wheat growth, development, and abiotic stresses (Raza et al., 2021); (iii) Gretchen Hagen3 (*TaGH3*) gene family, important in various biological processes, including phytohormone responses, growth, development, metabolism, defense, and abiotic-stress tolerance, such as salinity and osmotic ones, with polyploidization contributing to their high number (Jiang et al., 2020); (iv) superoxide dismutase (SOD) gene (*TaSOD*) family, encoding antioxidant enzymes scavenging reactive oxygen species (ROS), also involved in plant growth, development, and abiotic-stress tolerance, including drought and salinity (Jiang et al., 2019); (v) non-specific lipid transfer proteins (nsLTP/LTP) gene (*TansLTP/TaLTP*) family, involved in transporting phospholipids across membranes, growth, development, and abiotic stresses, such as drought and salinity, showing high numbers, due to gene duplications (Fang et al., 2020a); (vi) REMorin (REM) gene (*TaREM*) family, involved in vernalization, plant-microbe interactions, hormonal regulation, development, and tolerance to biotic and abiotic stresses, including cold acclimation (Badawi et al., 2019); (vii) S-phase Kinase-associated Protein 1 (SKP1) gene (*TaSKP1*) family, encoding core subunits of the Ubiquitin Proteasome 26S (UPS) and have expanded through duplications, being involved in development and stress signaling (El Beji et al., 2019); (viii) subtilase or subtilisin-like protease (SBT) genes (*TaSBT*), involved in many biological functions, such as defense and tolerance to biotic stresses caused by pathogens, among which are *Puccinia striiformis f*. sp. *tritici*, which is the fungus generating the wheat stripe-rust disease (Yang et al., 2020); (ix) highly and structurally conserved “Soluble N-ethylmaleimide Sensitive Factor (NSF; SNF) Attachment Protein (SNAP) REceptor” (SNARE) and Novel Plant SNare (NPSN) gene families (*TaSNARE* and *TaNPSN*, respectively), involved in growth and development, regulating vesicle trafficking, fusion, and targeting to vacuoles and exocytosis (Gaggar et al., 2020); (x) “DNA binding with one finger” (Dof) gene (*TaDof*) family, encoding zinc-finger transcription factors (*TaDof*), involved in phytohormone response, growth, development, metabolism, defense, and stress responses, including both abiotic (such as salinity and drought) and biotic ones, with their high numbers due to polyploidization, showing many segmental duplications and both miRNA and cis-regulators involvement in modulating their gene expression profiles (Fang et al., 2020b); and (xi) basic leucine ZIPper (bZIP) gene (*TabZIP*) family, encoding transcription factor, being involved in plant growth, development, metabolism, chlorophyll content, photosynthesis, membrane stability, and tolerance to stresses, including abiotic ones such as drought, salinity, and heat, also involving oxidative stress (Agarwal et al., 2019). These gene families complement many gene models identified by highly conserved domains, using model organisms such as rice and *Arabidopsis*, and provide a matrix across the wheat genome, to associate with phenotypic variation in order to establish a new matrix of functionally, with annotated gene models predicted to affect wheat phenotype.

Bioinformatics analyses of the published wheat reference genome, IWGSC RefSeq v1.0, have also enabled the comparisons of cDNA, identification, and annotation of genes. These comparisons revealed different metabolic pathways, including starch and sucrose, as well as genes related to abiotic- and biotic-stress tolerance, signaling and transportation (Kaur et al., 2019). Delivering the range of bioinformatics outputs from the wheat genome sequence has required innovation in the production of genotyping microarrays, such as the Axiom Wheat high-density Genotyping Array, Axiom Wheat Breeder’s Genotyping Array, and GeneChip Wheat Genome Array from Affymetrix-Applied Biosystems-Thermo Fisher Scientific.^8^ The efficacy of the SNP arrays can now be evaluated using a three-way classification system, permitting sorting of SNP into three quality groups (Lange et al., 2020).

The concept of having the wheat genome on a chip is currently under development by the Arbor Biosciences IWGSC exome array group, working with the IWGSC, with a first release in 2019 of the Baits Expert Wheat Exome capture panel, based on the complete high-confidence exon-annotated wheat genome. It included two million probes, targeting more than 200 megabases of high-confidence exons.^9^ An extra level of fine-tuning for gene regulation of different biological processes, including metabolism, growth, development, transport, cell signaling, structural proteins, and abiotic- and biotic-stress tolerance, is afforded by the characterization of new microRNA (miRNA), including polycistronic miRNA, in cultivated and wild species. The genes targeted by these small RNA can be predicted, and some are monomorphic, whereas others are polymorphic, all represented on the Expert Exome capture chip (Singh et al., 2020).

The small RNAs are spatially and temporally regulated, being involved in post-transcriptional gene regulation, including transcription factors, and thus complement the genome-wide transcriptomics approach established to dissect the dynamics and underlying regulation of key processes, such as wheat-spike development. Genes involved are related to meristem maintenance, initiation and transition, development of flowers, and flowering response to stress (Li et al., 2018b).

### Grain Yield and Related Traits

Wheat grain yield is controlled by numerous genetic components, most of which are quantitative in nature. Due to this underlying complexity, QTL mapping is commonly used for the dissection of grain yield and yield components, in order to identify markers for MAS. Prior to the 2014 draft sequence, several QTL studies have reported using redundant SSR-markers for QTL mapping of GY and related traits, as recently reviewed (30, see also Supplementary Table S1), but most of these regions were not incorporated into wheat cultivars in breeding programs using MAS. The IWGSC draft sequence published in 2014 (IWGSC, 2014) enabled the use of genotyping arrays and GBS, with deep coverage to construct high-density linkage maps and identify several candidate genes (Addison et al., 2016; Su et al., 2016; Assanga et al., 2017; Cui et al., 2017; Hussain et al., 2017b; Kuzay et al., 2019; Jin et al., 2020). Major and stable QTL for plant height, anthesis date, flag-leaf length and width, as well as spike length, density and spikelet number per spike were mapped on chromosome 2D and 4B, with individual phenotypic variation (PV) range of 10.10%–30.68%. Other QTLs were mapped on chromosomes 4A and 6D. The markers were associated with candidate genes coding for TGTCTC auxin response elements, F-box protein TIR1, flowering locus T-like protein, MADS-box transcription factor 8 and 12 genes encoding SAUR-like auxin-responsive family proteins (Jin et al., 2020). Three independent studies identified haplotype SNP markers and major stable QTL for seed number per pod (SNPP), thousand-grain weight (TGW), grain length, flag leaf length, width, and area on chromosomes 7A (Su et al., 2016; Hussain et al., 2017b; Kuzay et al., 2019) and 5A (Hussain et al., 2017b). These QTL were associated with candidate genes, such as *Wheat ortholog of Aberrant Panicle Organization 1* (*WAPO1*) and *TaGASR7*. Among these, a well-studied and reproducible yield QTL on the long arm of chromosome 7A has been located to an 87-kbp region (674,019,191–674,106,327 bp, IWGSC RefSeq v1.0), containing two full and two partial genes. The ortholog of one of these genes (*TraesCS7A01G481600*) was *APO1*, which is known to significantly affect panicle attributes (Kuzay et al., 2019). This *APO1* ortholog was the best candidate for the spikelets per spike phenotype, being associated with two amino acid changes (C47F and D384N) in the coding region. In the genomic region carrying the chromosome 7A *APO1* gene, three major haplotypes were associated with the spikelets per spike phenotype, and two of these show enrichment in modern germplasm (Kuzay et al., 2019; Voss-Fels et al., 2019). More recently, genetic analyses were carried out using a wheat multi-founder population genotyped with a 20K SNP array. They found that allelic variation at the homoeologous location on chromosome 7B was associated with haplotype variation at the *WAPO-B1* gene (Corsi et al., 2021). Another recent example of the use of high-density SNP arrays for the genetic mapping of yield components was the use of a 660K SNP array that led to the identification of a stable major QTL for grain number per spike on chromosome 4A that corresponded to 65 putative genes (Cui et al., 2017) and contributed 8.0%–21.2% to PV.

Networks linking quality attributes of the grain to the yield of the grain are evident, as genes initially identified in the quality space have been characterized for their roles in the improvement of yield and related traits. Examples of several such genes include *TaGW2* (Su et al., 2011), cell-wall invertase *TaCwi-A1* (Ma et al., 2012), *TaGASR7-A1* (Dong et al., 2014), *TaGS-D1* (Zhang et al., 2014), *IAA-glucose hydrolase* gene, *TaTGW6* (Hu et al., 2016a), and *TaTGW-7A* (Hu et al., 2016b). Marker-trait associations (MTAs) for yield and related traits have been linked to candidate genes/loci such as *Rht-B1*, *Rht-D1*, *Vrn-A1*, *Ppd-D1*, *TaSus1*, *TaSus2*, *TaGS-D1*, and *TaGW2-6B* (Lopes et al., 2015; Zanke et al., 2015). Importantly, a GBS-based GWAS for 768 wheat accessions identified 395 QTL for plant height, DH, SNPP, spike length and number, grain length, grain width, and TGW under seven environments (Pang et al., 2020). These QTL were closely linked with several candidate genes including, but not limited to, yield-related genes: *APO1*, *AUX1*, *Ehd2*, *GSN1*, *GL3*, *Gn1a*, *MADS14*, *MADS15*, *MADS18*, *MADS57*, *Rht-D1*, *Rht12*, *TaGA2ox8*, *TaSus1-7B*, *Vrn-B1*, and *Vrn-D1*.

Within the matrix of the functional wheat genome, projection of yield-related QTL onto a set of well-defined wheat genes (99,386) identified 32 metaQT including 18 grain-yield mQTL associated with 15,772 genes (28,630 SNP), 37 of which were major candidate genes (Quraishi et al., 2017), including *ATPase*, *GIF1*, *Ppd-D1*, *Prog1*, *Gn1-a*, *NYC1*, *emp4*, *DEP1*, *GW2*, *GS2*, and *Rc3* (Pang et al., 2020).

### Drought Tolerance

In candidate gene-based association mapping approaches, resequencing of genes with known or predicted biochemical function is performed, and SNP variation identified within a candidate gene is used to investigate associations with traits (Iehisa et al., 2014; Supplementary Table S2). Using this approach, allelic variation in four drought-related genes has been investigated in wheat. Edae and colleagues (Shokat et al., 2020) reported associations of SNP in three genes, *DREB1A*, *ERA1*, and *1-FEH*, with multiple agronomic and physiological traits. These are known to be drought stress-induced genes in ABA-dependent (*ERA1*) and ABA-independent (*DREB1A*, *1-FEH*) pathways. In another CG-based association mapping study, allelic variation in *TaSnRK2.8* (a SNF-1 type serine–threonine protein kinase) showed association with plant height, flag leaf width, and water-soluble carbohydrates, under drought conditions (Zhang et al., 2013). Other CG involved in wheat AB/ABA-dependent/ABA-independent signaling pathways have been described, including *DREB1*, *WRKY1*, *DREB1A*, *HKT-1*, *DREB2*, *DREB3*, *ERA1-B*, *ERA1-D*, *1-FEH-A*, and *1-FEH-B* (Budak et al., 2015). Finally, a drought tolerance QTL on chromosome 6D was associated with regulation of expression of late embryogenesis abundant (LEA) genes such as *TaABA8*, *OH1*, *CYCB2*, and *CDKA1* (Iehisa et al., 2014), suggesting that these genes play a role in drought tolerance. Several clusters of drought-responsive genes (*DREG*) have been found on the long arm of the group 5 chromosomes, with orthology to a known QTL of rice (*Oryza sativa*). In particular, this region contains the genes *PSY3*, *NCED*, *VRN1*, *UGDH*, and the dehydrin *DHN38* that increase their expression under field drought stress conditions (Gálvez et al., 2019). Similarly, transcriptomics analyses of high yielding and drought tolerant United States wheat-cultivars TAM 111 and TAM 112 identified several DREG. For instance, under drought stress, aquaporin, dehydrogenase, kinase, phosphatase synthase, phosphorylase, and sugar transporter were downregulated. On the other hand, dehydrin, ABA-inducible protein kinases, LEA protein, heat-shock protein, caleosin, lyase, amylase, and oxidoreductase were upregulated in such environments (Chu et al., 2021). These studies now clearly engage the very broad aspects of wheat biology, providing a subset of genes for more detailed study in characterizing variation in drought tolerance, facilitating the identification of suitable parents for breeding.

Large-scale haplotypes-based GWAS studies, combined with epistatic interactions, have been undertaken to untangle the genetic architecture of grain yield under multiple stress environments (including mild and severe drought stress), using a large panel of 6,333 advanced lines, from the International Maize and Wheat Improvement Centre (CIMMYT; Sehgal et al., 2020a). This study reported haplotype associations with grain yield, under mild (four datasets) and severe (10 datasets) drought stress environments. Most importantly, the authors identified a significant association of a haplotype block close to the *Vrn-B1* flowering time gene on chromosome 5B, with GY in more than 70% of the trials under severe drought stress. *Vrn-B1* is significantly correlated with adaptation to low temperature, thus indicating a shared tolerance mechanism for both abiotic stresses.

### Heat Tolerance

A candidate gene approach to heat tolerance allowed the examination of variation in Fv/Fm, a parameter for assaying the maximum quantum efficiency potential of Photosystem II (Supplementary Table S3). It was linked to three major QTL mapped on chromosomes 3B and 1D (Sharma et al., 2017), involving two light-reaction genes, chloroplastic NAD(P)H-quinone oxidoreductase subunit 2B (*ndhB2*) and photosystem I iron–sulfur center (*psaC*). Two other genes, known to control chloroplastic 3-isopropylmalate dehydrogenase 2 (*IMDH2*), were also suggested to be involved in metal binding during photosynthesis. Other candidate genes, such as beta-glucosidase 26 (*βglu26*) and fructokinase 2 (*frk2*; Sharma et al., 2017), were involved in carbohydrate metabolism. Response to thylakoid membrane damage, plasma-membrane damage and SPAD chlorophyll-content QTL were mapped on chromosomes 1B, 1D, 6A, and 7A. Cell membrane stability was on chromosomes 1D, 2B and 7A (Talukder et al., 2014). These QTL were associated with candidate genes such as *srg6*, calcium/calmodulin-dependent kinase (*CDPK*), topoisomerase I (*Top1*), and aquaporins. A GWAS study identified 20 significant MTA for cell membrane stability on chromosomes 1A, 1B, 2A, 4A, 4B, 6B and 7B, 13 of which were situated solely on 6B (ElBasyoni et al., 2017). All these SNP were then annotated as candidate genes, glycerophosphoric-diester phosphodiesterase (*GDE1*), TRAF-type zinc-finger protein, *SWI3B*, and *ATPase* (Supplementary Table S3). The network nature of grain yield, heat, and drought susceptibility indices, as well as yield stability coefficient, were also reported (Sehgal et al., 2017), as being associated with the flowering time genes *Vrn-B1*, *Ppd-D1*, and *Vrn-D3*.

### Salinity Tolerance

Salt tolerance is a complex trait (Hussain et al., 2015). In recent years, SNP-based genotyping platforms, including 9K (Asif et al., 2018), 35K (Hussain et al., 2017a; Chaurasia et al., 2020), 90K (Oyiga et al., 2018) and 660K (Yu et al., 2020; Hu et al., 2021) arrays, have been used to identify novel and major QTL, MTA, and CG that can be used in MAS and genomic selection for salinity tolerance (Supplementary Table S4). Wheat F2 lines (WTSD91 × WN-64) were genotyped using an Axiom 35K SNP array, to develop a high-resolution linkage map, and 49 QTL for sodium ion (NAX) and potassium ion (KC) status under salinity stress were mapped (Hussain et al., 2017a). Two NAX QTL on chromosome 2A coincided with a reported major *HKT1* (*Nax1/HKT1*) QTL (Genc et al., 2010), and two NAX QTL on chromosome 7A which contributed 11.2% and 18.8%, respectively, to phenotypic variation. QTL for KC were located on chromosomes 2A, 3D, 4B, and 6A, whereas a novel Zn QTL on chromosome 7A controlled 11.2% of variation for salt tolerance. The most important Ca^2+^ (chromosome 6B) and Mg^2+^ (2A) QTL contributed 11.9% and 8.4%, respectively, to salt tolerance. Furthermore, QTL for Cu, Mn, B, P, S, and Fe were mapped for the first time (Hussain et al., 2017a). Based on SNP and expression analyses, *Nax1/HKT1*, K^+^ outward-rectifying channel (SKOR), potassium transporter 12 (*KUP12*), chloride channel protein (*CLC-e*), transparent testa 12, glutathione S-transferase U6 (*GSTU6*), peroxidase 12, auxin transport (*BIG*), auxin-response factor 5-like, *ARF21*, *NAC78*, Mg transporter (*NIPA4*), and Zn transporter 6 were identified as candidate genes (Hussain et al., 2017a). In another study, QTL for shoot growth, NAX and KC were mapped on chromosome 2B, 5A, 6A, and 7A in a wheat DH population. QTL were associated with several candidate genes. They included salt overly sensitive 1 (*SOS1*) or sodium/hydrogen exchanger 7 (*NHX7*), potassium transporter 1 (*KUP1*), *HKT2*, pyrophosphate-energized proton pump (H^+^ pyrophosphatase), *KUP12*, *SKOR* and two-pore potassium (*TPK*) channel and proton pump H^+^-ATPase 4 (Asif et al., 2018). Importantly, both of these studies (Hussain et al., 2017a; Asif et al., 2018) identified major QTL for KC and NAX on chromosomes 6A and 7A, respectively.

GWAS has been used to identify novel MTA and candidate genes for salinity tolerance (Oyiga et al., 2018; Chaurasia et al., 2020; Yu et al., 2020; Hu et al., 2021). The genotyping of 150 wheat accessions using the 90K SNP array, and a subsequent GWAS, identified 187 SNP and 37 QTL for leaf NAX and KC, including four QTL on chromosomes 2A, 3A and the short arm of chromosome 1B, in addition to novel QTL on the short arm of chromosome 1B and the long arm of 1D (Oyiga et al., 2018). Transcriptomics analyses revealed missense mutations responsible for salt tolerance variations in candidate genes, including *ZIP7*, *SAP8*, *HAK18*, *GST1*, *SWEET17*, and *KeFC*. Chaurasia et al. (2020) reported 42 MTA for shoot fresh/dry weight, chlorophyll content, seedling biomass, K^+^ and Na^+^ concentration that contributed 2.4%–42.8% to phenotype variation. These genomic regions were associated with 58 candidate genes, including transparent testa 12, chloroplast iron-superoxide dismutase, serine/threonine protein kinase (*Nek6*), ethylene responsive transcription factor (*ERF3*), *bHLH30*, and GDP-mannose transporter (*GONST1*).

Furthermore, haplotype analyses have also been coupled with GWAS to identify allelic variation for salt tolerance in wheat (Yu et al., 2020; Hu et al., 2021). A significant number of MTA (102 of 117) for germination and salt tolerance indices were found on chromosomes 1A, 3B, and 6B. They were associated with 53 candidate genes, including abscisic acid-insensitive 5-like (*TaABI5-like*), DUF674 family protein, SUMO-activating enzyme subunit 1A, glutamate formiminotransferase 1, protein KRI1, and NADH-cytochrome b5 reductase. Haplotype variations and expression of candidate genes under salinity have also been validated (Yu et al., 2020). Another GWAS analysis for yield and related traits under salinity found genomic regions and haplotypes for adult-stage salt tolerance on chromosomes 1B, 3B, 4A, 4D, 5A, 5B, an 7A. The markers were linked to several candidate genes, serine/threonine protein kinases, ricin B-like lectin gene, phytochelatin synthase, MADA-box genes, glycerol-3-phosphate acyltransferase (*GPAT*), U-box E3 ubiquitin ligase, and lipid-transfer proteins (*TaLTP*s) [73]. Additionally, 19 QTL for Mg2^+^ and Ca2^+^ were mapped at the same location as of Cl-QTL (Genc et al., 2014), with potential candidate genes chloride channel (*CLC*) and cation chloride co-transporter (*CCC*).

### Frost Tolerance

An important limiting factor for wheat production in North America, North and Eastern Europe, and Russia is low temperature (Babben et al., 2018). As polar regions become more unstable due to climate change, the risk of extreme weather events including freezing temperatures increases (Francis and Skific, 2015). Therefore, resilience to frost is an important crop trait to consider. Frost tolerance is a complex biological process, involving pathways encompassing a large number of genes. The main pathway is frost response and a prolonged period of low temperature (vernalization), which can be regarded as an avoidance mechanism to prevent frost damage to sensitive reproductive organs. Two major frost tolerance loci, Frost Resistance 1 (*FR1*) and *FR2*, were identified on the long arm of chromosome 5A (Vágújfalvi et al., 2003). Zhao et al. (2013a) described an additional frost tolerance QTL on chromosome 5B in wheat germplasm from central Europe. During the last decade, several QTL associated with frost tolerance were identified on different wheat chromosomes (i.e., 1A, 1D, 2A, 2B, 3A, 5A, 5B, 6A, 6B, 6D, and 7B; Case et al., 2014; Kruse et al., 2017). The majority of genes assumed to be involved in frost tolerance have been identified on chromosome 5 (Vágújfalvi et al., 2003; Zhao et al., 2013a; Würschum et al., 2017; Babben et al., 2018; see also Supplementary S5). Until recently, only a few studies reported the identification of QTL regions associated with frost tolerance by GWAS (Babben et al., 2018; Kandel et al., 2018). Babben et al. (2018) demonstrated the utilization of the IWGSC RefSeq v1.0 in the specific primer development for highly conserved gene families in wheat. It showed that a candidate-gene association genetics approach is a useful tool for identifying new alleles of genes important for response to flowering time. Sequence analyses concluded that C-repeat binding factors (CBF)-A3, 5, 10, 13, 14, 15, and 18, vernalization response genes (*VRN-A1*, *VRN-B3*) and photoperiod response genes (*PPD-B1* and *PPD-D1*) were associated with frost tolerance in wheat. In addition to winter hardiness as described above, an additional and critical frost-resistance phenotype relates to damage caused by transient frosts that generally occur on early spring mornings. While this trait is more complex to study, the above genes described for winter hardiness would be expected to contribute to this tolerance as moderators.

During the last decade, several components encompassing messenger molecules, protein kinases, and phosphatases, as well as transcription factors, which are involved in cold-stress signaling pathways, have been reported by studies using wheat sequence information (Wei and Han, 2017; Babben et al., 2018; Jin et al., 2018; Guo et al., 2019). The CBF (C-repeat binding factor), Inducer of CBF Expression (*ICE*) and cold-responsive (*COR*) genes or *ICE-CBF-COR* are part of the main cold-signaling pathway, playing a major role in controlling frost tolerance for crop species (Jin et al., 2018; Guo et al., 2019). *ICE* genes belong to the *MYC* family transcription factor and *MYC* subfamily of *bHLH* (Basic Helix–Loop–Helix; Guo et al., 2019). ICE factors are known as positive CBF expression regulators, considered to act upstream of the low-temperature signaling pathway. Two ICE homologs such as *TaICE41* and *TaICE87* have been identified in wheat (Guo et al., 2019). The *TaICE41*, *TaICE87*, and five MYC-like *bHLH*s were positively regulated upstream of the CBF mediated transcriptional cascade, controlling cold tolerance in wheat (Wei and Han, 2017).

### Cloning of Multiple Disease and Insect-Resistance Genes

Examples of the positional or map-based cloning of disease resistance-related genes in wheat are arguably more common than for non-disease resistance traits—presumably due to the gene-for-gene interaction of such major resistance genes with specific avirulent factors in the pathogen, or other major effects (Supplementary S6). These include genes such as *Lr21* (Huang et al., 2003), *Yr36* (Fu et al., 2009), *Yr15* (Klymiuk et al., 2018), *YrU1* (Wang et al., 2020b), *Fhb1* (Su et al., 2019), *Fhb7* (Wang et al., 2020a), *SuSr-D1* (Hiebert et al., 2020), *Yr7*, and *Yr5/YrSP* (Marchal et al., 2018). They have been cloned in wheat, to improve resistance against leaf and yellow rusts, in addition to fusarium head-blight diseases (Supplementary Table S5). Additionally, various genes were cloned for resistance against stem rust (*Sr22*, *Sr33*, *Sr35*, *Sr45*, *Sr46*, *Sr60*; Periyannan et al., 2013; Saintenac et al., 2013; Steuernagel et al., 2016; Arora et al., 2019; Chen et al., 2020), necrotrophic blotches (*Tsn1*, *Snn1*, *Stb6*, *Stb6q*; Faris et al., 2010; Shi et al., 2016; Saintenac et al., 2018, 2021), powdery mildew (*Pm1a*, *Pm2*, *Pm3*, *Pm3b*, *Pm5e*, *Pm21*, *Pm24*, *Pm38/Lr34/Yr18/Sr57*, *Pm41*, *Pm46/Lr67/Yr46/Sr55*, *Pm60*; Yahiaoui et al., 2004; Krattinger et al., 2009; Hurni et al., 2013; Moore et al., 2015; Sánchez-Martín et al., 2016; Xing et al., 2018; Zou et al., 2018; Li et al., 2020; Lu et al., 2020; Xie et al., 2020), being detailed in Supplementary Table S5. Access to a high-quality genome reference of wheat (IWGSC RefSeq v1.0) has also enabled researchers to explore susceptibility factors that may contribute to the onset of disease. For instance, Henningsen et al. (2021) explored the upregulation of wheat genes with orthology to known susceptibility factors in other plant species, in response to stem-rust fungus. They hypothesized genes that may play a conserved role in susceptibility. Similarly, a study by Corredor-Moreno et al. (2021) combined gene expression analyses and the genome reference of wheat (IWGSC RefSeq v1.0) and provided the basis for showing that the branched-chain amino acid aminotransferase gene (*TaBCAT1*) contributed to susceptibility to both stripe and stem rust. The authors from both studies suggested that manipulation of susceptibility genes can result in novel strategies to control disease. Fine-mapping and leveraging of available wheat pan-genome datasets, together with TILLING resources, have been utilized to analyze these complex situations. For example, a possible gene driving the complex interactions underlying *Sm1*-mediated resistance to orange wheat-blossom midge (OWBM, *Sitodiplosis mosellana* Géhin) wheat insect pest has been identified as a canonical NLR, with kinase and major sperm protein integrated domains (Walkowiak et al., 2020).

GBS-based GWAS identified 27 MTA for powdery mildew (7 MTAs), stem rust (5 MTAs), septoria (3 MTAs) and leaf rust (12 MTAs) resistance on all chromosomes (except for 4B and 5D; Bhatta et al., 2019). MTA were associated with several candidate genes for leaf rust; namely *GDSL* esterase/lipase, vesicle-associated 1-1-like protein, E3 ubiquitin ligase family protein, phosphatidic acid phosphatase, 12-oxophytodienoate reductase-like protein, septoria (F-box/RNI-like/FBD-like domains-containing protein) and stem rust (zinc transporter, putative). Leaf and stem-rust candidate genes associated with MTA were members of the *NLR* (nucleotide-binding domain leucine-rich repeat) gene family, nuclear monodehydroascorbate reductase 6 (*MDAR6*), solanesyl-diphosphate synthase 1 (*DSDS1*), enhancer of AG-4 protein 2 (*AG4*), phosphatase 2C (*PP2C*), and importin-9 (*IPT9*), being listed in Supplementary Table S5.

Advances in wheat genomics have facilitated the cloning of nine stripe-rust resistance genes (*Yr5*, *Yr7*, *YrSP*, *Yr15*, *Yr18/Lr34*, *Yr36*, *Yr46*, *YrAS2388*, and *YrU1*), out of the >80 genes that have been identified and mapped so far in different genetic backgrounds of wheat (Wang et al., 2020b). Cloning of the broad-spectrum stripe-rust resistance (R)-gene (*Yr15*), derived from wild emmer wheat, has led to the discovery of a novel protein family, the tandem kinase-pseudokinases (*TKP*) that emerged as a new class of disease-resistance protein family (TKP), providing plant innate immunity, being present not only in wheat, but also across the whole plant kingdom (Klymiuk et al., 2018). Five plant-disease resistance genes have been identified so far to contain a structure with tandem kinase domains, including three wheat genes, i.e., the wheat-stripe-rust R-gene WTK1 (*Yr15*; Klymiuk et al., 2018, 2020), wheat-stem-rust R-gene WTK2 (*Sr60*; Chen et al., 2020), and wheat powdery-mildew [*Blumeria graminis* f. sp. *tritici* (Bgt)] R-gene WTK3 (*Pm24*; Lu et al., 2020). More than 20 WTK copies have been found to be scattered across the three wheat genomes, AA, BB, and DD, including the orthologous group in chr 1 and the paralogous groups on chromosome 6 (Klymiuk et al., 2019). WTK1 orthologs, paralogs, and homologs were found also in the diploid wheat relatives, *Triticum urartu* (AA), *Aegilops speltoides* (SS), and *A. tauschii* (DD), representing the ancestral A, B, and D genomes, respectively, as well as in rye (*Secale cereale*), barley (*Hordeum vulgare*), and other cereal species. The protein sequences of TKP were obtained from genome assemblies of wild and cultivated wheat species, being used for phylogenetic analyses (Klymiuk et al., 2018), because it is important for successful deployment of R-genes in wheat breeding programs to identify if a cloned gene differed from other genes localized in the same chromosome region, or may represent different alleles of the same gene. For example, it was found that *Yr15*-, *YrG303*-, and *YrH52*-mediated resistances to yellow rust are encoded by a WTK1 as a single locus (Klymiuk et al., 2020). In future, we expect that many more such cases will be revealed, narrowing down the list of designated R-genes in wheat.

Ten powdery mildew (*Pm*) genes have been cloned so far, alongside with advances in wheat genomics resources (Supplementary Table S5). *Pm3/Pm8*, *Pm2*, *Pm21*, *Pm60*, *Pm5e*, *Pm41*, and *Pm1*a encode NLR-immune receptors from different wheat relatives (Yahiaoui et al., 2004; Hurni et al., 2013; Sánchez-Martín et al., 2016; Xing et al., 2018; Zou et al., 2018; Li et al., 2020; Xie et al., 2020), while a tandem kinase protein is encoded by *Pm24* (Lu et al., 2020). Furthermore, two non-NLR genes—*Pm38* and *Pm46*, showed broad-spectrum multi-adult plant resistance to powdery mildew and rust diseases. An ABC transporter is encoded by the *Lr34/Yr18/Sr57/Pm38* multi-resistance gene (Krattinger et al., 2009), and a hexose transporter is encoded by the *Lr67/Yr46/Sr55/Pm46* multi-resistance gene (Moore et al., 2015). The cloning of these *Pm* genes enables the development of high-throughput diagnostic functional markers that can be used in MAS for fungi-resistance breeding programs (Supplementary Table S5). Some of these *Pm* genes have been widely used for the protection of wheat cultivars for many years. For example, Triticeae grass *Dasypyrum villosum* (2n = 2x = 14, VV) harbors *Pm21*, which confers broad-spectrum resistance, and was transferred in China into wheat cultivars (T6AL.6VS wheat-*D. villosum* translocation line) in 1995 (Chen et al., 2013). Some of these NLR proteins could be overcome by the fast evolution of virulent *Blumeria graminis* (Bgt) isolates, especially when the gene is widely deployed in wheat fields. For example, wheat-rye 1BL·1RS translocation carrying *Pm8* has lost the resistance function for wheat variety production (Zeng et al., 2014). Different alleles of those cloned *Pm* genes that might be resistant to different *Bgt* isolates have been identified and could also be used for MAS. For example, 17 alleles of the *Pm3* gene have been identified mediating resistance to distinct race spectra of Bgt. *Pm3a* has a range of resistance that fully encompasses that of *Pm3f*, but also extends to additional races (Brunner et al., 2010). Therefore, enriching the *Pm* gene pools is very important for resistance breeding. *Pm24* is a rare natural allele of tandem kinase protein (TKP), with putative kinase-pseudokinase domains, conferring broad-spectrum resistance to wheat powdery-mildew disease. However, there are some other *Pm* genes that have not been cloned yet, such as *Pm30*, found in ~80% of Chinese cultivars, as detected by closely linked markers (Cheng et al., 2020). The absence of functional molecular markers is limiting the diagnosis of potential *Pm* alleles, and their deployment in wheat breeding, *via* MAS and genome editing.

Two independent GWAS analyses utilizing iSelect 9K and 90K Illumina arrays have reported SNP and genes for Soil-Borne Wheat Mosaic Virus (SBWMV) resistance (Liu et al., 2014, 2020). Liu et al. (2020) completed a GWAS analysis of SBWMV resistance using the 90K Illumina array. Thirty-five SNP in 12 wheat genes and one intergenic SNP in the Sbwm1 region were identified on chromosome 5D, being associated significantly with SBWMV resistance. Resistance to SBWMV was strongly associated with a predicted kinase family protein (Liu et al., 2014). Furthermore, GWAS analyses identified major resistance SNP for Wheat Spindle Streak Mosaic Virus (WSSMV) on chromosome 2D, in addition to regions on 5B and 7D. The 2D genomic region was linked with 18 candidate genes, including 11 NBS-LRR ones (Hourcade et al., 2019), being listed in Supplementary Table S6.

Insect resistance (Supplementary Table S7) has been explored, utilizing the wheat stem sawfly (WSS) transcriptome and its interaction with the regulatory elements, microRNA<--abbreviation indicated above-- and long non-coding RNA (lncRNA). Interestingly, the study found that WSS miRNA may target wheat transcripts and vice versa, thereby potentially modulating plant responses against WSS (Cagirici et al., 2017). The solid-stem trait, associated with WSS resistance, was linked to copy-number variation of a putative Dof Transcription Factor (TdDof) within the 3BL QTL, through the use of high-throughput sequencing in different genetic backgrounds. Transgenic lines over-expressing TdDof firmly established that increased expression of TdDof was responsible for solid stemness, likely through regulation of programmed cell death in pith parenchyma cells (Nilsen et al., 2020). Similarly, genome sequencing in resistant and susceptible cultivars revealed a candidate gene in the Sm1 locus that is known to confer resistance to orange wheat blossom midge (OWBM). This time, knockout mutant lines demonstrated that mutations within this gene resulted in susceptibility against OWBM. The candidate gene contains NB-ARC, and LRR motifs, in addition to a serine/threonine (S/T) kinase that is similar to those found in rust resistance proteins, and a major sperm protein (MSP) domain (Walkowiak et al., 2020).

### End-Use Quality Traits

Wheat grain markets and food industries demand not only high yielding and resistant varieties, but also those with specific end-use qualities. End-use quality is, therefore, an important focus in breeding programs. Methods, for testing quality, however, require large amounts of grain and are time-consuming and costly. Significant efforts have been made to identify QTL linked to various end-use quality traits such as grain protein content (GPC), dough rheological properties, and baking quality (Supplementary Table S7). Several comprehensive analyses (Sun et al., 2008; Raman et al., 2009; Carter et al., 2012; Simons et al., 2012) of mapping several quality traits related to protein and starch have been conducted. Sun et al. (2008) analyzed GPC, flour protein content (FPC), grain glutenin macropolymer content, wet gluten content (WGC), dry gluten content (DGC), Zeleny sedimentation volume, flour–water absorption (FWA), dough development time (DDT), and mixing tolerance index and flour paste viscosity (Supplementary Table S7). They identified 30 QTL for starch traits and 15 QTL for protein traits, with QTL clusters for starch traits located on chromosomes 3D, 6B, and 7B, and protein traits on 1D and 3B. Raman et al. (2009) analyzed GPC, milling yield, FPC, flour color, FWA, DDT, dough strength (DS), and dough extensibility (DE). They found several QTL associated with DS, DE, DDT, and FWA, close to glutenin (*Glu-B1*) locus on chromosome 1B. Simons et al. (2012) analyzed 20 end-use quality traits including six grain, seven milling and flour, four dough mixing strength, and three bread-making traits. They found that the 1DL QTL cluster containing *Glu-D1* had a large genetic influence on dough mixing strength and bread-making performance. Furthermore, two QTL clusters located on chromosomes 3B and 4D associated with several milling and baking quality traits (Carter et al., 2012) were reported, being associated with the *Wx-B1*, *Glu-B1* and *Glu-D1* genes.

Specific attributes of starch contribute unique properties to certain wheat-breeding lines and the genome level characterization of one such property, udon noodle quality, is detailed in (Appels et al., 2018). The Granule Bound Starch Synthase (*GBSS; TraesCS4A01G418200*) gene on chromosome 4A is absent from some lines, being the null allele for *GBSS*-4AL (*Wx-B1b*) that associates with udon noodle quality. Significant sections of *TraesCS4A01G418200* were absent from the exome sequences of 3.9% lines of a set of 644 (hexaploid) wheat varieties and landraces, assessed using 10 SNP identified from snapshot exome sequence data (Appels et al., 2018). The specific deletions within the *GBSS*-4AL gene mean that the respective lines provide new germplasm sources for wheat breeding. The gene-specific deletions would not be expected to show detrimental effects, due to the deletion of adjoining gene models, and thus be expected to perform successfully at the agronomic level, to satisfy the high-value commercial udon-noodle market. Other attributes of starch, such as a high amylose content for an improved source of fiber in the diet, can now be introduced into commercial wheat lines.

Importantly, a metaQTL analysis (Quraishi et al., 2017) identified stable QTL, by combining 27 quantitative genetic studies with four genetic maps. It located 73 and 82 QTL for baking quality and GPC-related traits, respectively, on a consensus map. They reported 8 metaQTL for baking quality and 6 for GPC. The most precise metaQTL having the smallest confidence intervals were located on chromosomes 3D (3.78 cM) for baking quality and chromosome 2B (5.83 cM) for GPC. The candidate genes identified are listed in Supplementary Table S7.

Recently, high-density SNP arrays and GBS have also been utilized to identify QTL for bread-making quality, using biparental populations (Jin et al., 2016; Boehm et al., 2017; Guo et al., 2020b). These high-resolution genetic maps helped to precisely identify major QTL and candidate genes, thus providing a valuable resource for MAS and genomic selection in wheat. Guo et al. (2020b) used GBS1.0 DArT arrays and 90K iSelect SNP array to map QTL for protein and starch-related traits. The authors reported 26 stable QTL, for GPC, sedimentation volume, DDT, DST, FWA, flour viscosity, break down, and peak time, detected in more than two environments on chromosomes 1A, 1B, 1D, 4B, 5D, 6A, 6B, 6D, 7A, and 7D. These genomic regions were linked to several candidate genes, including embryonic flower 1 (*EMF1*), trehalose-6-phosphate synthase 6 (*TPS*), nitrate transporter 1:2 (*NRT1; 2 transporters*), zinc-finger protein 830 (*ZNF830*), phospholipid-transporting ATPase, transcription factor (*TFIIIB)*, *acylamino-acid-releasing enzyme* (*APEH*), F-box protein (*SKIP22*), and *aldehyde dehydrogenase* (*ALDH*). Similarly, Jin et al. (2016) used the 90K iSelect and the 660K SNP arrays to identify QTL for dough rheology and starch-pasting properties (Jin et al., 2016). Subsequently, 119 additive QTL were mapped on 20 chromosomes (i.e., all except 4D), including 55 and 17 novel QTL for mixolab parameters and 17 for Rapid Visco-Analyzer parameters. SNP markers in these regions were located on eight candidate genes, involved in biosynthesis of fatty acids and amino acids, in addition to starch and sucrose metabolism, i.e., anthranilate phosphoribosyltransferase (*AnPRT*), 3-ketoacyl-CoA, ornithine aminotransferase, lipoxygenase 2 (*LOX2*), sucrose-phosphate synthase II (*SPS2*), lysosomal beta glucosidase, and 5′-methylthioadenosine/S-adenosylhomocysteine nucleosidase (*mtnN*; Jin et al., 2016). Using the power of GBS, Boehm et al. (2017) identified co-localizing QTL for multiple end-use quality traits (GPC, FWA, and flour yield) on chromosomes 1B, 2D, 7A, and 7B, including allelic variation for the glutenin genes *Glu-A1*, *Glu-B1*, *Glu-A3*, *Glu-B3*, and *Glu-D3*.

## Current and Potential Methods to Identify and Clone Genes in Wheat

The availability of reference genome in wheat and subsequent construction of several high-density genetic maps developed from the sequence-tagged SNPs (see section Characterization of Genes and Gene Families Using the Wheat Reference-Genome) has opened new opportunities for map-based cloning of the genes. Therefore, here we have discussed the presently used and potential gene cloning methods in bread wheat. Dissecting the genetic and molecular mechanisms regulating grain yield and growth is the key for positional cloning or map-based cloning, as well as wheat breeding and improvement. Traditional forward genetic tools have been widely used to clone genes regulating traits of interest in wheat, e.g., *VRN1* (Yan et al., 2003), *Gpc-B1* (Uauy et al., 2006), and *Lr21* (Huang et al., 2003). Map-based gene cloning, however, usually needs multiple steps such as generating mapping populations, fine mapping to narrow the target region to identify genetic markers co-segregating with the phenotype, screening candidate gene(s) and gene(s) identification by sequencing. This process often requires more time and is labor intensive, especially in wheat. Hence, a limited number of positional cloning studies have been successfully undertaken. Furthermore, BREEDWHEAT program^10^ summarized 8-year efforts in which novel source of genetic diversity identified and introduced to elite materials to generate superior varieties.

Bulked segregant analysis (BSA) was recommended as a shortcut to identify the linkage of molecular markers with phenotype, being extensively used to map loci that have major effects (Michelmore et al., 1991). In this analysis, DNA of each individual showing extreme phenotype in a segregating population (i.e., F2) are bulked and genotyped, including their parents, with molecular markers (Chhetri et al., 2017). Any marker is considered to be linked with the studied trait if it shows the same allele in the bulk and parent of a similar phenotype. Recently, with the great advances of NGS technologies, several BSA-based modifications have been developed to identify major-effect QTL, regulating quantitative traits. These modifications are based on whole-genome resequencing bulks in a large population, reducing cost of genotyping, time spent, and increasing statistical power of analyses (Zou et al., 2016; Chhetri et al., 2017).

In crops with large genomes such as wheat, complexity reduction is very important to identify and clone target genes more quickly and efficiently. QTL-seq is one such approach that incorporates the potential of BSA. The power of high-throughput whole-genome resequencing to identify genomic regions showed contrasting results of an SNP index in two bulk populations (each with 20–50 individuals), featuring extreme phenotypes (Takagi et al., 2013a). Recently, QTL-seq was used in bread wheat to identify the candidate genomic region tightly linked to the awn inhibitor loci. Diagnostic markers were designed to understand the role of QTL in the awnless trait formation (Wang et al., 2021). Moreover, this approach was applied to identify loci involved in tiller angle in bread wheat, which represents an important factor influencing yield. Also, in this case, functional markers for MAS were developed and validated (Zhao et al., 2020). Multiple QTL-seq (mQTL-seq) used for several mapping populations from crosses with at least one common parent (Das et al., 2015). The use of multiple mapping populations with a broad genetic diversity was critical for the validation of QTL, along with narrowing down the detected QTL. To date, however, this technique has not yet been used in wheat.

Where the cost of whole-genome resequencing becomes prohibitive, bulk segregant RNA sequencing (BSR-seq) can be an alternative strategy for identifying expression QTL (eQTL) regions, generating data of gene expression for genomic loci of interest (Trick et al., 2012). Differential expression of genes in two bulks can be used to identify candidate genes responsible for favorable phenotypes. BSR-seq has been successfully used for mapping of stripe-rust-resistant loci *YrMM58* and *YrHY1* on chromosome 2AS (Wang et al., 2018b), *Yr15* on chromosome 1BS (Ramirez-Gonzalez et al., 2015), and leaf senescence gene (*els1*) on chromosome 2BS (Li et al., 2018a), in segregating wheat biparental populations. Similarly, BSR-seq enabled fine-mapping of a locus controlling grain-protein content (GPC) in wheat (*GPC-B1*) to 0.4 cM from the previously reported interval of 30 cM (Trick et al., 2012). This study pinpointed 13–18 candidate genes for GPC in wheat.

NGS platforms have also accelerated the identification and cloning of genes in mutant collections. TEnSeq pipelines are examples of advances that have allowed for rapid gene cloning identification, as recently reviewed (Zhang et al., 2020). Mutagenesis chromosome flow-sorting and short-read sequencing (MutChromSeq) is a recently developed tool (Sánchez-Martín et al., 2016; Hiebert et al., 2020), based on mutagenesis followed by flow sorting of chromosomes and their subsequent sequencing, to identify induced mutations. This rapid approach was successfully described to clone the powdery-mildew resistance locus *Pm2* in wheat (Sánchez-Martín et al., 2016). MutChromSeq has the advantage that it does not rely on an assumption that the resistance gene belongs to the NLR class (as for other approaches, see below). Hence, it would be appropriate for identification of non-immune-mediated resistance genes. The most recent application is the cloning of the Med15 protein encoded by *SuSr*-*D1*, a suppressor gene of stem-rust resistance (Hiebert et al., 2020) from the wheat cultivar “Canthatch.”

A similar approach to MutChromSeq, which does not require mutagenesis, is the target chromosome-based cloning (TACCA) method. It uses flow-sorted chromosomes, next-generation sequencing, and cultivar-specific *de novo* assembly. Using this approach, *Lr22a*, broad-spectrum leaf-rust resistance locus was cloned in wheat. Two SSR markers flanking *Lr22a*, covering 0.48 cM interval on chromosome 2D, were previously mapped. Sorting chromosome 2D, followed by sequencing and identification of genes, was performed within 4 months (Thind et al., 2017).

Other cloning strategies such as MutMap (mutational mapping) involve mutagenesis, sequencing, and mapping, to identify SNP between wild-type and homozygous mutants, and then zero in on the region containing the gene of interest. Although this approach was initially considered to be applicable only in crops with small genomes, it was successfully utilized to map and clone *Ms1* from bread wheat, using F2 plants derived from heterozygous *ms1e* mutants (Wang et al., 2017). MutMap will be less efficient at identifying the causal mutation, however, if the wild-type reference genome has gaps at the position of the causal mutation. Thus, *de novo* assembly of the wild-type genome is used in MutMap-Gap (Takagi et al., 2013b), and could be applied to wheat in the future.

Finally, MutRenSeq is a fast gene-cloning tool for the isolation of nucleotide-binding and leucine-rich repeat (NLR) genes (Steuernagel et al., 2016). Chemical mutagenesis, exome capture, and sequencing are required. Most genes associated with disease-resistance encode proteins in the NLR family. Hence, exome capture is necessary to enrich the NLR-specific bait library. Then, resistant wild-type parent and susceptible loss-of-function mutants are sequenced, as a last step. Mutant reads are aligned with the equivalent wild-type pool of the NLR gene family from the parents. This method has been utilized to clone two fungal stem-rust resistance genes (*Sr22* and *Sr45*) and three yellow-rust genes (*Yr7*, *Yr5*/*YrSP*) from bread wheat (Steuernagel et al., 2016; Marchal et al., 2018). This method does not require fine positional mapping and can be applied to isolate NLR-type resistance genes from most crops and their wild relatives. Nevertheless, two major limitations must be taken into account. Firstly, the design of oligonucleotide baits is based on a reference genome sequence. Considering the large-scale presence/absence variations among different accessions, the recent release of pan-genome in wheat is the ideal reference on which to design baits (Walkowiak et al., 2020). Secondly, this approach is limited to isolating only resistance genes, encoding NLR proteins. Therefore, genes that do not belong to the NLR family are missed (Dinh et al., 2020). However, it is possible to add capture baits targeting other classes of genes, thought *a priori* to be involved in disease resistance, such as wall-associated kinases.

Unlike map-based cloning and MutRenSeq, the association genetics with the R-gene enrichment sequencing (AgRenSeq) method has been developed to align with GWAS platforms (to utilize genome-wide natural variation). Thereby, RenSeq eliminates the need for biparental mapping populations or mutagenesis. This approach was demonstrated successfully to clone the, *Sr46* R-gene and to identify the candidate-gene sequence for *SrTA1662*, using a diverse panel of *A. tauschii* ssp. *strangulata* (Arora et al., 2019). It explores a pool of diverse wild relatives, carrying many resistance genes. As a result, it enables cloning of multiple genes at the same time (Dinh et al., 2020). These strategies have many advantages over traditional marker-based mapping. In addition to taking much less time, they find genes or functional nucleotides/haplotypes, responsible for a given agronomic trait. In several cases, genes were rapidly cloned and diagnostic markers were developed. The benefits of cloning genes in wheat, particularly those with a role in disease resistance, have been recently shown (Luo et al., 2021). As demonstrated by Luo et al. (2021), construction of transgene cassettes can simplify breeding bottlenecks, associated with the deployment of multiple genes to be inherited as a single unit. In this case study, a gene cassette containing five previously cloned R-genes (*Sr45*, *Sr50*, *Sr55*, *Sr22*, and *Sr35*) provides high levels of resistance to stem rust, suggesting that this could be a viable solution to confer durable multi-pathogen resistance.

## Genomic Selection in Wheat to Improve Complex Traits

The relatively recent availability of large numbers of genome-wide molecular markers in wheat genetic resources has led to the application of an alternative marker-assisted approach for wheat genetic improvement, namely genomic selection (Meuwissen et al., 2001; Jannink et al., 2010). For a long time, the lack of high-density markers were a major hindrance to carrying out in-depth genetic and genomic analyses. GS is an advanced form of MAS, wherein genome-wide markers are used to calculate genomic-estimated breeding values (GEBV; Meuwissen et al., 2001). Rather than explicitly identifying and tracking markers associated with genetic loci controlling a given trait, GS aims to use large numbers of genome-wide markers, in conjunction with phenotypic data collected in a collection of lines/varieties (termed the “training set”), to establish parameters that allow forward selection of the progenies derived from the training set over multiple forward generations, in the absence of additional phenotyping (Meuwissen et al., 2001; Jannink et al., 2010). This potentially allows selection to be applied faster and at higher intensities, as more lines can be incorporated for advancing to subsequent generations, without the need for time-consuming phenotyping steps. Additionally, advancement in statistical and bioinformatics methods to deal with high-density marker data for genomic selection has been equally important for plant breeders for the development of GS in wheat. GS is a valuable and attractive plant breeding approach that provides an idea for the conversion of genotypic value to phenotypic value (Nakaya and Isobe, 2012). Although this approach has great potential, plant breeders must carefully consider relationship between training and breeding populations. Additionally, it is important that breeders should investigate all traits of interest during the training phase in order to exclude phenotyping during the breeding cycle.

Since its first use in 2006 (Crossa et al., 2006), GS has been extensively used in wheat, for a wide range of traits with different architectures including grain yield (Crossa et al., 2016; Rutkoski et al., 2016; Saint Pierre et al., 2016; Guo et al., 2020a; Sehgal et al., 2020b), resistances to different diseases as rusts, fusarium head blight, *Stagonospora nodorum* blotch*, Septoria tritici* blotch, and tan-spot resistance (Jiang et al., 2017; Juliana et al., 2017a,b), macro- and micro-nutrients (Manickavelu et al., 2017), as well as end-use quality traits (Battenfield et al., 2016; Kristensen et al., 2018). Application of GS for hybrid prediction has also been investigated in wheat (Zhao et al., 2013b; Adhikari et al., 2020), pointing to challenges when predicting hybrids derived from untested parents (Zhao et al., 2015). Using more refined Genotype x Environment (GxE) interaction-based GS models promises to partly reduce this shortcoming (Basnet et al., 2019). The optimization of underlying factors on which genomic selection relies, such as marker density, predictive models, training population size, and the relationship between training and validation population sets, is ongoing (Larkin et al., 2019).

Recent investigations have focused on optimization of GS in genetic resources. In order to harness new diversity from wheat gene banks. Crossa et al. (2016) investigated GS models to predict days to heading and days to maturity, on a large set of wheat landrace accessions (8,416 Mexican landrace accessions and 2,403 Iranian landrace accessions) from CIMMYT gene bank, using two strategies. The first one involved random cross-validation of the data in 20% training (TRN) and 80% testing (TST; TRN20-TST80). In the second strategy, two types of core sets called “diversity” and “prediction,” including 10 and 20%, respectively, of total collections were used. Prediction accuracy of the 20% diversity core set was close to accuracies obtained for 20% training and 80% testing set (0.412–0.654 and 0.182–0.647 for Mexican landraces and Iranian landraces, respectively). For traits controlled by a mix of a few major and many minor genes, it can thus be beneficial to include preexisting knowledge on known candidate genes, to increase accuracy of genome-wide predictions (Bernardo, 2014). The potential of such an approach has been demonstrated when predicting flowering time and plant height for wheat (Zhao et al., 2014). These results suggested a way forward for parental selection in pre-breeding, by predicting the value of all genotyped accessions in a gene bank, followed by pre-breeding programs, based on those genotypes that have the highest predicted value, or harbor promising novel candidate genes or alleles. Once promising parents are identified, efficient pre-breeding programs need to be designed. This is a non-trivial task, mainly depending on diversity of plant genetic resources. Some of the latter have diversity that makes them directly useful as a source of parents for breeding new varieties; however, this tends to be the exception. It is evident that genetic resources must therefore be improved, through appropriate pre-breeding programs, to the point where they can be used as productive parents in breeding programs. Two complementary approaches can be used: (i) pre-breeding of populations created from genetic resources and (ii) pre-breeding of populations created from crosses between plant genetic resources and elite materials. In both cases, genomic prediction enables rapid selection gain, but requires the presence of extensive training populations, related to the base population. Crossing parents selected from agronomic and physiological screening of genetic resources, and advancing through generations, using high throughput phenotyping of physiological parameters, is another approach (Reynolds and Langridge, 2016). Such strategy is complementary to genomic selection of progeny, since many complex physiological traits that may have been used in strategic crossing do not lend themselves to high-throughput progeny screening (Reynolds and Langridge, 2016).

Substantial efforts have shifted to development of high-throughput phenotyping platforms in wheat, which are used to measure different traits, including plant height (Holman et al., 2016), disease resistance (Devadas et al., 2015), growth rate (Holman et al., 2016), and nitrogen deficiency (Devadas et al., 2015). These significant advancements in high-throughput phenotyping have brought a paradigm shift in breeding strategies. Wheat scientists have incorporated high-throughput phenotyping data in GS models, to explore their potential in improving prediction accuracies for complex traits (Rutkoski et al., 2016; Crain et al., 2018). Rutkoski et al. (2016) investigated the role of canopy temperature and green and red normalized difference vegetation index (NDVI), measuring chlorophyll concentration, canopy leaf-area and yield, as secondary traits in GS models, for improving prediction accuracy for grain yield. The authors observed 67% improvement in prediction accuracy, without correcting for days to heading (DTH), and 37% improvement upon correction with DTH. Crain et al. (2018) used over 1.1 million phenotypic data points generated by high-throughput phenotyping on 1,170 advanced CIMMYT lines in drought and heat stress environments, observing an increase in prediction accuracy from 7% to 33%, as compared to the standard univariate model.

Genomic selection has revolutionized animal breeding and will likely be a major source of genetic improvement of crops, including wheat, over the coming decade (Mackay et al., 2021). We envisage that incorporation of additional datatypes and technologies into GS pipelines will open opportunities for further gains to be made. For example, increasing precision of phenotypic characterization in training set *via* high-throughput phenotyping platforms (Devadas et al., 2015; Holman et al., 2016; Rutkoski et al., 2016; Crain et al., 2018), as well as incorporation of environmental covariates (de los Campos et al., 2020), may lead to improved prediction accuracies. Similarly, incorporating additional molecular or “omics” data may further refine prediction equations. These include: (i) molecular data tagging functionally validated alleles, whether they are natural variants, or novel ones, generated *via* technologies such as gene editing or TILLING (Krasileva et al., 2017; Hussain et al., 2018) and (ii) integration of transcriptomic and metabolomic data with molecular markers, as has been reported in maize (Schrag et al., 2018). Ultimately, combining these approaches with methods for shortening of wheat generation times will further increase selection intensity. Currently, “speed breeding” methodology, whereby plants are grown under extended photoperiods *via* the use of supplementary lighting, allows spring wheat generation time to be reduced from four to around 2 months (Watson et al., 2018). Combining speed breeding with “speed vernalization” methods can even more shortened the breeding process. Any further dramatic shortening of cycling times would require development of new approaches, such as generation of recombinant individuals, by *in vitro* production of gametes and their subsequent fusions (De La Fuente et al., 2013). Combining such *in vitro* generation-cycling with genomic selection methodologies may well represent an achievable medium-term step-change in genomics-informed breeding.

## CRISPR/Cas9-Mediated Genome Editing for Wheat Improvement

Availability of complete genome assemblies of diverse wheat genotypes, originating from different parts of the world, is essential to identify and characterize functions of various wheat genes, for different growth stages and environmental conditions, at the whole-genome level. Furthermore, transcriptomic analyses help to identify genes and gene networks regulating traits in different conditions. Therefore, knockout, knock-in, or activation of such genes through CRISPR/Cas9 gene-editing system provides unique opportunities for wheat genetic improvement (Scheben et al., 2017; Scheben and Edwards, 2017; Hussain et al., 2018). Because of the very large genome size of such species, orthologous gene copies present in the polyploid genome, and the presence of many repetitive sequences, genetic manipulation through CRISPR/Cas9-mediated gene editing system is more challenging in bread and durum wheat, as compared to cereals with smaller genomes. Using wheat-cell suspension cultures which led to InDel mutations, an attempt was made to use the CRISPR/Cas9 method for specific gene modification, in wheat inositol oxygenase (*inox*) and phytoene desaturase (*pds*) genes (Upadhyay et al., 2013). The first successful application of CRISPR/Cas9 to generate wheat knockout lines having three homoeoalleles of powdery-mildew resistance locus O gene (*TaMLO*), by a transient protoplast expression system, was done independently by Shan et al. (2014) and Wang et al. (2014). The latter successfully applied the CRISPR/Cas9 system in bread wheat, for the generation of plants mutated in a single *TaMLO-A1* allele, with increased resistance to powdery mildew. A similar strategy has been used to knockout drought-responsive transcription factors in wheat, like dehydration-responsive element-binding protein 2 (*TaDREB2*) and ethylene-responsive factor 3 (*TaERF3*), for improved drought signaling (Hussain et al., 2018; Kim et al., 2018).

The knockout of all three homoeoalleles of *TaGW2* through the CRISPR/Cas9 system increased the thousand kernel weight (TKW) and seed size (Wang et al., 2018a), implying the utility of the system for crop improvement. Such CRISPR-generated lines can either be exploited as new varieties or used as germplasm. Moreover, recent advances in editing allow simultaneous multiple-gene targeting or genome multiplexing (Xie et al., 2015), opening new horizons for employment of CRISPR/Cas9 in polyploid wheat, carrying many homoeologous and paralogous copies of the same gene, such as α-gliadins. In another study, CRISPR/Cas9 mediated mutations in 35 out of 45 α-gliadin genes, genes controlling gluten content in wheat, generated transgene-free, low-gluten wheat without any off-target mutations (Sánchez-León et al., 2018). Using this genome multiplexing by CRISPR/Cas9, three genes, *viz*., *TaGW2* (grain traits negative regulator), *TaMLO* (resistance to powdery mildew), and *TaLpx-1* (lipoxygenase; offers resistance to *Fusarium graminearum*), were targeted (Wang et al., 2018a). The first application of zinc-finger nuclease (ZFN)-mediated, non-homologous end joining (NHEJ)-directed loss-of-function gene knockout of acetohydroxyacid synthase (*AHAS*) in allohexaploid bread wheat through a supplied DNA repair template resulted in resistance to imidazolinone herbicides, due to an amino acid change in the target-gene coding sequence (Ran et al., 2018). Efficient and novel ribonucleoprotein-based (RNP) CRISPR/Cas9 genome editing procedures that required only 7–9 weeks were developed, with no off-target mutations and no transgene integration, implying the efficiency of the system (Liang et al., 2017).

The CRISPR/Cas9 genome editing methodology is also important for pre-breeding, namely, to reduce time to transfer beneficial alleles, increasing success rate. The idea is to directly induce/modify the alleles to beneficial ones in elite wheat germplasm both efficiently and quickly. CRISPR/Cas9-mediated permanent genome integration results in a stable expression of CRISPR/Cas9. However, RNP-based biolistic delivery offers a transient expression of CRISPR/Cas9, and its rapid degradation, which controls off-target mutations (Liang et al., 2017). Thus, RNP-based gene editing has been successfully applied for gene-editing in bread wheat (Liang et al., 2018). Similarly, knockout of three homologs of wheat enhanced disease resistance 1 (*TaEDR1*), a negative regulator of defense response against powdery mildew, conferred resistance against powdery mildew, without any off-target mutations (Zhang et al., 2017). DNA-virus [e.g., *Geminivirus*, i.e., wheat dwarf virus (WDV)]-based amplicons were later identified as an efficient construct-delivery method for gene editing, with an enhanced CRISPR/Cas9 expression, as compared to ubiquitin reference gene, proposing that it could be a potential tool for CRISPR-mediated genome editing in wheat (Gil-Humanes et al., 2017). Moreover, CRISPR/Cas9 has been successfully applied by generating heritable, targeted mutations, in wheat male-sterility 1 gene (*Ms1*), responsible for complete male sterility in commercial wheat cultivars, like Gladius and Fielder (Okada et al., 2019), thus speeding up hybrid-wheat production. These studies demonstrate the utility of the CRISPR/Cas9 system for rapid generation of male sterility in commercial wheat cultivars, for breeding programs. Although CRISPR-mediated genome or gene editing was demonstrated to be successful, its widespread implementation still encounters difficulties, involved in low regeneration efficiency of crops, such as wheat. Recently, growth-regulating factor-grf-interacting factor (GRF-GIF) wheat transformation system has become the game changer by using GRF-GIF chimeric protein construct, which improves regeneration efficiency up to 100% (Debernardi et al., 2020) in the transgenic-wheat lines.

Again, such CRISPR-generated lines can be released either as a variety or can be used as germplasm stocks. Although the utility of this revolutionary technology for crop improvement was demonstrated, regulatory approvals for the use of gene-edited plants still vary among different countries (Jouanin et al., 2018). CRISPR can also be utilized for testing the effect of a mutated allele on the resulting phenotype. If regulations are too strict, like in the European Union, this could be used later on, to search for such alleles in natural populations of wheat progenitors and germplasm stored in gene banks (“natural variation”). Recent initiatives to sequence thousands of gene bank accessions (Milner et al., 2019) can help to facilitate this approach. An additional benefit of using CRISPR/Cas9 is that genome editing for the first time allows direct transfer of favorable alleles into elite breeding material, without typical linkage drag, associated with cross-breeding. Wheat is crossed with maize to induce haploids, and colchicine is applied to get doubled haploid plants that serve as breeding material, or could be introduced as a variety, thus speeding up wheat breeding (Devaux and Cistué, 2016). Taking advantage of the well-established wheat × maize crossing system, maize pollens carrying gRNA for plant height genes (*BRI1* and *SD1*) were crossed to wheat, for inducing site-directed targeted mutagenesis in wheat (Budhagatapalli et al., 2020), without the need of segregating out the transgene. It helped in reducing the genotype-dependent site-directed mutagenesis. This approach can also be used for introducing mutations in multiple genes with one *cas9*/gRNA-transgenic (pollinator) plant, thus providing an opportunity for multiplex gene-editing in wheat. An important part of genetic analyses is identification of candidate genes and/or diagnostic marker(s) in linkage equilibrium to the trait(s) of selection interest. Many studies for abiotic stresses lack identification of candidate genes, slowing down MAS in wheat.

## Concluding Comments

Reviewing the very extensive genome-level analyses undertaken since publication of the CS reference genome sequence has identified the broad importance of considering the network nature of grain yield, heat and drought susceptibility indices and yield stability coefficients (Sehgal et al., 2017). Likewise, their association with flowering time genes (*Vrn-B1*, *Ppd-D1* and *Vrn-D3*). Observations are consistent with the long history of wheat improvement through breeding at the phenotypic level, and genome-level analyses can now complement this existing knowledge, through refining biological networks and fine-tuning germplasm to micro-environments and defined wavelength environments of LED lighting in speed breeding.

Typically, candidate genes are identified by locating the QTL region on the genome assembly and analyzing the genes residing in the region. If only a few genes are selected to validate their expression under a certain condition, the possibility of human bias (Baxter, 2020) can come into play, slowing down progress of genetic advancement. For example, looking for K^+^/Na^+^ transporters for salinity studies could lead to ignoring important genes in tolerance mechanisms. Complementing genomic selection with *in silico* transcriptomic analyses for all potential genes of interest would cast a wider net to capture a more complete set of genes that are relevant to the phenotype under study or selection.

The role of synthetic wheat in imparting stress tolerance is well known in wheat. Wheat gene banks harbor hundreds of such synthetic wheat lines. In the post reference genome era, extensive genotyping efforts have been undertaken to genotype entire gene bank accessions, generating the so-called “digital gene banks.” For example, CIMMYT has generated GBS data on ~100K accessions stored in its gene bank, in order to bridge the gap between genetic resources and breeding pipelines. Although success has been achieved in quantifying genome contributions of wild germplasm (synthetics and landraces) to the current elite germplasm, it is still unknown how to devise a genome-based strategy to deploy favorable introgressions from synthetic wheat, to enhance breeding value. Development of new genomic selection and machine-learning models and tools will be required to predict the best exotics from gene banks, without having to invest in laborious and costly multi-environmental field testing. These technologies will refine the already-successful breeder pipelines, for establishing new varieties.

Complex metabolic engineering can be exploited to improve cereals like wheat, to produce essential polyunsaturated fatty acids (PUFA), including the healthy omega 3 (ω-3) and omega 6 (ω-6). Genetic engineering and synthetic biology tools can be used to reach such a goal (Kraic et al., 2018). Similarly, these technologies can be used to generate Marker-Free and Transgene Insertion site-Defined (MFTID) transgenic plants. Thus, the lipoxygenase (*LOX*) gene expression was repressed using an RNA interference (RNAi) cassette reduce lipid peroxidation (improving storability) and increase nutrient quality, such as the amount of healthy fatty acids (e.g., containing linoleic and linolenic residues) of wheat seeds (Cao et al., 2020).

Cereals can also be improved so that their agricultural waste (plant cell wall, being mainly made of cellulose, hemicellulose, and lignin) contains more cellulose and less lignin, thereby allowing its use as feedstock for biofuel or bioproduct production. Currently, high costs and low yields are associated with their use, due to the molecular structure of the natural lignocellulosic biomass. As expected from an evolutionary point of view, it is hard to enzymatically hydrolyze cellulose into glucose, as it is resistant to most microorganism degradation (Rocha-Meneses et al., 2020).

In short, these developments hold interesting potential applications for wheat improvement, within molecular breeding programs, in addition to enhancing yield traits, and biotic- and abiotic-stress tolerance, which are particularly relevant in the present scenario of global warming and climate change.

## Author Contributions

HB conceived, designed, and organized the study. All authors listed have made a substantial contribution to the work, drafted, edited, and approved it for publication.

## Funding

HB was funded by USDA-NIFA SBIRI and SBIRII. PH and SG were funded by project P18-RT-992 from Junta de Andalucía (Andalusian Regional Government), Spain (Co-funded by FEDER). JC was funded by BBSRC grant BB/P010741/1. MF is supported by the 2Blades Foundation and Grains Research and Development Corporation (project CSP1801-013RTX/9176010). VK is supported by the Ministry of Science and Higher Education of the Russian Federation (grant no. 075-15-2019-1881). GH was supported by funding of the Deutsche Forschungsgemeinschaft (DFG, German Research Foundation) under Germany’s Excellence Strategy—EXC-2048/1—project ID 390686111 and grants 426557363 and 458717903, the European Regional Development Fund (Project ID ZS/2018/06/93171), and the Czech Science Foundation (CZ.02.1.01./0.0/0.0/16_019/0000827, SPP 813103381). BK thanks the Government of Norway (QZA-14/0005) for funding the initiative of “Adapting Agriculture to Climate Change: Collecting, Protecting and Preparing Crop Wild Relatives” (https://www.cwrdiversity.org/project/pre-breeding/).

## Conflict of Interest

HB and BA are employed by Montana BioAg Inc., VK is employed by KWS, TU is employed by Ficus Biotechnology, and PD is employed by Florimond Desprez Group.

The remaining authors declare that the research was conducted in the absence of any commercial or financial relationships that could be construed as a potential conflict of interest.

## Publisher’s Note

All claims expressed in this article are solely those of the authors and do not necessarily represent those of their affiliated organizations, or those of the publisher, the editors and the reviewers. Any product that may be evaluated in this article, or claim that may be made by its manufacturer, is not guaranteed or endorsed by the publisher.

## Supplementary Material

The Supplementary Material for this article can be found online at: https://www.frontiersin.org/articles/10.3389/fpls.2022.851079/full#supplementary-material

## Footnotes

^1^ http://faostat.fao.org

^2^ https://wheat-urgi.versailles.inra.fr/Seq-Repository/Assemblies

^3^ https://www.wheatgenome.org/News2/IWGSC-RefSeq-v2.0-now-available-at-URGI

^4^ https://wheat.pw.usda.gov/WheatExp

^5^ http://www.wheat-tilling.com

^6^ http://plants.ensembl.org/Triticum_aestivum

^7^ https://plantinformatics.io

^8^ https://www.thermofisher.com/order/catalog/product/900560TS#/900560TS

^9^ https://arborbiosci.com/genomics/targeted-sequencing/mybaits/mybaits-expert/mybaits-expert-wheat-exome

^10^ https://www.mdpi.com/2079-7737/11/1/149
