# Supplementary material for: Capturing Wheat Phenotypes at the Genome Level
Source: Front Plant Sci. 2022 Jul 4;13:851079. doi: 10.3389/fpls.2022.851079 (PMC9289626; doi:10.3389/fpls.2022.851079)
Supplement: Supplementary file 1 [file Data_Sheet_1.docx]

**Supplementary File for**

**Capturing wheat phenotypes at the genome level**

**Supplementary S1: Yield and related traits in bread wheat**

GWAS using high-throughput genotyping arrays or GBS have been applied extensively (Table 1) to identify functional markers and candidate genes for yield and related traits (Lopes et al., 2015; Sukumaran et al., 2015; Zanke et al., 2015; Ward et al., 2019; Alqudah et al., 2020; Pang et al., 2020) (Table S1). Generally, many chromosomes are associated with the trait, while for other traits, only one chromosome is associated with the respective trait and that this reflects the repetitive occurrence of some genes and the limited homoeologous distribution of others (Appels et al., 2018). A GWAS for phenology-controlled population - Wheat Association Mapping Initiative (WAMI) - of 300 lines was conducted to identify yield and its components (TGW, GN, heading, NDVI, and adaptation to density) and earliness *per se* (Sukumaran et al., 2016). A more recent study on a high biomass population identified markers associated with above ground biomass and radiation use efficiency (Molero et al., 2018). Two independent GWAS (Lopes et al., 2015; Zanke et al., 2015) genotyped 287 and 372 wheat accessions, respectively, to identify 31 and 86 significant marker-trait associations (MTAs) for plant height, days to heading (DH), grain yield, TGW and photoperiodism, respectively. Most of these MTAs were located on chromosomes 1 (AB), 2 (ABD), 3 (ABD), 4 (BD), 5 (AD), 6 (ABD), and 7 (AB) while some of these MTAs for yield and related traits were linked to candidate genes/loci such as *Rht-B1, Rht-D1, Vrn-A1, Ppd-D1, TaSus1, TaSus2, TaGS-D1* and *TaGW2-6B* (Lopes et al., 2015; Zanke et al., 2015). Importantly, a GBS-based GWAS for 768 wheat accessions identified 395 QTL for plant height, DH, SNPP, spike length and number, grain length, grain width, TGW etc. under seven environments (Pang et al., 2020). These QTLs were closely linked with several candidate genes including, but not limited to, yield-related genes: *APO1, AUX1, Ehd2, GSN1, GL3, Gn1a, MADS14, MADS15, MADS18, MADS57, Rht-D1, Rht12, TaGA2ox8, TaSus1-7B, Vrn-B1* and *Vrn-D1*. Additionally, QTL and candidate genes on chromosomes 4B (GPN-loop GTPase-like protein, RNA-binding protein and 5A [auxin canalization protein (DUF828), pentatricopeptide repeat-containing protein, kinase interacting (KIP1-like) family protein] enhanced the spike seed stetting. Similarly, QTL and candidate genes (ubiquitin-conjugating enzyme E2, the protein of an unknown function (DUF2921)) on chromosome 1B enhanced grain size and TGW (Pang et al., 2020). The functions of these candidate genes were validated in three populations by transcriptomics analysis.

GWAS (Ward et al., 2019; Alqudah et al., 2020) based on GBS and the 15K SNP array genotyping identified 118 and 74 significant MTAs, respectively, for yield and related traits that associated with candidate genes such as *Gibberellin 2-oxidase 2 (GA2ox2), Pre-rRNA-processing protein TSR2, Glutathione peroxidase (GPx), ATP-dependent zinc metalloprotease (FtsH 1), mother of flowering time and terminal flowering 1 (MFT1), WAPO1*, B2 heat stress response protein, and gibberellin oxidase protein (GA2ox-A1) as detailed in Table S1. Furthermore, total 27 QTLs were combined to develop a consensus map consisting of 140,315 markers and 376 QTL, including 221 for grain yield-related traits. The projection of this map and the relevant QTL onto the wheat syntenome (having 99,386 genes) identified 32 metaQTL (mQTL), including 18 grain yield mQTL associated with 15,772 genes (28,630 SNPs), 37 of which were major candidate genes (Quraishi et al., 2017)including *ATPase, GIF1, Ppd-D1, Prog1, Gn1-a, NYC1, emp4, DEP1, GW2, GS2* and *Rc3*. Taken together, these studies illustrate the information provided through GWAS which can be used for improving wheat through MAS. However, a recent study involving 2,000 wheat accessions suggests that validation of MTAs in wheat remains a challenge (He et al., 2017) further research is needed before it can be widely used for wheat breeding.

Despite the large number of genetic mapping studies undertaken (Table S1), map-based positional cloning has been limited in wheat, largely because of the lack of a high-quality, fully annotated reference genome sequence. One of the earliest examples was the cloning of the vernalization gene, *VRN1* (Yan et al., 2003). Despite advances in cloning methods (Section 3), only a single QTL for an increase in grain number per spikelet, *GNI1*, has been cloned in wheat (Sakuma et al., 2019). However, sequences of several genes such as *TaGW2* (Su et al., 2011), cell wall invertase, *TaCwi-A1* (Ma et al., 2012), *TaGASR7-A1* (Dong et al., 2014), *TaGS-D1* (Zhang et al., 2014), *IAA-glucose hydrolase gene*, *TaTGW6* (Hu et al., 2016a) and *TaTGW-7A* (Hu et al., 2016b) have also been cloned for characterizing their roles in the improvement of yield and related traits.

Table S1: Examples of genomic regions, candidate and cloned genes for yield and related traits in bread wheat

| **Traits** | **Method** | **Chromosome** | **PV (%)** | **Candidate/cloned genes** | **Ref.** |
| --- | --- | --- | --- | --- | --- |
| Plant height (PH), anthesis, flag leaf, spike length (SL), spikelet (n)/spike | QTL | 2D, 4A, 4B, 6D | 10.1 – 30.7 | Genes for TGTCTC auxin response elements, F-box protein *TIR1*, Flowering Locus T-like protein, *MADS8*, *SAUR-like auxin-responsive proteins* | (Jin et al., 2020) |
| Spikelet (n) per spike | QTL | 7A | - | *WHEAT ABERRANT PANICLE ORGANIZATION 1* (*WAPO1*) | (Kuzay et al., 2019) |
| Thousand-grain weight (TGW), grain length | QTL | 7A | 9.3–19.7 | *TaGASR7* | (Su et al., 2016) |
| Grain number per spike | QTL | 4A | 8.0–21.2 | 65 Putative genes | (Cui et al., 2017) |
| Flag leaf length, width and area | QTL | 1A, 1B, 5A, 7A | 5.2–62.8 | *-* | (Hussain et al., 2017b) |
| Grain yield | QTL | 1A, 1D, 2B,3A, 3B, 4A, 5B, 6A | 32.7–69.7 | *Vrn-B1*, *Ppd-B1* | (Addison et al., 2016) |
| Plant height, grain yield, yield components, days to heading | QTL | 1A, 2A, 2B, 5A, 5B, 6A, 6B, 7A | 3.3–25.1 | *Heat shock 70 kDa protein, diacylglycerol kinase, alphamannosidase, DNA excision repair protein ERCC-6-like, auxin response factor 17 isoform X2, ubiquitin conjugation factor E4 B* | (Assanga et al., 2017) |
| Rachis nodes per rachis, TGW, days to heading (DH), | GWAS | 7A | - | *WAPO1, cyclin-like F-box* | (Voss-Fels et al., 2019) |
| Spikelet (n) per spike | QTL | 7B | 5-7 | *WAPO-B1* | (Corsi B, Obinu L, Zanella CM, Cutrupi S, Day R, Geyer M, Lillemo M, Lin M, Mazza L, Percival-Alwyn L, Stadlmeier M, Mohler V, Hartl L, 2021) |
| PH, grain yield (GY), DH | GWAS | 2D, 3A, 3B, 4B, 5A | 10–12 | *Rht-B1*, *Rht-D, Vrn-A1*, *Ppd-D1* | (Lopes et al., 2015) |
| TGW, Photoperiodism | GWAS | 1B, 2A, 2D, 3B, 3D, 4B, 4D, 6A, 6B, 6D, 7A | 2.3–14.4 | *Ppd-D1, Rht-B*, *Rht-D1, TaGW2, TaSus1, TaSus2, TaGS-D1* | (Zanke et al., 2015) |
| PH, DH, spikelet (n) per spike, spike length (SL), spike number (SN), grain length, grain width, TGW | GWAS | 1B, 4B, 6A, 7A, 7B | - | *WAPO1, AUX1, Ehd2, GSN1, GL3, Gn1a, MADS14, 15, 18, 57; Rht-D1, Rht12, TaGA2ox8, TaSus1-7B, Vrn-B1, Vrn-D1*, *GPN-loop GTPase-like, RNA-binding protein 34*, *DUF828*, *KIP1-like*, *ubiquitin-conjugating enzyme E2*, *DUF2921* | (Pang et al., 2020) |
| Spike sterility, PH, DH, spikelet (n) per spike, SL, SN, harvest index (HI), TGW, GY | GWAS | 1A, 1B, 2A, 2D, 3B, 5B | - | *Gibberellin 2-oxidase 2* (*GA2ox2*), Pre-rRNA-processing protein (*TSR2)*, *Glutathione peroxidase (GPx), ATP-dependent zinc metalloprotease (FtsH 1)* | (Alqudah et al., 2020) |
| Stay-green, PH, DH, GY, NDVI, grains per head, grains/m^2^, grain protein+ starch content, TGW | GWAS | 1A, 2A, 2D, 3A, 5B, 6A, 6B, 7A, 7D, | - | *Mother of flowering time and terminal flowering 1* (*MFT1*), Wheat-aberrant panicle organization (*WAPO1*) protein¸ B2 heat stress response protein, *gibberellin oxidase protein (GA2ox-A1*) | (Ward et al., 2019) |
| GY, TGW, grain number, PH, heading, NDVI, biomass, harvest index | GWAS | 1B, 1D, 2D, 3B, 5A, 5B, 6A | 5–10 | *-* | (Sukumaran et al., 2015) |
| Earliness per se | GWAS | 1D | 5–9 | *TaELF-3* | (Sukumaran et al., 2016) |
| Plant biomass, radiation use efficiency | GWAS | 5A, 7A | 7–17 | *Coleoptile phototrophism 1 (CPT1), BTF2‐like transcription factor, aldehyde dehydrogenase (ALDH), CPT1, guanosine diphosphate dissociation inhibitor (GDI2), early light‐inducible protein (ELIP), glutathione‐s‐transferase 3 (GST3)* | (Molero et al., 2018) |
| Grain yield | GWAS, GS | 1B, 2B, 3A, 4A, 4B, 5A, 5B, 6B, 7A, 7B, 7D | 1–4.5 | *-* | (He et al., 2017) |
| GY, Tillers (n), grain length (GL), grain width (GW), TGW, HI, PH, SL | mQTL | 1B, 1D, 2A, 2B, 2D, 3B, 3D, 4A, 4B, 5A, 5B, 6A, 6B, 7A, 7D | - | *ATPase, GIF1, Ppd-D1, Prog1, Gn1-a, NYC1, GARP, Lsk1, SRS5, emp4, DEP1, GW2, EP3 OsNaPRT1, GS2, Rc3, incw2, MOC1* | (Quraishi et al., 2017) |
| Grain number per spikelet | Cloned | 2A | 61 | *GNI1* | (Sakuma et al., 2019) |
| GW, TGW | Cloned | 6A | - | *TaGW2* | (Su et al., 2011) |
| TGW | Cloned | 2A | - | *TaCwi-A1* | (Ma et al., 2012) |
| GL | Cloned | 7A | - | *TaGASR7-A1* | (Dong et al., 2014) |
| TGW, GL | Cloned | 7D | - | *TaGS-D1* | (Zhang et al., 2014) |
| TGW | Cloned | 4A | - | *TaTGW6* | (Hu et al., 2016a) |
| TGW | Cloned | 7A | 21.7–27 | *TaTGW-7A* | (Hu et al., 2016b) |

GWAS = genome-wide association study, QTL = Quantitative trait loci, PV= Phenotypic variation, GS= Genomic selection

**Supplementary S2: Drought tolerance**

Drought is the most devastating abiotic stress curtailing productivity of all crops and in wheat, results in significant yield losses of up to 50% (Budak et al., 2015). Therefore, the development of drought-tolerant varieties has been a prime objective in global wheat breeding programs. Using traditional bi-parental mapping approaches, QTL for various agronomic and physiological traits responsive to drought stress have been genetically mapped (Table S2), and has been reviewed extensively (Budak et al., 2015; Gupta et al., 2017). The most common physiological traits that have been targeted for QTL mapping of wheat drought tolerance include, but are not limited to, canopy temperature, carbon isotope discrimination, chlorophyll content, water-soluble carbohydrates, ABA production, relative water content, stay-green trait, photosynthetic capacity/rate, cell membrane thermostability and importantly, various root architectural traits such as root elongation rate, primary root length, lateral root length, root angle, deep root ratio, root-shoot ratio, root biomass and deep root length (Edae et al., 2014; Iehisa et al., 2014; Shahinnia et al., 2016; Wang et al., 2016; Lucas et al., 2017; Sehgal et al., 2017; Shi et al., 2017; Xu et al., 2017; Soriano and Alvaro, 2019; Ballesta et al., 2020). For root related traits alone, more than 634 QTL have been reported and were projected onto a consensus map (Soriano and Alvaro, 2019). This study led to the identification of 94 consensus root mQTL, of which 35 were related to drought response and these mQTL were linked to 68 candidate genes (Table 2). Pleiotropic QTL linked to both physiological traits and yield-related traits have also been reported. For example, QTL for chlorophyll content, water use efficiency, photosynthetic rate, and internal CO_2_ concentration were co-located with QTL for GY and/or yield components (Xu et al., 2017).

Increased abscisic acid (ABA) levels have been suggested to impart drought tolerance in wheat by accelerating the accumulation of osmolytes (Budak et al., 2015). A major QTL for ABA responsiveness was mapped on chromosome 6D in an F_2_ population derived from a cross between synthetic wheat lines contrasting for ABA-responsiveness (Iehisa et al., 2014). This 6D QTL regulated the expression of *late embryogenesis abundant* (*LEA*) genes under drought conditions. A few studies have investigated the genetics of stomatal traits under drought stress by QTL mapping (Shahinnia et al., 2016; Wang et al., 2016). Most importantly, two QTL, one on chromosome 5A (Wang et al., 2016) and another on chromosome 7A (Shahinnia et al., 2016), were identified and both QTL were co-located with QTL of yield components or harvest index.

The stay-green trait (delayed leaf senescence) has been found to be correlated with adaptation to drought stress in wheat. Normalized difference vegetation index (NDVI) is used as an indirect selection criterion for stay-green and higher yield under drought in wheat (Gupta et al., 2017). Shi et al. (Shi et al., 2017) reported a major QTL for NDVI on chromosome 5A and several pleiotropic QTL for NDVI and agronomic traits on chromosomes 1B, 3D, 4D and 7A. Such pleiotropic regions shared by NDVI, biomass and yield component will aid breeders to utilize the trait as an indirect selection criterion for GY improvement. Consistent QTLs (2B, 4A, 7B) for drought and heat stress-related agronomic and physiological traits (stay-green, canopy temperature) were identified in a phenology controlled population of Seri/Babax (Pinto and Reynolds, 2015; Pinto et al., 2016). The genetic map was updated using 90K SNPs and DArTseq markers and QTLs were identified for GY, TGW, GN, NDVI, and CT (Liu et al., 2019a). Another study identified QTLs for heat and drought stress QTLs on a synthetic derived population with a flowering time range of 3 days (Liu et al., 2019b).

The drought sensitivity index (DSI) of agronomic traits, as an indirect measure of drought tolerance, has been used in QTL mapping studies in wheat as an indirect measure of drought tolerance (Shukla et al., 2015; Gahlaut et al., 2017; El-Feki et al., 2018). Gahlaut et al. (Gahlaut et al., 2017), for example, mapped DSI of nine drought-responsive agronomic traits in a DH population, which was evaluated under 22 environments in India under both irrigated and rain-fed conditions. Most importantly, the authors reported four stable QTL on chromosomes 5A and 7A. Additionally, two more studies identified stable QTL of agronomic traits under drought stress on chromosomes 3B (Shukla et al., 2015), 2B, 5A, 6A and 7D (El-Feki et al., 2018).

Due to the availability of high-density SNPs in the genomics era, GWAS became a leading approach for the dissection of complex traits such as drought tolerance in wheat. The vast majority of GWAS research in wheat has been done by mapping solely GY and yield components under drought stress (Sehgal et al., 2017, 2020b, 2020a; Ballesta et al., 2020); others have explored a wide range of physiological traits or a combination of physiological and yield-related traits (Zhang et al., 2013; Edae et al., 2014; Gahlaut et al., 2017; Shokat et al., 2020). Recently, root architectural traits including root/shoot dry weight ratio, root length and root biomass under drought stress have been extensively investigated by GWAS (Lucas et al., 2017; Soriano and Alvaro, 2019). An analysis of GWAS publications in wheat reveals that drought stress tolerance-linked MTAs have repeatedly been reported on chromosome 4A (Edae et al., 2014; Sehgal et al., 2017; Ballesta et al., 2020). Ballesta et al. (Ballesta et al., 2020), for example, mapped four types of indices (i.e., stress susceptibility index, stress tolerance index, tolerance index and yield stability index) based on GY and yield components on chromosome 4A. Edae et al. (Edae et al., 2014) identified MTAs for DSI, leaf senescence, green leaf area and flag leaf traits on chromosome 4A. Sehgal et al. (Sehgal et al., 2017) reported two stable QTL on chromosome 4A associated with GY under drought and heat stress environments.

With the availability of dense genome-wide markers from SNP arrays and other high-density wheat genotyping platforms (9K, 15K, 90K, 660K and 820K SNP arrays, genotyping-by-sequencing and DArTseq), the most recent investigations have also explored haplotypes-based GWAS approaches for identifying stable QTL for drought stress tolerance (Sehgal et al., 2020b, 2020a; Shokat et al., 2020; Yang et al., 2020b). Sehgal et al. (Sehgal et al., 2020a), for example, used a combination of haplotypes-based GWAS with epistatic interactions to untangle the genetic architecture of GY under multiple stress environments (including mild and severe drought stress) using a large panel of 6,333 advanced lines from the International Maize and Wheat Improvement Centre (CIMMYT). They reported four and ten stable haplotype association with grain yield under mild and severe drought stress environments, respectively. Most importantly, the authors identified a significant association of a haplotype block close to the *Vrn-B1* flowering time gene on chromosome 5B, with GY in more than 70% of the trials under severe drought stress. *Vrn-B1* is significantly correlated with adaptation to low temperature thus this shows a shared tolerance mechanism for both abiotic stresses.

Table S2: Examples of genomic regions and candidate genes for drought tolerance

| **Traits** | **Method** | **Chromosome** | **PV (%)** | **Candidate genes** | **Ref.** |
| --- | --- | --- | --- | --- | --- |
| Root related traits (root length, root width, root area, root angle, root volume, root weight, root dry weight, root diameter, root depth, root biomass, root growth, root:shoot) | mQTL | All | 1–76 | *E3 ubiquitin-protein ligases* (6), *F-box family proteins* (5), *LEAs* (4), *Root hair defective 3*- *RHD3* (3), *peroxidases* (3), *Glycosyltransferases* (2)*,* *Root primordium defective 1*, *Root meristem growth factor 1* | (Soriano and Alvaro, 2019) |
| Chlorophyll content (CC), photosynthesis rate, water use efficiency, CO_2_ concentration, grain yield (GY) | QTL | All;  Stable loci on 3A and 5B | 4.8–34.8 | – | (Xu et al., 2017) |
| Drought tolerance, ABA response | QTL | 6D | – | *TaABA8′OH1*, *CYCB2, CDKA1* | (Iehisa et al., 2014) |
| Stomata size, density, drought tolerance | QTL | 1D, 2D, 3A, 4A, 5A, 6A, 5B, 7A | 7.7–30.9 | – | (Wang et al., 2016) |
| Stomata size, density, grains per spike, VI, harvest index (HI), GY | QTL | 1A, 1B, 2B, 2D, 4B, 5A, 5D, 7A | 5–53 | – | (Shahinnia et al., 2016) |
| Stay green, PH, CC, thousand grain weight (TGW), GY, NDVI | QTL | 1B, 2A, 2B, 2D, 3A, 3B, 4B, 4D, 5A, 5B, 5D, 7D | 3.6–41.6 | – | (Shi et al., 2017) |
| Coleoptile length, root-shoot ratio, root length, volume & area, days to heading (DH), anthocyanin | QTL, GWAS | 1A, 4B, 6B | – | *Rht-B1* | (Lucas et al., 2017) |
| DSI, leaf senescence, leaf area, flag leaf traits, HI, TGW, GY | GWAS | 1A, 1B, 2D, 3A, 3B, 4A, 5A, 5B, 7A | 2.2–29.5 | *Rht8*, *Ppd-D1* | (Edae et al., 2014) |
| GY, susceptibility indices, DH, PH, GY stability coefficient (drought, heat) | GWAS | 1B, 2B, 3A, 3B, 4A, 4B, 5B, 6A, 6B, 7A, 7B, 7D | – | *Vrn*-*B1*, *Ppd-D1, Vrn-D3* | (Sehgal et al., 2017) |
| Stress Susceptibility/ Tolerance index (SSI, STI), yield stability index | GWAS | 1A, 1B, 2A, 2B, 3B, 4A, 5B, 7A, 7D | 4–6 | – | (Ballesta et al., 2020) |
| TGW, grain filling rate, stay-green, canopy temperature, NDVI | QTL | 1B, 2A, 2B, 4A, 4B, 7B, 7D | 8.4–36.4 | *SRG1* | (Pinto and Reynolds, 2015; Pinto et al., 2016) |
| GY, TGW, NDVI, days to heading | QTL | 1A, 1B, 2D, 3A, 3B, 4A, 4B, 4D, 5A, 5B, 5D, 6A, 6B, 6D, 7A, 7D | 4.1–21.3 | *Vrn-D4, Vrn-B3, Rht-B1, NST1-like protein, TPR15* | (Liu et al., 2019a, 2019b) |
| GY, biomass, grains (n), tillers (n), spikes (n), HI, SSI, flowering days, PH, TGW, canopy temperature | GWAS | All,  Stable loci on 3B, 4A, 6A, 7B | 15.6–19.6 | *Cytokinin oxidase* *(CKX2), indole acetic acid synthase* (*SPP1*) | (Shukla et al., 2015) |
| TGW, DH, grain filling duration (GFD), SSI | QTL | All, Stable loci on 5A, 7A | 2–35.8 | – | (Gahlaut et al., 2017) |
| GY, PH, GFD, DH, spike length (drought) | QTL | 1B, 2B, 5A, 6A,7B, 7D | 4.2–38.8 | *Ppd-B1, Vrn-D3* | (El-Feki et al., 2018) |
| GY, PH, DH, GY stability | GWAS | 3B, 4B, 6B, 7B | 5–16.3 | *Ppd-D1, Vrn-B1* | (Sehgal et al., 2020b) |
| GY (drought, heat stress) | GWAS, GS | 1A, 1B, 2B, 4A, 5B, 6B, 7B | 3.7– 23.1 | *Vrn-B1* | (Sehgal et al., 2020a) |
| PH, flag leaf, water-soluble carbohydrates | Association analysis | 5A | 3.7–5.6 | *TaSnRK2.8* | (Zhang et al., 2013) |
| GY, TGW, grains per spike | GWAS | 2D, 3A, 3B, 4A, 6B | – | – | (Shokat et al., 2020) |
| GY, TGW, HI, grains per spike, biomass yield | QTL | 1A, 1B, 1D, 2A, 2D, 3D, 4B, 4D, 6A, 6B, 6D, 7B, 7D | 10.3–12.2 | *TaGS-D1* | (Yang et al., 2020b) |
| Physiological parameters | QTL, RNAseq | 5A, 5B, 5D | – | *PSY3*, *NCED*, *VRN1*, *UGDH*, dehydrin *DHN38* | (Gálvez et al., 2019) |

GWAS = genome-wide association study, QTL = Quantitative trait loci, mQTL = metaQTL, PV= Phenotypic variation, GY = grain yield

**Supplementary S3: Heat tolerance**

Similar to drought stress, heat stress has been projected to become a major threat to wheat production in a changing climate. A 4–6% reduction in average global yields of wheat is predicted for each 1 °C increase in global mean air temperature (Asseng et al., 2015). Heat stress at reproductive stage resulted in 66% reduction in green yield in wheat (Jamil et al., 2019). In the past decade, multiple QTL mapping studies in wheat using bi-parental populations and different traits as indicators of heat tolerance have been reported (Table 3). Many of these studies consistently reported QTL hotspots on chromosome 3B (Bennett et al., 2012; Mondal et al., 2015; Sharma et al., 2017). Bennett et al. (Bennett et al., 2012), for example, identified two QTL for canopy temperature and GY on chromosome 3B. Sharma et al. (Sharma et al., 2017) identified a significant genomic region on chromosome 3B for F_v_/F_m_ trait (maximum quantum efficiency of photosystem II) in three mapping populations.

Along with chromosome 3B, QTL hotspots for heat tolerance have been identified on chromosomes 1B, 2B, 2D, 4A, 5A, 5B, 7A and 7D (Bennett et al., 2012; Talukder et al., 2014; Mondal et al., 2015; Bhusal et al., 2017; Sharma et al., 2017). Heat susceptibility or tolerance indices were used frequently in many of the above-cited studies (Bennett et al., 2012; Talukder et al., 2014; Bhusal et al., 2017). Several studies have reported common QTL for both heat and drought tolerance (Bennett et al., 2012; Acuña-Galindo et al., 2015; ElBasyoni et al., 2017; Sehgal et al., 2020a). Furthermore, two independent studies (Sharma et al., 2016; Bhusal et al., 2017) mapped several QTL for agronomic and physiological traits such as PH, GY, TGW, grains per spike, grain filling rate (GFR), grain filling duration (GFD) under heat stress, and heat susceptibility index onto chromosomes 1B, 2A, 2B, 3B, 5A, 6B and 6D, which contributed 11.2-30.6% of the phenotypic variation. Many of these QTL were mapped on chromosome 2A, located just 6 cM above the reported Fv/Fm (normalized ratio between variable fluorescence and maximum florescence) QTL, *QFv/Fm*.*cgb-2A* (Sharma et al., 2016; Bhusal et al., 2017). Other studies have also reported QTL for chlorophyll ﬂuorescence kinetics parameters (initial, maximum and variable fluorescence), chlorophyll content and Fv/Fm in addition to cytoplasmic membrane stability, proline content, water-soluble carbohydrates and grain yield (Azam et al., 2014; Hassan et al., 2018). Most importantly, a meta-analysis of all drought and heat tolerance QTL identified 66 metaQTL and candidate genes distributed throughout the genome (Acuña-Galindo et al., 2015). Of these, 20 and 2 were specific for drought and heat stress, respectively, while 43 metaQTL were for combined drought and heat stress on chromosomes 1B, 2B, 2D, 4A, 4B, 4D, 5A, and 7A. Such combined QTL identified for both drought and heat stress tolerance can be efficiently utilized efficiently in marker-assisted breeding.

The GWAS approach to dissecting heat tolerance in wheat has gained importance in the past two years (Sehgal et al., 2017, 2020a; Tadesse et al., 2019). Tadesse et al. (Tadesse et al., 2019) explored 197 spring wheat genotypes from the International Center for Agricultural Research in the Dry Areas (ICARDA) under the heat-stressed environments in Sudan and Egypt. Through MTA and GWAS, the authors identified astable genomic regions on chromosome 4A and 5A associated with yield at both geographical locations. Furthermore, they delineated a suitable marker combination, with one marker each from chromosomes 4A and 5A which together resulted in a yield advantage by 15%. Sehgal et al. (Sehgal et al., 2020a) identified 15 stable haplotypes associated with grain yield under heat stress environments by haplotypes-based GWAS in a large panel of advanced lines from CIMMYT and reported a haplotype block hotspot region for heat tolerance on chromosome 7A. Similarly, MTAs for grain yield, heat and drought susceptibility indices and yield stability coefficient was also reported (Sehgal et al., 2017) and were associated with the flowering time genes *Vrn-B1, Ppd-D1* and  *Vrn-D3*. To add to this, another study reported MTAs for spike ethylene content under heat stress conditions on the WAMI population (Valluru et al., 2017).

Table S3. Examples of genomic regions and candidate genes for heat tolerance

| **Trait(s)*** | **Cross** | **Pop.**  **Type** | **Pop. Size** | **LGs** | **Candidate genes** | **Ref.** |
| --- | --- | --- | --- | --- | --- | --- |
| Grain yield | RAC875 × Kukri | DH | 255 | 3B, 3D, 4D, 7A | - | (Bennett et al., 2012) |
| Flag leaf cuticular waxes, temperature depression of flag leaf and main spike | Halberd × Karl92 | RIL | 121 | 1B, 2D, 3B, 3D, 5A, 5B | *Vrn-A1* | (Mondal et al., 2015) |
| Fv /Fm (maximum quantum efﬁciency of photosystem II) | IPK- 2845 × IPK-970,  IPK-8183 × IPK-9705, IPK-28703 × IPK-9705 | F_2_ | 140 | 1D, 3B | *NAD(P)H-quinone oxidoreductase subunit 2B* (*ndhB2*), *photosystem I iron-sulfur centre* (*psaC*), *chloroplastic 3-isopropylmalate dehydrogenase 2* (*IMDH2*)*,* *beta-glucosidase 26* (*bglu26*), *fructokinase 2* (*frk2*) | (Sharma et al., 2017) |
| Chlorophyll content, plasma & thylakoid membrane damage | Ventnor × Karl 92 | RIL | 101 | 1B, 1D, 2B, 6A, 7A | *srg6, topoisomerase I, CDPK,* Aquaporins | (Talukder et al., 2014) |
| HSI of grain yield & components, grain filling rate & duration | HD2808 × HUW510 | RIL | 251 | 2A, 2B, 6D | - | (Bhusal et al., 2017) |
| Grain yield, Biomass, photosynthesis, TGW | Avalon × Cadenza; Multiple | DH, RIL | - | 1B, 2B, 2D, 4A, 4B, 4D, 5A, 7A | *Zeta1‐COP, SIT4 phosphatase, PGPD14, Raffinose synthase, Raffinose synthase, F-box* | (Acuña-Galindo et al., 2015) |
| Grain yield, grain filling duration, HSI | K 7903 × RAJ 4014 | RIL | 220 | 1B, 2B, 3B, 5A, 6B | - | (Sharma et al., 2016) |
| Chlorophyll ﬂuorescence kinetics parameters, F_v_ /F_m_ | Hanxuan 10 × Lumai 14 | DH | 150 | 1A, 1B, 2B, 4A, 7D | - | (Azam et al., 2014) |
| Grain yield, proline content, water soluble carbohydrates,  Fv /Fm, cytoplasmic membrane stability, chlorophyll content | SeriM82 × Babax | RIL | 167 | 2B, 2D, 4A, 4B, 5B, 6A | - | (Hassan et al., 2018) |
| TGW, grain filling rate, stay-green, canopy temperature, NDVI | SeriM82 × Babax | RIL | 167 | 1B, 2A, 2B, 4A, 4B, 7B, 7D | *SRG1* | (Pinto and Reynolds, 2015; Pinto et al., 2016) |
| GY, TGW, NDVI, days to heading | SeriM82 × Babax; SYN-D × Weebill 1 | RIL | 156;  276 | 1A, 1B, 2D, 3A, 3B, 4A, 4B, 4D, 5A, 5B, 5D, 6A, 6B, 6D, 7A, 7D | *Vrn-D4, Vrn-B3, Rht-B1, NST1-like protein, TPR15* | (Liu et al., 2019a, 2019b) |
| Grain yield, susceptibility indices, yield stability coefficient | GWAS |  | 720 | 1B, 2B, 3A, 3B, 4A, 4B, 5B, 6A, 6B, 7A, 7B, 7D | *Vrn*-*B1*, *Ppd-D1, Vrn-D3* | (Sehgal et al., 2017) |
| Grain yield in drought & heat stress | GWAS |  | 6,461 | 1A, 1B, 2B, 4A, 6B, 7B | *Vrn-B1* | (Sehgal et al., 2020a) |
| Grain yield, 1000-  grain weight, days to  heading, plant height,  grain filling duration | GWAS |  | 125 | 1B, 1D, 2A,  2B, 2D, 3B,  4B, 5A, 6A,  6B, 6D, 7A | Phenylalanine ammonia-lyase, helix-  loop-helix (bHLH) domain, F-box-like  domain, PTHR 44519, tansmembrane  helices, Pentatricopeptide repeat | (Jamil et al., 2019) |
| Cell membrane stability, grain yield | GWAS |  | 2,111 | 1A, 1B, 2A, 4A, 6B, 7B | *GDE1*, *TRAF-type zinc finger protein*, *SWI3B*, *ATPase, PRP38* | (ElBasyoni et al., 2017) |
| Grain yield, biomass, TGW, days to heading, PH | GWSA |  | 197 | 1A, 1B, 2A, 2B, 3B, 4A, 5A, 5B, 6B, 7A, 7B, 7D | *Vrn‐A1* | (Tadesse et al., 2019) |
| Spike ethylene | GWAS |  | 130 | 3B, 7B, 7A, 7B |  | (Valluru et al., 2017) |

*HSI; heat susceptibility index, HTI; heat tolerance index, Pop.; population, LGs; linkage groups

**Supplementary S4: salinity tolerance in bread wheat**

Although a large area of the world’s land is considered saline, research on salinity stress responses in crops is limited by the complexity of these responses and their interactions with other stresses [95]. Compared to drought tolerance, QTL mapping for salt tolerance (ST) is scarce and mainly focussed on sodium exclusion (NAX), K+ concentration (KC), and grain yield under salinity (Table 4). Before the release of the wheat draft sequence in 2014, QTL for grain yield, NAX, KC and shoot weight were focussed [96]. QTL studies for ions other than Na+ and K+ under salinity are rare. QTL for Cl- in wheat were found to differ between hydroponic and field conditions, and a major chromosome 5A QTL for Cl- contributed 27.0–32.0% to PV in the field. Additionally, 19 QTL for Mg2+ and Ca2+ were mapped at the same location as of Cl- QTL [97] with potential candidate genes, chloride channel (CLC) and cation chloride co-transporter (CCC).

Table S4. Examples of genomic regions and candidate genes for salinity tolerance in bread wheat

| **Traits studied** | **Method** | **Chromosomes** | **PV %** | **Candidate Genes** | **Ref.** |
| --- | --- | --- | --- | --- | --- |
| Sodium exclusion, K^+^, Tillers (n), total chlorophyll, seedling biomass | QTL | 1A, 1D, 2A, 2D, 3B, 3D, 4B, 4D, 5A, 5B, 5D, 6A, 6D, 7A, 7D | 10–18 | *HKT1;4* | (Genc et al., 2010) |
| Cl^-^, Ca^+2^, Mg^+2^ | QTL | 2A, 3A, 3B, 4A, 4D, 5A, 7D, | 27–32 | *Chloride channel (CLC), cation chloride co-transporter (CCC).* | (Genc et al., 2014) |
| Sodium exclusion, P, K^+^, Ca^+2^, Mg^+2^, S, Cu, Fe, Zn, B, Mg; root weight, shoot weight | QTL | 1A, 1D, 2A, 2B, 3B, 3D, 4B, 5A, 6A, 6B, 7A, 7B | 2–18.8 | *HKT1;4*, *K* *outward-rectifying channel (SKORs)*, K transporter 12 (*KUP12)*, *CLC-e, transparent testa 12, glutathione S-transferase GSTU6*, *peroxidase 12*, *auxin transport BIG*, *ARF5-like*, *ARF21*, *NAC78*, *NIPA4,* *Zn transporter 6* | (Hussain et al., 2017a) |
| Shoot growth, NAX, K^+^ | QTL | 2B, 5A, 6A, 7A | 4.9–29.8 | *Sodium/hydrogen exchanger 7* (*NHX7*), *potassium transporter 1* (*KUP1*), *KUP12*, *HKT2;1,* *HKT2;4, SKORs, TPK channel* , *H+ pyrophosphatase, H+-ATPase 4* | (Asif et al., 2018) |
| Shoot fresh/dry weight, seedling biomass, K^+^, Na^+^, chlorophyll content | GWAS | All | 2.4– 42.8 | *Transparent testa 12, chloroplast iron-superoxide dismutase, Serine/threonine protein kinase Nek6, ethylene responsive transcription factor ERF3, bHLH30, GDP-mannose transporter GONST1* | (Chaurasia et al., 2020) |
| Sodium exclusion, K^+^, shoot weight under salinity | GWAS | 1B, 1D, 2A, 3A | 3–30.7 | *ZIP7*, *KeFC, SAP8*, *HAK18, GST1,* *SWEET17* | (Oyiga et al., 2018) |
| Germination, salt tolerances indexes | GWAS | 1A, 3B, 6B | 2–8.9 | *Abscisic acid-insensitive 5-like (TaABI5-like), DUF674 family protein, SUMO-activating enzyme subunit 1A, Glutamate formiminotransferase 1, Protein KRI1, NADH-cytochrome b5 reductase* | (Yu et al., 2020) |
| Grain yield and related traits | GWAS | 1B, 3B, 4A, 4D, 5A, 5B, 7A | 9.1–68.8 | *Serine/threonine protein kinases, ricin B-like lectin gene, phytochelatin synthase, MADA-box genes, GPAT, U-box E3 ubiquitin ligase, lipid transfer proteins (LTPs)* | (Hu et al., 2021) |

GWAS = genome-wide association study, QTL = Quantitative trait loci, PV= Phenotypic variation

**Supplementary S5: Frost tolerance**

The C-repeat binding factor/dehydration responsive element binding factor (*CBF*/*DREB*) is a member of the *AP2/ERF* multi-gene family, which represent critical regulators of the freezing tolerance mechanisms in plants. These genes are regulated through inducers of CBF expression (*ICEs*) by binding to MYC recognition *cis*-elements (CANNTG) in the promoter (Jin et al., 2018), which constitutes the signal regulatory pathway, *ICE*–*CBF*–*COR*. This cascade mediates the response to cold stress. CORs (cold-responsive) are pointed to proteins that are encoded by cold-responsive or cold-regulated genes. These genes are induced by CBF or DREB2 proteins through binding to *cis*-acting dehydration responsive elements (DRE) or C-repeat (CRT) motif (5′-CCGAC- 3 (Guo et al., 2019). In addition to the role of *CBF* genes in cold response, vernalization (*VRN*) genes are responsible for natural differences in frost tolerance in wheat. Changes in the regulatory regions of vernalization genes (*VRN1* and *VRN3* genes or in the coding regions of *VRN2*) cause a delay in flowering time in plants. In summary, this body of research has provided useful resources to address frost tolerance breeding in wheat and genes/loci that could be incorporated in MAS and GS.

**Supplementary S6: Disease resistance**

Food crops, such as wheat, have always been adversely affected by diseases and pests. Genomic technologies available nowadays can be incorporated into wheat breeding program to complement traditional resistance breeding and meet these pressing challenges (Nelson et al., 2018). Modern breeding strategies are dealing with diverse forms of resistance, including both qualitative and quantitative types of resistance. Qualitative resistance tends to confer complete or near-complete resistance encoded by single resistance genes (R-genes) encoding immunoreceptors [nucleotide-binding leucine-rich repeat (NLR) proteins], also known as major genes (Nelson et al., 2018). Within a single host there may be multiple resistance genes (*R*-genes) for recognizing different races of a pathogen, and pyramiding these genes within elite varieties is vital for maintaining sustainable resistance (Hussain, 2015).

In contrast to the above example, an incomplete or partial phenotype shown by quantitative disease resistance (QDR) is regulated by multiple genes of small effect encoded by minor genes and map to quantitative trait loci (QTL) (Nelson et al., 2018). Generating polygenic resistance by incorporating multiple loci with small effect, into one cultivar, using classical breeding strategies can be even more challenging than transferring monogenic resistance (Nelson et al., 2018) . Transgenic or cisgenic approaches can provide a more realistic solution for wheat breeding with durable resistance, as demonstrated for the construction of a five-transgene cassette that confers broad-spectrum resistance to wheat stem rust (Luo et al., 2021).

**Leaf and stem rust**

In recent years, high throughput genotyping, QTL mapping and GWAS have commonly used approaches for the identification of resistance loci, *R*-genes and candidate genes due to their ability to identify significant SNPs controlling specific traits. For example, linkage maps constructed with genotyping data from a 90K SNP array for five doubled haploid populations mapped QTL for leaf rust resistance on chromosome 1A, 2A, 2B, 3B, 2D, 4B, 5A, 6A, 6B, 7A, 7B and 7D that were associated with *Lr3, Lr16, Lr17a, Lr23, Lr34/Yr18, Lr72* resistance genes (Bokore et al., 2020). Similarly, genotyping with 90K array identified two dominant alleles for conditioned resistance to the Ug99 race, also known as TTKSK. Furthermore, two major QTL were mapped on 2BL and 6DS for seedling and adult plant resistance to stem rust [104, 106]. The loci for recognition of two Ug99 races, BCCBC and TTKSK, were found to be located at the same location (*KASP_IWB1208* marker) on chromosome 2BL and was associated with *Sr28* (Babiker et al., 2017).

Recently, the 9K and 90K SNP genotyping arrays and GBS have been utilized by multiple groups for GWAS to dissect the genetic architecture of stem rust resistance (Mihalyov et al., 2017; Muleta et al., 2017; Yu et al., 2017; Edae et al., 2018; Mourad et al., 2018). For example, genotyping with the 9K array and GWAS identified 12 significant MTAs for resistance to the notorious wheat stem rust (Puccinia graminis f. sp. tritici (Pgt)) (Yu et al., 2017). Among them, SNPs found on chromosomes 4A and 4B were co-localized with SrND643, Sr37, and Ug99 resistance genes. Whereas 7DL SNPs coincided with Sr25 and other stem rust resistance genes. These SNPs were found to be located within genes annotated to be regulatory factors, plant disease resistance genes, or metabolic enzymes, six of which were validated to be Ug99 resistance genes by Kompetitive Allele-Specific PCR (KASP) assay. To add to this, the wheat 90K array was used to identify 22,310 significant high-quality SNP markers and several major significant MTAs for resistance against four races of stem rust (Puccinia graminis) that were on 1A, 2B, 3B, 2D, 4A that were co-localized with the Sr6, Sr7a, and Sr9b genes onto 1AL. Most of the genotypes possessed three or more Sr genes (Sr57, Sr12, Sr11, Sr9b, Sr8a, Sr7a, and Sr6) in various combinations (Edae et al., 2018). Another GWAS (Mihalyov et al., 2017) based on 1,411 wheat accessions and 5,390 SNPs, identified significant MTAs for resistance to four races of stem rust (TTTTF, BCCBC, TRTTF, TTKSK or Ug99) ten of which were associated with *Sr8a, Sr9h, Sr28, Sr31, Sr36, Sr39, Sr40, Sr47* including three novel loci. Furthermore, GBS data has been used to identify markers for disease resistance with GWAS e.g. a GBS data for 270 wheat accessions identified ∼35,000 high-quality SNPs, of which 32 were significant MTAs (GWAS) for stem rust resistance located mainly in close proximity to the *Sr6* gene on chromosome 2D (Mourad et al., 2018). Similarly, the wheat 90K array, SNPs and GWAS have been used to characterise disease resistance to stem and stripe rust in Ethiopian wheat bread lines (Muleta et al., 2017). Other candidate genes associated with MTAs were members of the *NLR* (nucleotide-binding domain leucine-rich repeat) gene family, nuclear monodehydroascorbate reductase 6 (*MDAR6*), solanesyl-diphosphate synthase 1 (DSDS1), enhancer of AG-4 protein 2 (*AG4*), phosphatase 2C (*PP2C*), and importin-9 (*IPT9*) and are listed in Table S5.

**Stripe (yellow) rust**

Another devastating wheat disease, Puccinia striiformis f. sp. tritici (Pst) fungus mediated stripe rust (or yellow rust) is responsible for significant yield losses worldwide. Sometimes, severe yield losses happen due to the pathogen attack in the early seedling stage and the progress in disease development during the season (Nemri et al., 2014). The use of major race-specific R-genes in wheat varieties is an effective and environmentally safe way for wheat disease management. Wheat genotyping assays and GBS has been utilized in recent years to map genomic regions associated with stripe rust resistance (Maccaferri et al., 2015; Yang et al., 2019) (Table S5).

**Powdery mildew**

Powdery mildew caused by the biotrophic fungus Blumeria graminis f. sp. tritici (Bgt) can cause 5%-62% of yield losses in susceptible varieties (Singh et al., 2016). More than 100 alleles in about 60 loci (officially designated *Pm1* - *Pm68*) have been mapped in wheat or its wild relatives (McIntosh et al.). Various studies have used GWAS to identify powdery mildew resistance loci in wheat (Liu et al., 2017b; Bhatta et al., 2019; Kang et al., 2020). Liu et al. (Liu et al., 2017b) mapped MTAs for powdery mildew in U.S. winter wheat on chromosomes 1A, 1B, 1D, 2A, 3B, 5A, 5B and 6A and the SNPs were tightly linked with Pm17, PmHNK, Pm24, Pm30 and Pm45 genes. Kang et al. (Kang et al., 2020) identified 33 MTAs for adult-plant resistance to powdery mildew on 1B, 2B, 2D, 3A, 3B, 5A, 5B, 6A, 6B and 7D, which were linked with candidate genes for peptidase family M3 and B56 subunit of protein phosphatase 2A (PP2A). Importantly, GBS-based GWAS identified seven, five, three and 12 MTAs for powdery mildew, stem rust, septoria and leaf rust resistance on all chromosomes (except for 4B and 5D) (Bhatta et al., 2019). The MTAs were associated with several candidate genes for leaf rust (GDSL esterase/lipase, Vesicle-associated 1-1-like protein, E3 Ubiquitin ligase family protein, Phosphatidic acid phosphatase, 12-oxophytodienoate reductase-like protein), Septoria (F-box/RNI-like/FBD-like domains-containing protein) and stem rust (Zinc transporter, putative).

The low number of functional molecular markers is limiting the diagnosis of the potential Pm alleles and their deployment in wheat breeding via MAS and genome editing (Table S5).

**Blotches and Fusarium head blight**

Furthermore, GWAS has applied as well for dissecting the genetic bases for various diseases. For example, a GBS of 273 wheat accessions identified 19,992 SNPs, and ten significant MTAs for Fusarium head blight (Fhb) on chromosomes 1D, 3B, 4A, 4D, 6A, 7A, and 7D were mapped (Arruda et al., 2016). The combination of these favorable alleles in genotypes caused reduced deoxynivalenol concentration, incidence, and severity of the disease, while several favorable SNPs were linked to the previously map-based cloned Fhb1 gene on chromosome 3B (Arruda et al., 2016). Similarly, genomic regions for black point reaction (Liu et al., 2017a) were associated with candidate genes for F-box repeat, Polyphenol oxidase (PPO-A1), RPP8-like, Serine/threonine-protein kinase, Peroxisomal biogenesis factor 2 (PEX2). Besides, a wheat 9K SNP array was used for genotyping and a subsequent GWAS identified significant MTAs for resistance to bacterial leaf streak (caused by Xanthomonas translucens), leaf spot blotch (caused by *Cochliobolus sativus*) and *Stagonospora nodorum* blotch diseases and stripe rust, respectively (Gurung et al., 2014; Kollers et al., 2014; Maccaferri et al., 2015; Corsi et al., 2020) including both novel and known genes of resistance as further detailed in Table S5.

**Wheat Viruses**

Soil-borne yellow mosaic-inducing virus diseases (Fig.1) seriously threaten the global production of autumn-sowing wheat. The diseases are caused by infection of soil-borne plasmodiophorid Polymyxa graminis L. under natural field conditions. The yellow mosaic-inducing virus diseases are infected by the bipartite viruses belonging to two genera, the Bymovirus of the Potyviridae and the Furovirus of the Virgaviridae, and usually cause mixed infections (Jiang et al., 2020). Due to transmission by the P. graminis, which has been detected down to a soil depth of 60 cm, chemical measures are neither effective nor acceptable for economic and ecological reasons; therefore, the only possibility of controlling these viruses is through breeding resistant or tolerant cultivars. Soil-borne wheat mosaic virus (SBWMV) and Soil-borne cereal mosaic virus (SBCMV) as well as Wheat spindle streak mosaic virus (WSSMV) belonging to the soil-borne Furoviruses or Bymoviruses, respectively, are serious constraints to winter wheat cultivation in Europe and North America (Clover et al., 2001; Jiang et al., 2020) while in Asia, Bymovirus Wheat Yellow Mosaic Virus (WYMV), are major constraints to winter wheat cultivation. In bread wheat, the genetic analysis of Furovirus resistance has found one major locus: Sbm1, on chromosome 5D and was shown to be effective against SBCMV in Europe (Bass et al., 2006). The same major QTL QSbm.uga-5DL was identified in all environments with very significant LOD values, explaining up to 62 and 65 % of the total variation. This locus QSbm.uga-5DL coincided with previously reported SBCMV resistance genes *Sbm1, SbmClaire* and *SbmTrémie* on the long arm of chromosome 5D (Perovic et al., 2009). Additionally, two major WYMV resistance QTL, *Qym1* and *Qym2* were mapped on wheat chromosome 2D (Suzuki et al., 2015) (Table S5).

Two independent GWAS analyses utilizing iSelect 9K and 90K Illumina arrays have reported SNPs and genes for SBWMV resistance (Liu et al., 2014, 2020). Liu et al. (Liu et al., 2020) completed a GWAS analysis of SBWMV resistance using the 90K Illumina array. Thirty-five SNPs in 12 wheat genes and one intergenic SNP in the Sbwm1 region were identified on chromosome 5D that was associated significantly with SBWMV resistance. Resistance to SBWMV was strongly associated with putative kinase family protein (Liu et al., 2014). Furthermore, GWAS analysis identified major resistance SNPs for WSSMV on chromosome 2D in addition to regions on 5B and 7D. The 2D genomic region was linked with 18 candidate genes including eleven NBS‐LRR genes (Hourcade et al., 2019), and are listed in Table S5.

Table S5. Examples of genomic regions, candidate and cloned genes mapped for disease resistance in wheat species

| **Disease Resistance** | **Method** | **Chromosome** | **PV (%)** | **Cloned/candidate genes** | **Ref.** |
| --- | --- | --- | --- | --- | --- |
| Leaf rust (*Puccinia triticina*) | QTL | 1A, 2A, 2B, 3B, 2D, 4B, 5A, 6A, 6B, 7A, 7B, 7D | 1.9–24 | *Lr3, Lr16,* *Lr17a, Lr23,* Lr34/Yr18/Pm38, *Lr72* | (Bokore et al., 2020) |
| Leaf rust | Cloned | 1D |  | *Lr21* | (Huang et al., 2003) |
| Stem rust (*Puccinia graminis*) v. Ug99 | QTL | 2B, 6D | 16.3–34.8 | *Sr28* | (Babiker et al., 2017) |
| Stem rust | GWAS | 4A, 4B, 7B, 7D | – | *SrND643*, *Sr25,* *Sr37*, *NBS-LRR*, *IPT9,* *RPP13*, *RGA3*, *MDAR6,* *DSDS1*, *PP2C*, *AG4* | (Yu et al., 2017) |
| Stem rust | GWAS | 1A, 2B, 3B, 2D, 4A | 12.31–16.94 | *Sr6*, *Sr7a,* *Sr8a*, *Sr9b, Sr11*, *Sr12*, *Sr57*, | (Edae et al., 2018) |
| Stem rust | GWAS | 1AL, 1B, 2A, 2B, 2D, 6A | 33.9– 39.8 | *Sr8a*, *Sr9h*, *Sr28*, *Sr31, Sr36, Sr39, Sr40, Sr47* | (Mihalyov et al., 2017) |
| Stem rust | GWAS | 2D | 11.7– 17.68 | *Sr6* | (Mourad et al., 2018) |
| Stem rust | Cloned | 7D |  | *SuSr-D1* | (Hiebert et al., 2020) |
| Stem rust | Cloned | 7A |  | *Sr22* | (Steuernagel et al., 2016) |
| Stem rust | Cloned | 1D |  | *Sr33* | (Periyannan et al., 2013) |
| Stem rust | Cloned | 3A |  | *Sr35* | (Saintenac et al., 2013) |
| Stem rust | Cloned | 1D |  | *Sr45* | (Steuernagel et al., 2016) |
| Stem rust | Cloned | 2D |  | *Sr46* | (Arora et al., 2019) |
| Stem rust | Cloned | 5A |  | *Sr60* | (Chen et al., 2020) |
| Powdery mildew (*Blumeria graminis* f. sp. *tritici*) | GWAS | 1A, 1B, 1D, 2A, 3B, 5A, 5B, 6A | – | *Pm17, PmHNK, Pm24, Pm30*, *Pm45* | (Liu et al., 2017b) |
| Powdery mildew | GWAS | 1B, 2B, 2D, 3A, 3B, 5A, 5B, 6A, 6B, 7D | – | *Peptidase family M3, B56 subunit of protein phosphatase 2A (PP2A)* | (Kang et al., 2020) |
| Powdery mildew, stem rust, Septoria, leaf rust | GWAS | All except for 4B and 5D | – | *Zinc transporter, putative, F-box/RNI-like/FBD-like domains-containing protein, Phosphatidic acid phosphatase, OPR2, E3 Ubiquitin ligase, GDSL esterase* | (Bhatta et al., 2019) |
| Powdery mildew | MutRenSeq | 7A | - | *Pm1a* | (Xing et al., 2018) |
| Powdery mildew | MutChrom Seq | 5D | - | *Pm2* | (Sánchez-Martín et al., 2016) |
| Powdery mildew | Homology-based cloning | 1A | - | *Pm3* | (Hurni et al., 2013) |
| Powdery mildew | MBC | 1A |  | *Pm3b* | (Yahiaoui et al., 2004) |
| Powdery mildew | MBC | 7B |  | *Pm5e* | (Xie et al., 2020) |
| Powdery Mildew | MBC | T6VS·6AL |  | *Pm21* | (Xing et al., 2018) |
| Powdery Mildew | MBC | 1D |  | *Pm24* | (Lu et al., 2020) |
| Powdery mildew | MBC | 7D |  | *Pm38/Lr34/Yr18/Sr57* | (Krattinger et al., 2009) |
| Powdery mildew | MBC | 3B |  | *Pm41* | (Li et al., 2020) |
| Powdery mildew | MBC | 4D |  | *Pm46/Lr67/Yr46/Sr55* | (Moore et al., 2015) |
| Powdery mildew | MBC | 7A |  | *Pm60* | (Zou et al., 2018) |
| Fusarium head blight | GWAS | 1D, 3B, 4A, 4D, 6A, 7A, 7D | 4–16 | *Fhb1, Rht‐B1, Rht‐D1* | (Arruda et al., 2016) |
| Fusarium head blight | Cloned | 3B |  | *Fhb1* | (Su et al., 2019) |
| Fusarium head blight | Cloned | 7E |  | *Fhb7* | (Wang et al., 2020a) |
| Black point reaction disease | GWAS | 2A, 2B, 3A, 3B, 3D, 4B, 5A, 5B, 6A, 6B, 6D, 7A, 7B, 7D | 7.9–18.0 | *Polyphenol oxidase (PPO-A1*), *Peroxisomal biogenesis factor 2* (*PEX2*), *RPP8-like,* F-box repeat | (Liu et al., 2017a) |
| Leaf spot blotch + bacterial leaf streak + *Parastagonospora nodorum* blotch | GWAS | 1A, 1B, 2A, 2B, 2D, 3A, 3B, 4B, 4D, 5A, 5B, 5D, 6A, 6B, 6D, 7A, 7B, 7D | 1.9– 14.3 | *Tsn1, Tsn2, Tsn5, Tsc2, Tsr6, PAH1, ACLA-3, ACA8, MAK10, MRP5, ESP4, OXS3, SAG12, SYTA, SYTB, CCB1* | (Gurung et al., 2014; Kollers et al., 2014; Corsi et al., 2020) |
| Stripe/Yellow rust (*Puccina striiformis* f. sp. *tritici*) | GWAS | 1B, 1D, 2A, 2B, 3B, 4A, 4D, 5A, 6B, 6D | 15–45 | *Lr34/Yr18/Pm38, Lr67/Yr46* | (Maccaferri et al., 2015) |
| Stripe rust | QTL | 1A, 2A, 2B, 4A, 4B, 6B, 7D | 9.7–26.8 | *Yr33* | (Yang et al., 2019) |
| Stripe rust | Cloned | 6B |  | *Yr36* | (Fu et al., 2009) |
| Stripe rust | Cloned | 1B |  | *Yr15* | (Klymiuk et al., 2018) |
| Stripe rust | Cloned | 5A |  | *YrU1* | (Wang et al., 2020b) |
| Stripe rust | Cloned | 2B |  | *Yr5, Yr7*, *YrSP* | (Marchal et al., 2018) |
| Tan spot; *Stagonospora nodorum* blotch | Cloned | 5B |  | *Tsn1* | (Faris et al., 2010) |
| *Parastagonospora nodorum* blotch | Cloned | 1B |  | *Snn1* | (Shi et al., 2016) |
| *Septoria tritici* blotch (STB) | Cloned | 3A |  | *Stb6* | (Saintenac et al., 2018) |
| *Septoria tritici* blotch | Cloned | 3D |  | *Stb6q* | (Saintenac et al., 2021) |
| Soil-borne wheat mosaic virus (SBWMV) | GWAS, QTL | 5D | – | *Putative kinase family protein* | (Liu et al., 2014) |
| Soil-borne wheat mosaic virus | GWAS | 5D | – | *Sister chromatid cohesion protein, kinase family protein, pto-interacting protein 1 (PTI1), pollen allergen-like protein, serine/threonine phosphatase, KDEL sequence binding protein, K channel activity protein, PARG* | (Liu et al., 2020) |
| Soil-borne cereal mosaic virus (SBCMV) | QTL | 5D | – | – | (Bass et al., 2006; Perovic et al., 2009) |
| Wheat Yellow Mosaic Virus (WYMV) | QTL | 2D, 4B, 4D | – | – | (Suzuki et al., 2015) |
| Wheat spindle streak mosaic virus (WSSMV) | GWAS | 2A, 2B, 2D, 3B, 5B, 5D, 7A, 7B, 7D | – | *NBS‐LRR genes* (11)*, dicer‐like proteins* (2)*, RNA‐dependent RNA polymerase protein, RGA2, RPM1* | (Hourcade et al., 2019) |

GWAS = genome-wide association study. QTL = quantitative trait loci. PV = phenotypic variation

**Supplementary S7 insect and mite resistance**

There are a number of arthropod pests that can cause major yield losses in wheat. Efforts to identify resistance genes to insects lag behind those for other diseases, but with increased awareness and demand for sustainable wheat production methods, this field is accelerating in recent years. The insects discussed here such as the Russian wheat aphid (Diuraphis noxia), the greenbug (Schizaphis graminum) and the Hessian fly (Mayetiola destructor) where single dominant resistance genes have been found to confer resistance, whereas for the bird cherry-oat aphid (Rhopalosiphum padi) and the English grain aphid (Sitobion avenae) where good resistance has been harder to identify and seems to be under the control of multiple genes. Methods for phenotyping for insect resistance vary depending on the type of insect, clear visual symptoms, such as for D. noxia, S. graminum and M. destructor are associated with a larger number of genes identified, whereas those that cause damage to yield and quality without leaving visual symptoms and where phenotyping is more difficult like R. padi and S. avenae have fewer resistance sources identified (Aradottir and Crespo-Herrera, 2021). As noted below, named BYDV resistance genes have been assigned in wheat.

Hessian fly (*Mayetiola destructor*) is a major pest in wheat in the USA which has been successfully controlled using singly deployed H genes for over 50 years; 100% of *M. destructor* larvae die before causing damage in resistant germplasm, which is biotype dependant. Over 35 H genes have been identified, but none of those has been cloned (Anderson et al., 2019).

For the Russian wheat aphid (D. noxia), Dn resistance protects yield by maintaining chlorophyll functionality, whereas susceptible plants react to infestation with chlorotic streaks, leaf rolling and stunting. A number of *Dn* genes are reported; *Dn1, Dn2, Dn4, Dn5, Dn6, Dn8* and *Dn9* are thought to be dominant and derive from *T. aestivum*, whereas the recessive dn3 derives from *Ae. tauschii*, and Dn7 came for a rye translocation (Tolmay et al., 2020). A recent review suggests however that considering the location of the majority of the Dn genes near the centromere of 7D and the difficulty in identification of diagnostic markers for the genes, the resistance may be instead be controlled by closely linked genes or QTL inﬂuenced by the genetic background they occur in (Tolmay et al., 2020). The deployment of resistance genes needs to take biotype development in *D. noxia* into account, five biotypes are known from the USA and four from S-Africa (Harris, M O; Anderson, K; El-Bouhssini, M; Peairs, F; Hein, G; Xu, 2017).

The greenbug (*S. graminum*) is a serious aphid pest that occurs in all major wheat growing regions apart from Australia. It causes necrotic lesions on plants, is a vector for the barley yellow dwarf virus and causes indirect damage by acting as a winged transport mechanism for the wheat curl mite (Aceria tosichella) that vectors the wheat streak mosaic virus. The development of the first resistant wheat cultivars started in the 1950’s, and now there are >15 resistance genes reported. A number of greenbug biotypes have been identified whose distribution needs to inform the deployment of the resistance genes (Harris, M O; Anderson, K; El-Bouhssini, M; Peairs, F; Hein, G; Xu, 2017; Xu et al., 2020). Both genomic regions and candidate genes identified for the resistance against different classes of wheat pest are detailed in Table S6. Wheat resistance to the barley yellow dwarf virus vectoring bird cherry-oat aphid (R. padi) and the English grain aphid (S. avenae) has been elusive, as effective high throughput phenotyping methods have been lacking for these species, but resistance has been identified in wheat wild relatives from the primary and secondary gene pool, more commonly in species with low ploidy levels such as *Triticum boeoticum, Aegilops tauschii. Triticum araraticum, T. araraticum* and *Triticum dicoccoides*.

Many other insect pests are of importance in wheat production. The resistance gene *Sm1*, which acts against the orange wheat blossom midge (*Sitodiplosis mosellana*) has been deployed in Canada and Europe and four resistance genes (*Cmc1-4*) have been identified against the aforementioned wheat curl mite (*A. tosichella*) (Khalaf et al., 2019). Other pests that are of concern to wheat production include the Sunn pest (*Eurygaster integriceps*), wheat stem sawfly (*Cephus cinctus*) (WSS) and the yellow wheat blossom midge (*Contarinia tritici*). Hence there is no shortage of projects for insect resistance breeding programs in future, which will need to focus both on the discovery of new resistance traits and the maintenance of previously developed insect resistant genotypes (Smith, 2021). For instance, the use of solid stemmed cultivars has been the primary strategy against WSS until recently. Advances in the availability of genomics tools and resources first permitted a comparative analysis of this QTL in wheat and related grasses and additionally provided a global view of the plant response upon WSS manifestation at the transcriptome, proteome and metabolome levels (Biyiklioglu et al., 2018). Another study explored the WSS transcriptome and its interaction with the regulatory elements, microRNAs (miRNAs) and long non-coding RNAs (lncRNAs). Interestingly, this study found that WSS miRNAs may target wheat transcripts and vice versa, thereby potentially modulating the plant responses against WSS (Cagirici et al., 2017).

Table S6. Examples of genomic regions and candidate genes mapped for insect and mite resistance

| **Insect Resistance** | **Method** | **Chromosome** | **PV (%)** | **Candidate genes** | **Ref.** |
| --- | --- | --- | --- | --- | --- |
| Russian wheat aphid (*Diuraphis noxia*) | QTL, Physical Map | 1D, 7B, 7D, | – | *Dn1, Dn2, Dn3, Dn4, Dn5, Dn6, Dn7, Dn8, Dn9, Dn10, Dnx, Dny, Dn1881, Dn2414, Dn626580* | (Li et al., 2018; Tolmay et al., 2020) |
| Russian wheat aphid (*Diuraphis noxia*) | QTL | 1D, 7D | – | *Dn2401* | (Fazel-Najafabadi et al., 2015) |
| Wheat curl mite  (*Aceria tosichella*) | QTL Introgression | 6D | – | *Cmc1, Cmc2, Cmc4* | (Khalaf et al., 2019) |
| Wheat stem sawfly (*Cephus cinctus* Norton) | QTL | 3B | – | *SSt1, CPR-5* | (Biyiklioglu et al., 2018) |
| Greenbug  *(Schizaphis graminum) or wheat aphid* | LM | 7D | – | *Gb8* | (Xu et al., 2020) |
| Greenbug  *(Schizaphis graminum) or wheat aphid* | QTL | 2D, 7D | 10.2–16.6 | *Gba,* | (Crespo-Herrera et al., 2014) |
| Greenbug  *(Schizaphis graminum) or wheat aphid* | LM | 7D | – | *Gb7/* *Gbx2* | (Tan et al., 2017) |
| Greenbug  *(Schizaphis graminum) or wheat aphid* | QTL | 2B, 3A, 7B, 7D | *3,9*–*24* | *Gba, Gbb, Gbd, GbSkll* | (Crespo-Herrera et al., 2019) |
| Bird cherry-oat aphid *(Rhopalosiphum padi)* | QTL | 3A, 4B, 5A, 5B | 5.7–*35* | *Hl1, Tbx,* | (Crespo-Herrera et al., 2014) |
| Hessian fly (*Mayetola distructor*) | LM | 3D | – | *H32* | (Tan et al., 2017) |
| Hessian fly (*Mayetola distructor*) | QTL | 3B, 7A | *23.8*–*36* | *H35, H36, defensin-like protein.,cellulose synthase family protein, cellulose synthase-like protein, isoflavone reductase-like proteins, pectinesterase inhibitors, proteinase inhibitor* | (Zhao et al., 2020) |
| Orange wheat blossom midge (*Sitodiplosis mosellana*) | LM | 2B | – | *Sm1, sresponse protein NST1-like, E3 ubiquitin ligase, disease resistance protein RGA, Nicotiana lesion-inducing like, cyclopropane-fatty-acyl-phospholipid synthase* | (Kassa et al., 2016) |

**Supplementary S8: Quality traits**

In contrast to agronomic traits, dissection of quality traits in wheat by GWAS has received less attention. However, in the past two years, a few studies have reported quality traits dissection by association mapping approaches. These studies used high density SNP arrays and identified candidate genes (Table S7) in some cases (Nedelkou et al., 2017; Bhatta et al., 2019; Chen et al., 2019; Yang et al., 2020a). Chen et al. (Chen et al., 2019), for example, used the 90K genotyping assay to identify MTAs for grain hardness, GPC, WGC and flour color and reported 103multi-environment-signiﬁcant SNPs in more than four environments. Further, they reported that a disease resistance RPP13- like protein 1 gene, *TaRPP13L1*, was associated with ﬂour color. Wheat lines with *TaRPP13L1-B1a* showed signiﬁcantly higher ﬂour redness than those with TaRPP13L1-B1b in Chinese wheat. Other candidate genes included *TaRPP13L1-7B, TaRPP13L1-7D, MCM3, Pinb, SBE1, Psy, RPL1, SPY* and *STK*. Similarly, Bhatta et al. (Bhatta et al., 2019) explored synthetic and bread wheat accessions from Western Siberia for GPC and agronomic traits by GWAS using 192,876 GBS-SNPs, and based on MTA on chromosome 2D, identified two candidate genes (*TraesCS2D01G582500.1* and *TraesCS2D01G264700.1*) based on MTAs identified on chromosome 2D for GPC. Gene model *TraesCS2D01G582500.1* has a putative kinase function while *TraesCS2D01G264700.1* was annotated as a member of the NBS-LRR disease resistance proteins (NLRs) family. Yang et al. (Yang et al., 2020a) conducted a high-resolution multilocus-GWAS combined with gene network analysis of grain quality and dough rheological traits based on 19,254 SNPs genotyped in 267 bread wheat accessions. In that study, sixty-seven core candidate genes involved in protein/sugar synthesis, histone modiﬁcation and the regulation of transcription factor were reported to be associated with the grain quality. Furthermore, another GWAS study (Nedelkou et al., 2017) identified MTAs for grain hardness, GPC and flour sedimentation on chromosomes 3A, 3D, 4A, 4B, 4D, 5B, 5D, 6B and 7B that were associated with candidate genes *NAM-B1* and *Pinb-D1*. Furthermore, eight SNP markers for early identification of high molecular weight glutenin subunits (HMW-GSs) were reported (Ravel et al., 2020) that can be utilized in applied breeding. It is important to note that although immense efforts were invested in mapping and targeting genes for grain quality, only one gene, *NAM-B1* (*Gpc-B1*) (Uauy et al., 2006) has been map-based cloned.

Table S7. Examples of genomic regions, candidate and cloned genes for quality traits

| **Trait(s)*** | **Cross** | **Pop. Type** | **Pop. size** | **Chromosomes** | **Candidate/cloned genes** | **Ref.** |
| --- | --- | --- | --- | --- | --- | --- |
| GPC, GMPC, WGC, DGC, ZSV, FWA, MTI, FPV | Chuan 35050 × Shannong 483 | RIL | 131 | 1D, 2A, 2D, 3B, 3D, 5A, 6A, 6B, 6D, 7B | - | (Sun et al., 2008) |
| GPC, milling yield, FPC, FC, FWA, DDT, DS, DE | Chara × WW2449 | DH | 190 | 1B, 4A | *Glutenin Glu-B1* | (Raman et al., 2009) |
| Milling, dough rheology, bread making, FY | BR34 × Grandin | RIL | 118 | 1B, 1D, 4B, 5B, 6A | *Glu-D1* | (Simons et al., 2012) |
| GPC, FY, FPC, SRC, flour ash Grain hardness | Louise × Penawawa | RIL | 188 | 1A, 2A, 2B, 2D, 3B, 4A, 4D, 5B, 6A, 6B, 6D, 7D | *Wx-B1, Glu-B1, Glu-D1* | (Carter et al., 2012) |
| GPC, DDT, DST, FWA, SV, FN, PV | Tainong 18 × Linmai 6 | RIL | 184 | 1A, 1B, 1D, 4B, 5D, 6A, 6B, 6D, 7A, 7D | *EMF1, TPS, NRT1:2, ZNF830, phospholipid-transporting ATPase, transcription factor TFIIIB, APEH, F-box protein SKIP22, ALDH* | (Guo et al., 2020) |
| GPC, FPC, FWA, FY | Butte86 × ND260 | RIL | 132 | 1B, 2D, 7A, 7B | *Glu-A1, Glu-B1, Glu-A3, Glu-B3, Glu-D3* | (Boehm et al., 2017) |
| Dough rheological and starch pasting properties | Gaocheng 8901 × Zhoumai 16 | RIL | 176 | All except 4D | *AnPRT, 3-ketoacyl-CoA, ornithine aminotransferase, LOX2, SPS2, lysosomal beta glucosidase, mtnN* | (Jin et al., 2016) |
| GPC, dough viscosity, dough strength, dough volume, total starch | mQTL | RIL, DH, F_2_ | - | 1A, 1B, 2A, 2B, 2D, 3A, 3D, 4A, 4B, 6B, 7A | *GliA3, RuBisCO, Vivip-1, Glu-A1* | (Quraishi et al., 2017) |
| GPC, grain gluten content | GWAS | - | 143 | 1A, 1D, 2A, 2D, 3A, 3B, 4A, 5A, 5B, 7D | *Kinase, putative, NBS-LRR disease resistance protein* | (Bhatta et al., 2019) |
| Grain quality and dough rheological traits | GWAS | - | 267 | All | *Glucose-6-phosphate isomerase, Glutathione S-transferase, SWEET*  *Glycogen synthase, Histone H2A, H2B, H3, H4; Defensin, Starch synthase, E3 ubiquitin-protein ligase, glutenin subunits* | (Yang et al., 2020a) |
| FC, GPC, WGC, grain hardness | GWAS | - | 846 | 1B, 1D, 2A, 3A, 4B, 5A, 5B, 5D, 6A, 7A, 7D | *TaRPP13L1-7B, TaRPP13L1-7D, MCM3, Pinb, SBE1, Psy, RPL1, SPY, STK* | (Chen et al., 2019) |
| Grain hardness, GPC, flour sedimentation | GWAS | - | 359 | 3A, 3D, 4A, 4B, 4D, 5B, 5D, 6B, 7B | *NAM-B1, Pinb-D1* | (Nedelkou et al., 2017) |
| GPC, grain zinc and iron | Cloned | RIL | - | 6B | *Gpc-B1* | (Uauy et al., 2006) |

*GPC; grain protein content, FPC; flour protein content, GMPC; grain glutenin macropolymer content, WGC; wet gluten content, DGC; dry gluten content, ZSV; Zeleny sedimentation volume, FWA; flour water absorption, DDT; dough development time, MTI; mixing tolerance index, FPV; flour paste viscosity, FC; flour color, DS; dough strength, DE; dough extensibility, FY; Flour yield, SE; softness equivalent, AWRC; alkaline water retention capacity, SRC; solvent retention capacity, SV; sedimentation volume, BDT; break down time, DST; dough stability time, FN; falling number, PV; peak viscosity, TV; trough viscosity, PTi; peak time, Pte; pasting temperature

**References**

Acuña-Galindo, M. A., Mason, R. E., Subramanian, N. K., and Hays, D. B. (2015). Meta-analysis of wheat QTL regions associated with adaptation to drought and heat stress. *Crop Sci.* 55, 477–492. doi:10.2135/cropsci2013.11.0793.

Addison, C. K., Mason, R. E., Brown-Guedira, G., Guedira, M., Hao, Y., Miller, R. G., et al. (2016). QTL and major genes influencing grain yield potential in soft red winter wheat adapted to the southern United States. *Euphytica* 209, 665–677. doi:10.1007/s10681-016-1650-1.

Alqudah, A. M., Haile, J. K., Alomari, D. Z., Pozniak, C. J., Kobiljski, B., and Börner, A. (2020). Genome-wide and SNP network analyses reveal genetic control of spikelet sterility and yield-related traits in wheat. *Sci. Rep.* 10, 1–12. doi:10.1038/s41598-020-59004-4.

Anderson, K. M., Harris, M. O., and Peairs, F. (2019). Susceptibility of North Dakota Hessian Fly (Diptera: Cecidomyiidae) to 31 H Genes Mediating Wheat Resistance. *J. Econ. Entomol.* 112, 2398–2406. doi:10.1093/jee/toz121.

Appels, R., Eversole, K., Feuillet, C., Keller, B., Rogers, J., Stein, N., et al. (2018). Shifting the limits in wheat research and breeding using a fully annotated reference genome. *Science (80-. ).* 361. doi:10.1126/science.aar7191.

Aradottir, G. I., and Crespo-Herrera, L. (2021). Host plant resistance in wheat to barley yellow dwarf viruses and their aphid vectors: a review. *Curr. Opin. Insect Sci.* 45, 59–68. doi:10.1016/j.cois.2021.01.002.

Arora, S., Steuernagel, B., Gaurav, K., Chandramohan, S., Long, Y., Matny, O., et al. (2019). Resistance gene cloning from a wild crop relative by sequence capture and association genetics. *Nat. Biotechnol.* 37, 139–143. doi:10.1038/s41587-018-0007-9.

Arruda, M. P., Brown, P., Brown-Guedira, G., Krill, A. M., Thurber, C., Merrill, K. R., et al. (2016). Genome-Wide Association Mapping of Fusarium Head Blight Resistance in Wheat using Genotyping-by-Sequencing. *Plant Genome* 9, 0. doi:10.3835/plantgenome2015.04.0028.

Asif, M. A., Schilling, R. K., Tilbrook, J., Brien, C., Dowling, K., Rabie, H., et al. (2018). Mapping of novel salt tolerance QTL in an Excalibur × Kukri doubled haploid wheat population. *Theor. Appl. Genet.* 131, 2179–2196. doi:10.1007/s00122-018-3146-y.

Assanga, S. O., Fuentealba, M., Zhang, G., Tan, C. T., Dhakal, S., Rudd, J. C., et al. (2017). Mapping of quantitative trait loci for grain yield and its components in a US popular winter wheat TAM 111 using 90K SNPs. *PLoS One* 12, e0189669. doi:10.1371/journal.pone.0189669.

Asseng, S., Ewert, F., Martre, P., Rötter, R. P., Lobell, D. B., Cammarano, D., et al. (2015). Rising temperatures reduce global wheat production. *Nat. Clim. Chang.* 5, 143–147. doi:10.1038/nclimate2470.

Azam, F. i., Chang, X., and Jing, R. (2014). Mapping QTL for chlorophyll fluorescence kinetics parameters at seedling stage as indicators of heat tolerance in wheat. *Euphytica* 202, 245–258. doi:10.1007/s10681-014-1283-1.

Babiker, E. M., Gordon, T. C., Chao, S., Rouse, M. N., Wanyera, R., Acevedo, M., et al. (2017). Molecular Mapping of Stem Rust Resistance Loci Effective Against the Ug99 Race Group of the Stem Rust Pathogen and Validation of a Single Nucleotide Polymorphism Marker Linked to Stem Rust Resistance Gene Sr28. *Phytopathology* 107, 208–215. doi:10.1094/PHYTO-08-16-0294-R.

Ballesta, P., Mora, F., and Del Pozo, A. (2020). Association mapping of drought tolerance indices in wheat: QTL-rich regions on chromosome 4A. *Sci. Agric.* 77, 2020. doi:10.1590/1678-992x-2018-0153.

Bass, C., Hendley, R., Adams, M. J., Hammond-Kosack, K. E., and Kanyuka, K. (2006). The Sbm1 locus conferring resistance to Soil-borne cereal mosaic virus maps to a gene-rich region on 5DL in wheat. *Genome* 49, 1140–1148. doi:10.1139/G06-064.

Bennett, D., Reynolds, M., Mullan, D., Izanloo, A., Kuchel, H., Langridge, P., et al. (2012). Detection of two major grain yield QTL in bread wheat (Triticum aestivum L.) under heat, drought and high yield potential environments. *Theor. Appl. Genet.* 125, 1473–1485. doi:10.1007/s00122-012-1927-2.

Bhatta, M., Shamanin, V., Shepelev, S., Stephen Baenziger, P., Pozherukova, V., Pototskaya, I., et al. (2019). Marker-trait associations for enhancing agronomic performance, disease resistance, and grain quality in synthetic and bread wheat accessions in Western Siberia. *G3 Genes, Genomes, Genet.* 9, 4209–4222. doi:10.1534/g3.119.400811.

Bhusal, N., Sarial, A. K., Sharma, P., and Sareen, S. (2017). Mapping QTLs for grain yield components in wheat under heat stress. *PLoS One* 12, e0189594. doi:10.1371/journal.pone.0189594.

Biyiklioglu, S., Alptekin, B., Akpinar, B. A., Varella, A. C., Hofland, M. L., Weaver, D. K., et al. (2018). A large-scale multiomics analysis of wheat stem solidness and the wheat stem sawfly feeding response, and syntenic associations in barley, Brachypodium, and rice. *Funct. Integr. Genomics* 18, 241–259. doi:10.1007/s10142-017-0585-5.

Boehm, J. D., Ibba, M. I., Kiszonas, A. M., See, D. R., Skinner, D. Z., and Morris, C. F. (2017). Identification of genotyping-by-sequencing sequence tags associated with milling performance and end-use quality traits in hard red spring wheat (Triticum aestivum L.). *J. Cereal Sci.* 77, 73–83. doi:10.1016/j.jcs.2017.07.007.

Bokore, F. E., Knox, R. E., Cuthbert, R. D., Pozniak, C. J., McCallum, B. D., N’Diaye, A., et al. (2020). Mapping quantitative trait loci associated with leaf rust resistance in five spring wheat populations using single nucleotide polymorphism markers. *PLoS One* 15, e0230855. doi:10.1371/journal.pone.0230855.

Budak, H., Hussain, B., Khan, Z., Ozturk, N. Z., and Ullah, N. (2015). From genetics to functional genomics: Improvement in drought signaling and tolerance in wheat. *Front. Plant Sci.* 6, 1–13. doi:10.3389/fpls.2015.01012.

Cagirici, H. B., Biyiklioglu, S., and Budak, H. (2017). Assembly and annotation of transcriptome provided evidence of miRNA mobility between wheat and wheat stem sawfly. *Front. Plant Sci.* 8, 1653. doi:10.3389/fpls.2017.01653.

Carter, A. H., Garland-Campbell, K., Morris, C. F., and Kidwell, K. K. (2012). Chromosomes 3B and 4D are associated with several milling and baking quality traits in a soft white spring wheat (Triticum aestivum L.) population. *Theor. Appl. Genet.* 124, 1079–1096. doi:10.1007/s00122-011-1770-x.

Chaurasia, S., Singh, A. K., Songachan, L. S., Sharma, A. D., Bhardwaj, R., and Singh, K. (2020). Multi-locus genome-wide association studies reveal novel genomic regions associated with vegetative stage salt tolerance in bread wheat (Triticum aestivum L.). *Genomics* 112, 4608–4621. doi:10.1016/j.ygeno.2020.08.006.

Chen, J., Zhang, F., Zhao, C., Lv, G., Sun, C., Pan, Y., et al. (2019). Genome-wide association study of six quality traits reveals the association of the TaRPP13L1 gene with flour colour in Chinese bread wheat. *Plant Biotechnol. J.* 17, 2106–2122. doi:10.1111/pbi.13126.

Chen, S., Rouse, M. N., Zhang, W., Zhang, X., Guo, Y., Briggs, J., et al. (2020). Wheat gene Sr60 encodes a protein with two putative kinase domains that confers resistance to stem rust. *New Phytol.* 225, 948–959. doi:10.1111/nph.16169.

Clover, G. R. G., Ratti, C., and Henry, C. M. (2001). Molecular characterization and detection of European isolates of Soil-borne wheat mosaic virus. *Plant Pathol.* 50, 761–767. doi:10.1046/j.1365-3059.2001.00634.x.

Corsi B, Obinu L, Zanella CM, Cutrupi S, Day R, Geyer M, Lillemo M, Lin M, Mazza L, Percival-Alwyn L, Stadlmeier M, Mohler V, Hartl L, C. J. (2021). Identification of eight QTL controlling multiple yield components in a German multi-parental wheat population, including Rht24, WAPO-A1, WAPO-B1 and genetic loci on chromosomes 5A and 6A. *Theor. Appl. Genet.* doi:10.1007/s00122-021-03781-7.

Corsi, B., Percival-Alwyn, L., Downie, R. C., Venturini, L., Iagallo, E. M., Campos Mantello, C., et al. (2020). Genetic analysis of wheat sensitivity to the ToxB fungal effector from Pyrenophora tritici-repentis, the causal agent of tan spot. *Theor. Appl. Genet.* 133, 935–950. doi:10.1007/s00122-019-03517-8.

Crespo-Herrera, L. A. A., Akhunov, E., Garkava-Gustavsson, L., Jordan, K. W. W., Smith, C. M. M., Singh, R. P. P., et al. (2014). Mapping resistance to the bird cherry-oat aphid and the greenbug in wheat using sequence-based genotyping. *Theor. Appl. Genet.* 127, 1963–1973. doi:10.1007/s00122-014-2352-5.

Crespo-Herrera, L., Singh, R. P., Reynolds, M., and Huerta-Espino, J. (2019). Genetics of Greenbug Resistance in Synthetic Hexaploid Wheat Derived Germplasm. *Front. Plant Sci.* 10, 782. doi:10.3389/fpls.2019.00782.

Cui, F., Zhang, N., Fan, X. L., Zhang, W., Zhao, C. H., Yang, L. J., et al. (2017). Utilization of a Wheat660K SNP array-derived high-density genetic map for high-resolution mapping of a major QTL for kernel number. *Sci. Rep.* 7, 1–12. doi:10.1038/s41598-017-04028-6.

Dong, L., Wang, F., Liu, T., Dong, Z., Li, A., Jing, R., et al. (2014). Natural variation of TaGASR7-A1 affects grain length in common wheat under multiple cultivation conditions. *Mol. Breed.* 34, 937–947. doi:10.1007/s11032-014-0087-2.

Edae, E. A., Byrne, P. F., Haley, S. D., Lopes, M. S., and Reynolds, M. P. (2014). Genome-wide association mapping of yield and yield components of spring wheat under contrasting moisture regimes. *Theor. Appl. Genet.* 127, 791–807. doi:10.1007/s00122-013-2257-8.

Edae, E. A., Pumphrey, M. O., and Rouse, M. N. (2018). A genome-wide association study of field and seedling response to individual stem rust pathogen races reveals combinations of race-specific genes in north american spring wheat. *Front. Plant Sci.* 9, 1–18. doi:10.3389/fpls.2018.00052.

El-Feki, W. M., Byrne, P. F., Reid, S. D., and Haley, S. D. (2018). Mapping quantitative trait loci for agronomic traits inwinter wheat under different soil moisture levels. *Agronomy* 8, 133. doi:10.3390/agronomy8080133.

ElBasyoni, I., Saadalla, M., Baenziger, S., Bockelman, H., and Morsy, S. (2017). Cell Membrane Stability and Association Mapping for Drought and Heat Tolerance in a Worldwide Wheat Collection. *Sustainability* 9, 1606. doi:10.3390/su9091606.

Faris, J. D., Zhang, Z., Lu, H., Lu, S., Reddy, L., Cloutier, S., et al. (2010). A unique wheat disease resistance-like gene governs effector-triggered susceptibility to necrotrophic pathogens. *Proc. Natl. Acad. Sci.* 107, 13544–13549. doi:10.1073/pnas.1004090107.

Fazel-Najafabadi, M., Peng, J., Peairs, F. B., Simkova, H., Kilian, A., and Lapitan, N. L. V. (2015). Genetic mapping of resistance to Diuraphis noxia (Kurdjumov) biotype 2 in wheat (Triticum aestivum L.) accession CI2401. *Euphytica* 203, 607–614. doi:10.1007/s10681-014-1284-0.

Fu, D., Uauy, C., Distelfeld, A., Blechl, A., Epstein, L., Chen, X., et al. (2009). A kinase-START gene confers temperature-dependent resistance to wheat stripe rust. *Science (80-. ).* 323, 1357–1360. doi:10.1126/science.1166289.

Gahlaut, V., Jaiswal, V., Tyagi, B. S., Singh, G., Sareen, S., Balyan, H. S., et al. (2017). QTL mapping for nine drought-responsive agronomic traits in bread wheat under irrigated and rain-fed environments. *PLoS One* 12, e0182857. doi:10.1371/journal.pone.0182857.

Gálvez, S., Mérida-García, R., Camino, C., Borrill, P., Abrouk, M., Ramírez-González, R. H., et al. (2019). Hotspots in the genomic architecture of field drought responses in wheat as breeding targets. *Funct. Integr. Genomics* 19, 295–309. doi:10.1007/s10142-018-0639-3.

Genc, Y., Oldach, K., Verbyla, A. P., Lott, G., Hassan, M., Tester, M., et al. (2010). Sodium exclusion QTL associated with improved seedling growth in bread wheat under salinity stress. *Theor. Appl. Genet.* 121, 877–894. doi:10.1007/s00122-010-1357-y.

Genc, Y., Taylor, J., Rongala, J., and Oldach, K. (2014). A major locus for chloride accumulation on chromosome 5A in bread wheat. *PLoS One* 9, e98845. doi:10.1371/journal.pone.0098845.

Guo, J., Ren, Y., Tang, Z., Shi, W., and Zhou, M. (2019). Characterization and expression profiling of the ICE-CBF-COR genes in wheat. *PeerJ* 2019, 1–19. doi:10.7717/peerj.8190.

Guo, Y., Zhang, G., Guo, B., Qu, C., Zhang, M., Kong, F., et al. (2020). QTL mapping for quality traits using a highdensity genetic map of wheat. *PLoS One* 15, e0230601. doi:10.1371/journal.pone.0230601.

Gupta, P. K., Balyan, H. S., and Gahlaut, V. (2017). QTL analysis for drought tolerance in wheat: Present status and future possibilities. *Agronomy* 7, 5. doi:10.3390/agronomy7010005.

Gurung, S., Mamidi, S., Bonman, J. M., Xiong, M., Brown-Guedira, G., and Adhikari, T. B. (2014). Genome-wide association study reveals novel quantitative trait loci associated with resistance to multiple leaf spot diseases of spring wheat. *PLoS One* 9. doi:10.1371/journal.pone.0108179.

Harris, M O; Anderson, K; El-Bouhssini, M; Peairs, F; Hein, G; Xu, S. (2017). “Wheat pests: insects, mites, and prospects for the future,” in *Achieving sustainable cultivation of wheat Volume 1*, ed. P. Langridge (Burleigh Dodd Scientific Publishing Ltd), 467–543.

Hassan, F. S. C., Solouki, M., Fakheri, B. A., Nezhad, N. M., and Masoudi, B. (2018). Mapping QTLs for physiological and biochemical traits related to grain yield under control and terminal heat stress conditions in bread wheat (Triticum aestivum L.). *Physiol. Mol. Biol. Plants* 24, 1231–1243. doi:10.1007/s12298-018-0590-8.

He, S., Reif, J. C., Korzun, V., Bothe, R., Ebmeyer, E., and Jiang, Y. (2017). Genome-wide mapping and prediction suggests presence of local epistasis in a vast elite winter wheat populations adapted to Central Europe. *Theor. Appl. Genet.* 130, 635–647. doi:10.1007/s00122-016-2840-x.

Hiebert, C. W., Moscou, M. J., Hewitt, T., Steuernagel, B., Hernández-Pinzón, I., Green, P., et al. (2020). Stem rust resistance in wheat is suppressed by a subunit of the mediator complex. *Nat. Commun.* 11, 1–10. doi:10.1038/s41467-020-14937-2.

Hourcade, D., Bogard, M., Bonnefoy, M., Savignard, F., Mohamadi, F., Lafarge, S., et al. (2019). Genome-wide association analysis of resistance to wheat spindle streak mosaic virus in bread wheat. *Plant Pathol.* 68, 609–616. doi:10.1111/ppa.12972.

Hu, M. J., Zhang, H. P., Cao, J. J., Zhu, X. F., Wang, S. X., Jiang, H., et al. (2016a). Characterization of an IAA-glucose hydrolase gene TaTGW6 associated with grain weight in common wheat (Triticum aestivum L.). *Mol. Breed.* 36, 1–11. doi:10.1007/s11032-016-0449-z.

Hu, M. J., Zhang, H. P., Liu, K., Cao, J. J., Wang, S. X., Jiang, H., et al. (2016b). Cloning and characterization of TaTGW-7A gene associated with grain weight in wheat via SLAF-seq-BSA. *Front. Plant Sci.* 7, 1902. doi:10.3389/fpls.2016.01902.

Hu, P., Zheng, Q., Luo, Q., Teng, W., Li, H., Li, B., et al. (2021). Genome-wide association study of yield and related traits in common wheat under salt-stress conditions. *BMC Plant Biol.* 21, 27. doi:10.1186/s12870-020-02799-1.

Huang, L., Brooks, S. A., Li, W., Fellers, J. P., Trick, H. N., and Gill, B. S. (2003). Map-based cloning of leaf rust resistance gene Lr21 from the large and polyploid genome of bread wheat. *Genetics* 164, 655–664. Available at: https://www.genetics.org/content/164/2/655.long [Accessed January 22, 2021].

Hurni, S., Brunner, S., Buchmann, G., Herren, G., Jordan, T., Krukowski, P., et al. (2013). Rye Pm8 and wheat Pm3 are orthologous genes and show evolutionary conservation of resistance function against powdery mildew. *Plant J.* 76, 957–969. doi:10.1111/tpj.12345.

Hussain, B. (2015). Modernization in plant breeding approaches for improving biotic stress resistance in crop plants. *TURKISH J. Agric. For.* 39, 515–530. doi:10.3906/tar-1406-176.

Hussain, B., Lucas, S. J., Ozturk, L., and Budak, H. (2017a). Mapping QTLs conferring salt tolerance and micronutrient concentrations at seedling stagein wheat. *Sci. Rep.* 7, 15662. doi:10.1038/s41598-017-15726-6.

Hussain, W., Stephen Baenziger, P., Belamkar, V., Guttieri, M. J., Venegas, J. P., Easterly, A., et al. (2017b). Genotyping-by-Sequencing Derived High-Density Linkage Map and its Application to QTL Mapping of Flag Leaf Traits in Bread Wheat. *Sci. Rep.* 7, 1–15. doi:10.1038/s41598-017-16006-z.

Iehisa, J. C. M., Matsuura, T., Mori, I. C., Yokota, H., Kobayashi, F., and Takumi, S. (2014). Identification of quantitative trait loci for abscisic acid responsiveness in the D-genome of hexaploid wheat. *J. Plant Physiol.* 171, 830–841. doi:10.1016/j.jplph.2014.02.003.

Jamil, M., Ali, A., Gul, A., Ghafoor, A., Napar, A. A., Ibrahim, A. M. H., et al. (2019). Genome-wide association studies of seven agronomic traits under two sowing conditions in bread wheat. *BMC Plant Biol.* 19, 149. doi:10.1186/s12870-019-1754-6.

Jiang, C., Kan, J., Ordon, F., Perovic, D., and Yang, P. (2020). Bymovirus-induced yellow mosaic diseases in barley and wheat: viruses, genetic resistances and functional aspects. *Theor. Appl. Genet.* 133, 1623–1640. doi:10.1007/s00122-020-03555-7.

Jin, H., Wen, W., Liu, J., Zhai, S., Zhang, Y., Yan, J., et al. (2016). Genome-Wide QTL Mapping for Wheat Processing Quality Parameters in a Gaocheng 8901/Zhoumai 16 Recombinant Inbred Line Population. *Front. Plant Sci.* 7. doi:10.3389/fpls.2016.01032.

Jin, J., Liu, D., Qi, Y., Ma, J., and Zhen, W. (2020). Major QTL for Seven Yield-Related Traits in Common Wheat (Triticum aestivum L.). *Front. Genet.* 11, 1012. doi:10.3389/fgene.2020.01012.

Jin, Y., Zhai, S., Wang, W., Ding, X., Guo, Z., Bai, L., et al. (2018). Identification of genes from the ICE–CBF–COR pathway under cold stress in Aegilops–Triticum composite group and the evolution analysis with those from Triticeae. *Physiol. Mol. Biol. Plants* 24, 211–229. doi:10.1007/s12298-017-0495-y.

Kang, Y., Barry, K., Cao, F., and Zhou, M. (2020). Genome-wide association mapping for adult resistance to powdery mildew in common wheat. *Mol. Biol. Rep.* 47, 1241–1256. doi:10.1007/s11033-019-05225-4.

Kassa, M. T., Haas, S., Schliephake, E., Lewis, C., You, F. M., Pozniak, C. J., et al. (2016). A saturated SNP linkage map for the orange wheat blossom midge resistance gene Sm1. *Theor. Appl. Genet.* 129, 1507–1517. doi:10.1007/s00122-016-2720-4.

Khalaf, L., Chuang, W. P., Aguirre-Rojas, L. M., Klein, P., and Michael Smith, C. (2019). Differences in Aceria tosichella population responses to wheat resistance genes and wheat virus transmission. *Arthropod. Plant. Interact.* 13, 807–818. doi:10.1007/s11829-019-09717-9.

Klymiuk, V., Yaniv, E., Huang, L., Raats, D., Fatiukha, A., Chen, S., et al. (2018). Cloning of the wheat Yr15 resistance gene sheds light on the plant tandem kinase-pseudokinase family. *Nat. Commun.* 9, 1–12. doi:10.1038/s41467-018-06138-9.

Kollers, S., Rodemann, B., Ling, J., Korzun, V., Ebmeyer, E., Argillier, O., et al. (2014). Genome-wide association mapping of tan spot resistance (Pyrenophora tritici-repentis) in European winter wheat. *Mol. Breed.* 34, 363–371. doi:10.1007/s11032-014-0039-x.

Krattinger, S. G., Lagudah, E. S., Spielmeyer, W., Singh, R. P., Huerta-Espino, J., McFadden, H., et al. (2009). A Putative ABC Transporter Confers Durable Resistance to Multiple Fungal Pathogens in Wheat. *Science (80-. ).* 323, 1360–1363. doi:10.1126/science.1166453.

Kuzay, S., Xu, Y., Zhang, J., Katz, A., Pearce, S., Su, Z., et al. (2019). Identification of a candidate gene for a QTL for spikelet number per spike on wheat chromosome arm 7AL by high-resolution genetic mapping. *Theor. Appl. Genet.* 132, 2689–2705. doi:10.1007/s00122-019-03382-5.

Li, G., Xu, X., Carver, B. F., Guo, P., and Puterka, G. (2018). Dn10 , a New Gene Conferring Resistance to Russian Wheat Aphid Biotype 2 in Iranian Wheat Landrace PI 682675. *Crop Sci.* 58, 1219–1225. doi:10.2135/cropsci2017.10.0649.

Li, M., Dong, L., Li, B., Wang, Z., Xie, J., Qiu, D., et al. (2020). A CNL protein in wild emmer wheat confers powdery mildew resistance. *New Phytol.* 228, 1027–1037. doi:10.1111/nph.16761.

Liu, C., Khodaee, M., Lopes, M. S., Sansaloni, C., Dreisigacker, S., Sukumaran, S., et al. (2019a). Multi-environment QTL analysis using an updated genetic map of a widely distributed Seri × Babax spring wheat population. *Mol. Breed.* 39, 1–15. doi:10.1007/s11032-019-1040-1.

Liu, C., Sukumaran, S., Claverie, E., Sansaloni, C., Dreisigacker, S., and Reynolds, M. (2019b). Genetic dissection of heat and drought stress QTLs in phenology-controlled synthetic-derived recombinant inbred lines in spring wheat. *Mol. Breed.* 39, 1–18. doi:10.1007/s11032-019-0938-y.

Liu, J., He, Z., Rasheed, A., Wen, W., Yan, J., Zhang, P., et al. (2017a). Genome-wide association mapping of black point reaction in common wheat (Triticum aestivum L.). *BMC Plant Biol.* 17, 220. doi:10.1186/s12870-017-1167-3.

Liu, N., Bai, G., Lin, M., Xu, X., and Zheng, W. (2017b). Genome-wide Association Analysis of Powdery Mildew Resistance in U.S. Winter Wheat. *Sci. Rep.* 7, 1–11. doi:10.1038/s41598-017-11230-z.

Liu, S., Bai, G., Lin, M., Luo, M., Zhang, D., Jin, F., et al. (2020). Identification of candidate chromosome region of Sbwm1 for Soil-borne wheat mosaic virus resistance in wheat. *Sci. Rep.* 10, 1–11. doi:10.1038/s41598-020-64993-3.

Liu, S., Yang, X., Zhang, D., Bai, G., Chao, S., and Bockus, W. (2014). Genome-wide association analysis identified SNPs closely linked to a gene resistant to Soil-borne wheat mosaic virus. *Theor. Appl. Genet.* 127, 1039–1047. doi:10.1007/s00122-014-2277-z.

Lopes, M. S., Dreisigacker, S., Peña, R. J., Sukumaran, S., and Reynolds, M. P. (2015). Genetic characterization of the wheat association mapping initiative (WAMI) panel for dissection of complex traits in spring wheat. *Theor. Appl. Genet.* 128, 453–464. doi:10.1007/s00122-014-2444-2.

Lu, P., Guo, L., Wang, Z., Li, B., Li, J., Li, Y., et al. (2020). A rare gain of function mutation in a wheat tandem kinase confers resistance to powdery mildew. *Nat. Commun.* 11, 680. doi:10.1038/s41467-020-14294-0.

Lucas, S. J., Salantur, A., Yazar, S., and Budak, H. (2017). High-throughput SNP genotyping of modern and wild emmer wheat for yield and root morphology using a combined association and linkage analysis. *Funct. Integr. Genomics* 17, 667–685. doi:10.1007/s10142-017-0563-y.

Luo, M., Xie, L., Chakraborty, S., Wang, A., Matny, O., Jugovich, M., et al. (2021). A five-transgene cassette confers broad-spectrum resistance to a fungal rust pathogen in wheat. *Nat. Biotechnol.*, 1–6. doi:10.1038/s41587-020-00770-x.

Ma, D., Yan, J., He, Z., Wu, L., and Xia, X. (2012). Characterization of a cell wall invertase gene TaCwi-A1 on common wheat chromosome 2A and development of functional markers. *Mol. Breed.* 29, 43–52. doi:10.1007/s11032-010-9524-z.

Maccaferri, M., Zhang, J., Bulli, P., Abate, Z., Chao, S., Cantu, D., et al. (2015). A genome-wide association study of resistance to stripe rust (Puccinia striiformis f. sp. tritici) in a worldwide collection of hexaploid spring wheat (Triticum aestivum L.). *G3 Genes, Genomes, Genet.* 5, 449–465. doi:10.1534/g3.114.014563.

Marchal, C., Zhang, J., Zhang, P., Fenwick, P., Steuernagel, B., Adamski, N. M., et al. (2018). BED-domain-containing immune receptors confer diverse resistance spectra to yellow rust. *Nat. Plants* 4, 662–668. doi:10.1038/s41477-018-0236-4.

McIntosh, R. A., Dubcovsky, J., Rogers, W. J., Xia, X. C., and Raupp, W. J. CATALOGUE OF GENE SYMBOLS FOR WHEAT: 2019 SUPPLEMENT. *A n n u a l W h e a t N e ws Let t e r*. Available at: https://wheat.pw.usda.gov/GG3/sites/default/files/Catalogue of Gene Symbols for Wheat - supplement2019.pdf.

Mihalyov, P. D., Nichols, V. A., Bulli, P., Rouse, M. N., and Pumphrey, M. O. (2017). Multi‐Locus Mixed Model Analysis Of Stem Rust Resistance In Winter Wheat. *Plant Genome* 10. doi:10.3835/plantgenome2017.01.0001.

Molero, G., Joynson, R., Pinera-chavez, F. J., Gardiner, L., Rivera-amado, C., Hall, A., et al. (2018). Elucidating the genetic basis of biomass accumulation and radiation use efficiency in spring wheat and its role in yield potential. 52. doi:10.1109/MC.2009.260.

Mondal, S., Mason, R. E., Huggins, T., and Hays, D. B. (2015). QTL on wheat (Triticum aestivum L.) chromosomes 1B, 3D and 5A are associated with constitutive production of leaf cuticular wax and may contribute to lower leaf temperatures under heat stress. *Euphytica* 201, 123–130. doi:10.1007/s10681-014-1193-2.

Moore, J. W., Herrera-Foessel, S., Lan, C., Schnippenkoetter, W., Ayliffe, M., Huerta-Espino, J., et al. (2015). A recently evolved hexose transporter variant confers resistance to multiple pathogens in wheat. *Nat. Genet.* 47, 1494–1498. doi:10.1038/ng.3439.

Mourad, A. M. I., Sallam, A., Belamkar, V., Wegulo, S., Bowden, R., Jin, Y., et al. (2018). Genome-wide association study for identification and validation of novel snp markers for Sr6 stem rust resistance gene in bread wheat. *Front. Plant Sci.* 9, 1–12. doi:10.3389/fpls.2018.00380.

Muleta, K. T., Rouse, M. N., Rynearson, S., Chen, X., Buta, B. G., and Pumphrey, M. O. (2017). Characterization of molecular diversity and genome-wide mapping of loci associated with resistance to stripe rust and stem rust in Ethiopian bread wheat accessions. *BMC Plant Biol.* 17, 134. doi:10.1186/s12870-017-1082-7.

Nedelkou, I.-P., Maurer, A., Schubert, A., Léon, J., and Pillen, K. (2017). Exotic QTL improve grain quality in the tri-parental wheat population SW84. *PLoS One* 12, e0179851. doi:10.1371/journal.pone.0179851.

Nelson, R., Wiesner-Hanks, T., Wisser, R., and Balint-Kurti, P. (2018). Navigating complexity to breed disease-resistant crops. *Nat. Rev. Genet.* 19, 21–33. doi:10.1038/nrg.2017.82.

Nemri, A., Saunders, D. G. O., Anderson, C., Upadhyaya, N. M., Win, J., Lawrence, G. J., et al. (2014). The genome sequence and effector complement of the flax rust pathogen Melampsora lini. *Front. Plant Sci.* 5. doi:10.3389/fpls.2014.00098.

Oyiga, B. C., Sharma, R. C., Baum, M., Ogbonnaya, F. C., Léon, J., and Ballvora, A. (2018). Allelic variations and differential expressions detected at quantitative trait loci for salt stress tolerance in wheat. *Plant. Cell Environ.* 41, 919–935. doi:10.1111/pce.12898.

Pang, Y., Liu, C., Wang, D., St. Amand, P., Bernardo, A., Li, W., et al. (2020). High-Resolution Genome-wide Association Study Identifies Genomic Regions and Candidate Genes for Important Agronomic Traits in Wheat. *Mol. Plant* 13, 1311–1327. doi:10.1016/j.molp.2020.07.008.

Periyannan, S., Moore, J., Ayliffe, M., Bansal, U., Wang, X., Huang, L., et al. (2013). The Gene Sr33, an Ortholog of Barley Mla Genes, Encodes Resistance to Wheat Stem Rust Race Ug99. *Science (80-. ).* 341, 786–788. doi:10.1126/science.1239028.

Perovic, D., Förster, J., Devaux, P., Hariri, D., Guilleroux, M., Kanyuka, K., et al. (2009). Mapping and diagnostic marker development for Soil-borne cereal mosaic virus resistance in bread wheat. *Mol. Breed.* 23, 641–653. doi:10.1007/s11032-009-9262-2.

Pinto, R. S., Lopes, M. S., Collins, N. C., and Reynolds, M. P. (2016). Modelling and genetic dissection of staygreen under heat stress. *Theor. Appl. Genet.* 129, 2055–2074. doi:10.1007/s00122-016-2757-4.

Pinto, R. S., and Reynolds, M. P. (2015). Common genetic basis for canopy temperature depression under heat and drought stress associated with optimized root distribution in bread wheat. *Theor. Appl. Genet.* 128, 575–585. doi:10.1007/s00122-015-2453-9.

Quraishi, U. M., Pont, C., Ain, Q., Flores, R., Burlot, L., Alaux, M., et al. (2017). Combined Genomic and Genetic Data Integration of Major Agronomical Traits in Bread Wheat (Triticum aestivum L.). *Front. Plant Sci.* 8. doi:10.3389/fpls.2017.01843.

Raman, R., Allen, H., Diffey, S., Raman, H., Martin, P., and McKelvie, K. (2009). Localisation of quantitative trait loci for quality attributes in a doubled haploid population of wheat (Triticum aestivum L.). *Genome* 52, 701–715. doi:10.1139/G09-045.

Ravel, C., Faye, A., Ben-Sadoun, S., Ranoux, M., Dardevet, M., Dupuits, C., et al. (2020). SNP markers for early identification of high molecular weight glutenin subunits (HMW-GSs) in bread wheat. *Theor. Appl. Genet.* 133, 751–770. doi:10.1007/s00122-019-03505-y.

Saintenac, C., Cambon, F., Aouini, L., Verstappen, E., Ghaffary, S. M. T., Poucet, T., et al. (2021). A wheat cysteine-rich receptor-like kinase confers broad-spectrum resistance against Septoria tritici blotch. *Nat. Commun.* 12, 433. doi:10.1038/s41467-020-20685-0.

Saintenac, C., Lee, W.-S., Cambon, F., Rudd, J. J., King, R. C., Marande, W., et al. (2018). Wheat receptor-kinase-like protein Stb6 controls gene-for-gene resistance to fungal pathogen Zymoseptoria tritici. *Nat. Genet.* 50, 368–374. doi:10.1038/s41588-018-0051-x.

Saintenac, C., Zhang, W., Salcedo, A., Rouse, M. N., Trick, H. N., Akhunov, E., et al. (2013). Identification of Wheat Gene Sr35 That Confers Resistance to Ug99 Stem Rust Race Group. *Science (80-. ).* 341, 783–786. doi:10.1126/science.1239022.

Sakuma, S., Golan, G., Guo, Z., Ogawa, T., Tagiri, A., Sugimoto, K., et al. (2019). Unleashing floret fertility in wheat through the mutation of a homeobox gene. *Proc. Natl. Acad. Sci. U. S. A.* 116, 5182–5187. doi:10.1073/pnas.1815465116.

Sánchez-Martín, J., Steuernagel, B., Ghosh, S., Herren, G., Hurni, S., Adamski, N., et al. (2016). Rapid gene isolation in barley and wheat by mutant chromosome sequencing. *Genome Biol.* 17, 221. doi:10.1186/s13059-016-1082-1.

Sehgal, D., Autrique, E., Singh, R., Ellis, M., Singh, S., and Dreisigacker, S. (2017). Identification of genomic regions for grain yield and yield stability and their epistatic interactions. *Sci. Rep.* 7, 1–12. doi:10.1038/srep41578.

Sehgal, D., Mondal, S., Crespo-Herrera, L., Velu, G., Juliana, P., Huerta-Espino, J., et al. (2020a). Haplotype-Based, Genome-Wide Association Study Reveals Stable Genomic Regions for Grain Yield in CIMMYT Spring Bread Wheat. *Front. Genet.* 11, 1427. doi:10.3389/fgene.2020.589490.

Sehgal, D., Rosyara, U., Mondal, S., Singh, R., Poland, J., and Dreisigacker, S. (2020b). Incorporating Genome-Wide Association Mapping Results Into Genomic Prediction Models for Grain Yield and Yield Stability in CIMMYT Spring Bread Wheat. *Front. Plant Sci.* 11, 197. doi:10.3389/fpls.2020.00197.

Shahinnia, F., Le Roy, J., Laborde, B., Sznajder, B., Kalambettu, P., Mahjourimajd, S., et al. (2016). Genetic association of stomatal traits and yield in wheat grown in low rainfall environments. *BMC Plant Biol.* 16, 150. doi:10.1186/s12870-016-0838-9.

Sharma, D. K., Torp, A. M., Rosenqvist, E., Ottosen, C.-O., and Andersen, S. B. (2017). QTLs and Potential Candidate Genes for Heat Stress Tolerance Identified from the Mapping Populations Specifically Segregating for Fv/Fm in Wheat. *Front. Plant Sci.* 8, 1668. doi:10.3389/fpls.2017.01668.

Sharma, D., Singh, R., Rane, J., Gupta, V. K., Mamrutha, H. M., and Tiwari, R. (2016). Mapping quantitative trait loci associated with grain filling duration and grain number under terminal heat stress in bread wheat ( Triticum aestivum L.). *Plant Breed.* 135, 538–545. doi:10.1111/pbr.12405.

Shi, G., Zhang, Z., Friesen, T. L., Raats, D., Fahima, T., Brueggeman, R. S., et al. (2016). The hijacking of a receptor kinase–driven pathway by a wheat fungal pathogen leads to disease. *Sci. Adv.* 2, e1600822. doi:10.1126/sciadv.1600822.

Shi, S., Azam, F. I., Li, H., Chang, X., Li, B., and Jing, R. (2017). Mapping QTL for stay-green and agronomic traits in wheat under diverse water regimes. *Euphytica* 213, 1–19. doi:10.1007/s10681-017-2002-5.

Shokat, S., Sehgal, D., Vikram, P., Liu, F., and Singh, S. (2020). Molecular markers associated with agro-physiological traits under terminal drought conditions in bread wheat. *Int. J. Mol. Sci.* 21, 3156. doi:10.3390/ijms21093156.

Shukla, S., Singh, K., Patil, R. V., Kadam, S., Bharti, S., Prasad, P., et al. (2015). Genomic regions associated with grain yield under drought stress in wheat (Triticum aestivum L.). *Euphytica* 203, 449–467. doi:10.1007/s10681-014-1314-y.

Simons, K., Anderson, J. A., Mergoum, M., Faris, J. D., Klindworth, D. L., Xu, S. S., et al. (2012). Genetic Mapping Analysis of Bread-Making Quality Traits in Spring Wheat. *Crop Sci.* 52, 2182. doi:10.2135/cropsci2012.03.0175.

Singh, R. P., Singh, P. K., Rutkoski, J., Hodson, D. P., He, X., Jørgensen, L. N., et al. (2016). Disease Impact on Wheat Yield Potential and Prospects of Genetic Control. *Annu. Rev. Phytopathol.* 54, 303–322. doi:10.1146/annurev-phyto-080615-095835.

Smith, C. M. (2021). Conventional breeding of insect-resistant crop plants: still the best way to feed the world population. *Curr. Opin. Insect Sci.* 45, 7–13. doi:10.1016/j.cois.2020.11.008.

Soriano, J. M., and Alvaro, F. (2019). Discovering consensus genomic regions in wheat for root-related traits by QTL meta-analysis. *Sci. Rep.* 9, 1–14. doi:10.1038/s41598-019-47038-2.

Steuernagel, B., Periyannan, S. K., Hernández-Pinzón, I., Witek, K., Rouse, M. N., Yu, G., et al. (2016). Rapid cloning of disease-resistance genes in plants using mutagenesis and sequence capture. *Nat. Biotechnol.* 34, 652–655. doi:10.1038/nbt.3543.

Su, Z., Bernardo, A., Tian, B., Chen, H., Wang, S., Ma, H., et al. (2019). A deletion mutation in TaHRC confers Fhb1 resistance to Fusarium head blight in wheat. *Nat. Genet.* 51, 1099–1105. doi:10.1038/s41588-019-0425-8.

Su, Z., Hao, C., Wang, L., Dong, Y., and Zhang, X. (2011). Identification and development of a functional marker of TaGW2 associated with grain weight in bread wheat (Triticum aestivum L.). *Theor. Appl. Genet.* 122, 211–223. doi:10.1007/s00122-010-1437-z.

Su, Z., Jin, S., Lu, Y., Zhang, G., Chao, S., and Bai, G. (2016). Single nucleotide polymorphism tightly linked to a major QTL on chromosome 7A for both kernel length and kernel weight in wheat. *Mol. Breed.* 36. doi:10.1007/s11032-016-0436-4.

Sukumaran, S., Dreisigacker, S., Lopes, M., Chavez, P., and Reynolds, M. P. (2015). Genome-wide association study for grain yield and related traits in an elite spring wheat population grown in temperate irrigated environments. *Theor. Appl. Genet.* 128, 353–363. doi:10.1007/s00122-014-2435-3.

Sukumaran, S., Lopes, M. S., Dreisigacker, S., Dixon, L. E., Zikhali, M., Griffiths, S., et al. (2016). Identification of earliness per se flowering time locus in spring wheat through a genome-wide association study. *Crop Sci.* 56, 2962–2972. doi:10.2135/cropsci2016.01.0066.

Sun, H., Lu, J., Fan, Y., Zhao, Y., Kong, F., Li, R., et al. (2008). Quantitative trait loci (QTLs) for quality traits related to protein and starch in wheat. *Prog. Nat. Sci.* 18, 825–831. doi:10.1016/j.pnsc.2007.12.013.

Suzuki, T., Murai, M. N., Hayashi, T., Nasuda, S., Yoshimura, Y., and Komatsuda, T. (2015). Resistance to wheat yellow mosaic virus in Madsen wheat is controlled by two major complementary QTLs. *Theor. Appl. Genet.* 128, 1569–1578. doi:10.1007/s00122-015-2532-y.

Tadesse, W., Suleiman, S., Tahir, I., Sanchez-Garcia, M., Jighly, A., Hagras, A., et al. (2019). Heat-tolerant QTLs associated with grain yield and its components in spring bread wheat under heat-stressed environments of Sudan and Egypt. *Crop Sci.* 59, 199–211. doi:10.2135/cropsci2018.06.0389.

Talukder, S. K., Babar, M. A., Vijayalakshmi, K., Poland, J., Prasad, P. V. V., Bowden, R., et al. (2014). Mapping QTL for the traits associated with heat tolerance in wheat (Triticum aestivumL.). *BMC Genet.* 15, 97. doi:10.1186/s12863-014-0097-4.

Tan, C.-T., Yu, H., Yang, Y., Xu, X., Chen, M., Rudd, J. C., et al. (2017). Development and validation of KASP markers for the greenbug resistance gene *Gb7* and the Hessian fly resistance gene *H32* in wheat. *Theor. Appl. Genet.* 130, 1867–1884. doi:10.1007/s00122-017-2930-4.

Tolmay, V. L., Sydenham, S. L., Sikhakhane, T. N., Nhlapho, B. N., and Tsilo, T. J. (2020). Elusive diagnostic markers for Russian wheat aphid resistance in bread wheat: Deliberating and reviewing the status quo. *Int. J. Mol. Sci.* 21, 1–22. doi:10.3390/ijms21218271.

Uauy, C., Distelfeld, A., Fahima, T., Blechl, A., and Dubcovsky, J. (2006). A NAC gene regulating senescence improves grain protein, zinc, and iron content in wheat. *Science (80-. ).* 314, 1298–1301. doi:10.1126/science.1133649.

Valluru, R., Reynolds, M. P., Davies, W. J., and Sukumaran, S. (2017). Phenotypic and genome-wide association analysis of spike ethylene in diverse wheat genotypes under heat stress. *New Phytol.* 214, 271–283. doi:10.1111/nph.14367.

Voss-Fels, K. P., Keeble-Gagnère, G., Hickey, L. T., Tibbits, J., Nagornyy, S., Hayden, M. J., et al. (2019). High-resolution mapping of rachis nodes per rachis, a critical determinant of grain yield components in wheat. *Theor. Appl. Genet.* 132, 2707–2719. doi:10.1007/s00122-019-03383-4.

Wang, H., Sun, S., Ge, W., Zhao, L., Hou, B., Wang, K., et al. (2020a). Horizontal gene transfer of Fhb7 from fungus underlies Fusarium head blight resistance in wheat. *Science (80-. ).* 368. doi:10.1126/science.aba5435.

Wang, H., Zou, S., Li, Y., Lin, F., and Tang, D. (2020b). An ankyrin-repeat and WRKY-domain-containing immune receptor confers stripe rust resistance in wheat. *Nat. Commun.* 11, 1–11. doi:10.1038/s41467-020-15139-6.

Wang, S., JIA, S., SUN, D., FAN, H., CHANG, X., and JING, R. (2016). Mapping QTLs for stomatal density and size under drought stress in wheat (Triticum aestivum L.). *J. Integr. Agric.* 15, 1955–1967. doi:10.1016/S2095-3119(15)61264-3.

Ward, B. P., Brown-Guedira, G., Kolb, F. L., Van Sanford, D. A., Tyagi, P., Sneller, C. H., et al. (2019). Genome-wide association studies for yield-related traits in soft red winter wheat grown in Virginia. *PLoS One* 14, e0208217. doi:10.1371/journal.pone.0208217.

Xie, J., Guo, G., Wang, Y., Hu, T., Wang, L., Li, J., et al. (2020). A rare single nucleotide variant in Pm5e confers powdery mildew resistance in common wheat. *New Phytol.* 228, 1011–1026. doi:10.1111/nph.16762.

Xing, L., Hu, P., Liu, J., Witek, K., Zhou, S., Xu, J., et al. (2018). Pm21 from Haynaldia villosa Encodes a CC-NBS-LRR Protein Conferring Powdery Mildew Resistance in Wheat. *Mol. Plant* 11, 874–878. doi:10.1016/j.molp.2018.02.013.

Xu, X., Li, G., Carver, B. F., and Armstrong, J. S. (2020). Gb8, a new gene conferring resistance to economically important greenbug biotypes in wheat. *Theor. Appl. Genet.* 133, 615–622. doi:10.1007/s00122-019-03491-1.

Xu, Y. F., Li, S. S., Li, L. H., Ma, F. F., Fu, X. Y., Shi, Z. L., et al. (2017). QTL mapping for yield and photosynthetic related traits under different water regimes in wheat. *Mol. Breed.* 37, 1–18. doi:10.1007/s11032-016-0583-7.

Yahiaoui, N., Srichumpa, P., Dudler, R., and Keller, B. (2004). Genome analysis at different ploidy levels allows cloning of the powdery mildew resistance gene Pm3b from hexaploid wheat. *Plant J.* 37, 528–538. doi:10.1046/j.1365-313X.2003.01977.x.

Yan, L., Loukoianov, A., Tranquilli, G., Helguera, M., Fahima, T., and Dubcovsky, J. (2003). Positional cloning of the wheat vernalization gene VRN1. *Proc. Natl. Acad. Sci. U. S. A.* 100, 6263–6268. doi:10.1073/pnas.0937399100.

Yang, Y., Basnet, B. R., Ibrahim, A. M. H., Rudd, J. C., Chen, X., Bowden, R. L., et al. (2019). Developing KASP Markers on a Major Stripe Rust Resistance QTL in a Popular Wheat TAM 111 Using 90K Array and Genotyping-by-Sequencing SNPs. *Crop Sci.* 59, 165–175. doi:10.2135/cropsci2018.05.0349.

Yang, Y., Chai, Y., Zhang, X., Lu, S., Zhao, Z., Wei, D., et al. (2020a). Multi-Locus GWAS of Quality Traits in Bread Wheat: Mining More Candidate Genes and Possible Regulatory Network. *Front. Plant Sci.* 11, 1091. doi:10.3389/fpls.2020.01091.

Yang, Y., Dhakal, S., Chu, C., Wang, S., Xue, Q., Rudd, J. C., et al. (2020b). Genome wide identification of QTL associated with yield and yield components in two popular wheat cultivars TAM 111 and TAM 112. *PLoS One* 15, e0237293. doi:10.1371/journal.pone.0237293.

Yu, L. X., Chao, S., Singh, R. P., and Sorrells, M. E. (2017). Identification and validation of single nucleotide polymorphic markers linked to Ug99 stem rust resistance in spring wheat. *PLoS One* 12, 1–21. doi:10.1371/journal.pone.0171963.

Yu, S., Wu, J., Wang, M., Shi, W., Xia, G., Jia, J., et al. (2020). Haplotype variations in QTL for salt tolerance in Chinese wheat accessions identified by marker-based and pedigree-based kinship analyses. *Crop J.* 8, 1011–1024. doi:10.1016/j.cj.2020.03.007.

Zanke, C. D., Ling, J., Plieske, J., Kollers, S., Ebmeyer, E., Korzun, V., et al. (2015). Analysis of main effect QTL for thousand grain weight in European winter wheat (Triticum aestivum L.) by genome-wide association mapping. *Front. Plant Sci.* 6, 1–14. doi:10.3389/fpls.2015.00644.

Zhang, H., Mao, X., Zhang, J., Chang, X., and Jing, R. (2013). Single-nucleotide polymorphisms and association analysis of drought-resistance gene TaSnRK2.8 in common wheat. *Plant Physiol. Biochem.* 70, 174–181. doi:10.1016/j.plaphy.2013.04.010.

Zhang, Y., Liu, J., Xia, X., and He, Z. (2014). TaGS-D1, an ortholog of rice OsGS3, is associated with grain weight and grain length in common wheat. *Mol. Breed.* 34, 1097–1107. doi:10.1007/s11032-014-0102-7.

Zhao, L., Abdelsalam, N. R., Xu, Y., Chen, M. S., Feng, Y., Kong, L., et al. (2020). Identification of two novel Hessian fly resistance genes H35 and H36 in a hard winter wheat line SD06165. *Theor. Appl. Genet.* 133, 2343–2353. doi:10.1007/s00122-020-03602-3.

Zou, S., Wang, H., Li, Y., Kong, Z., and Tang, D. (2018). The NB-LRR gene Pm60 confers powdery mildew resistance in wheat. *New Phytol.* 218, 298–309. doi:10.1111/nph.14964.
